# Effectiveness and safety of acupuncture for post-stroke spasticity: A systematic review and meta-analysis

**DOI:** 10.3389/fneur.2022.942597

**Published:** 2022-08-17

**Authors:** Chen Xue, Chengzhi Jiang, Yuanyuan Zhu, Xiaobo Liu, Dongling Zhong, Yuxi Li, Huiling Zhang, Wenjing Tang, Jian She, Cheng Xie, Juan Li, Yue Feng, Rongjiang Jin

**Affiliations:** ^1^School of Health Preservation and Rehabilitation, Chengdu University of Traditional Chinese Medicine, Chengdu, China; ^2^Department of Rehabilitation Medicine, Sichuan Science City Hospital, Mianyang, China; ^3^Department of Rehabilitation Medicine, Integrated Traditional Chinese and Western Medicine Hospital of Panzhihua City, Panzhihua, China; ^4^The Third Hospital/Acupuncture and Tuina School, Chengdu University of Traditional Chinese Medicine, Chengdu, China

**Keywords:** acupuncture, spasticity, stroke, systematic review, meta-analysis

## Abstract

**Objective:**

This systematic review and meta-analysis aimed to comprehensively evaluate the effectiveness and safety of acupuncture for post-stroke spasticity.

**Methods:**

Nine electronic databases were searched from their inception to 6 June 2022, to identify randomized-controlled trials (RCTs) that investigated the effectiveness and safety of acupuncture for post-stroke spasticity. Two reviewers independently screened the studies, extracted the data, assessed the risk of bias. The reporting quality of interventions in controlled trials of acupuncture was evaluated using Revised Standards for Reporting Interventions in Clinical Trials of Acupuncture (STRICTA). The RevMan 5.4 and R 4.2.0 software were used for statistical analysis.

**Results:**

A total of 88 eligible studies were included, involving 6,431 individuals. The pooled data demonstrated that acupuncture combined with conventional rehabilitation (CR) was superior to CR in reducing the Modified Ashworth Scale (MAS) score (standardized mean difference [SMD] = −0.73; 95% CI = −0.83 to −0.63; *I*^2^ = 65%; low certainty of evidence). The favorable results were also observed in comparisons of acupuncture vs. CR (SMD = −0.22, 95% CI = −0.36 to −0.07; *I*^2^ = 49%; moderate certainty of evidence). Subgroup analysis showed that acupuncture treatment with a frequency of once or twice a day was more effective than CR. In addition, the antispasmodic effect of acupuncture treatment increased with more sessions. Four studies explicitly reported slight acupuncture-related adverse events.

**Conclusion:**

Acupuncture could be recommended as adjuvant therapy for spasticity after stroke. However, due to the high risk of bias and heterogeneity of the included studies, the effectiveness of acupuncture for post-stroke spasticity remains to be confirmed.

## Introduction

Spasticity is one of the most common complications after stroke with a prevalence of 30–80% (1). As a motor dysfunction after the central nervous system lesions, spasticity is characterized by a velocity-dependent increase in tonic stretch reflex with exaggerated tendon jerks (2). Spasticity often results in several clinical symptoms, including joint contractures, deformities, swelling, and pain, which severely limits the motor functions of patients with stroke (3). Moreover, the presence of spasticity may interfere with self-care ability of patients, reduce their quality of life and lead to depressive symptoms (4). More importantly, spasticity brings a heavy financial burden to families and society. According to statistics, approximately 50% family members have to reduce work hours or even stop working to take care of patients with spasticity after stroke (5). The direct cost for patients with spasticity is US$84,195 during the 1st year after stroke, which were four times higher than those without spasticity (6).

Currently, there are quite a few therapeutic strategies (e.g., physiotherapy, oral spasmolytics, injections of botulinum toxin) to treat post-stroke spasticity, while the therapeutic effect of spasticity is unsatisfactory. In terms of physiotherapy, the limited effect and long-term treatment course may lead to poor compliance (7). The effect of oral spasmolytics is not long-lasting, and prolonged use of these drugs might cause multiple side effects, such as hepatotoxicity and muscle weakness (8). Repetitive injections of botulinum toxin may result in the formation of neutralizing antibodies and attenuate the treatment efficacy (9). Therefore, there is a need for an effective and safe therapy for post-stroke spasticity.

Acupuncture, as a pragmatic and safe traditional Chinese medicine (TCM) treatment (10), has been used for the rehabilitation of patients with post-stroke spasticity (11). Several SRs have explored the effectiveness of acupuncture for spasticity in stroke survivors. Notwithstanding, they showed inconsistent results (12–19). With the emergence of new randomized-controlled trials (RCTs) in recent years, we plan to conduct this SR and meta-analysis to update the evidence of the effectiveness and safety of acupuncture for post-stroke spasticity.

## Methods

The protocol of this SR has been registered on PROSPERO https://www.crd.york.ac.uk/PROSPERO/display_record.php?RecordID=129779 (registration ID: CRD42019129779) and published in advance (20). We conducted this study strictly in compliance with A Measurement Tool to Assess Systematic Reviews (AMSTAR 2.0) (21) and reported following the Preferred Reporting Items for Systematic reviews and Meta-Analysis 2020 (PRISMA) statement (22). The completed PRISMA checklist is shown in Supplementary File 1.

### Literature search

We performed a literature search in the following databases from their inception to 6 June 2022: PubMed, Embase, the Cochrane Library, Web of Science, Epistemonikos Database, Chinese Biomedical Database, Chinese National Knowledge Infrastructure, Chinese Science and Technology Periodical Database, and Wangfang Database. Comprehensive search strategies applied to the above databases were developed by a professional library staff (DLZ), which used logical operators to link subject terms and free words together. The detailed search strategies of all databases are shown in Supplementary File 2. We also searched the Chinese Clinical Trial Registry and ClinicalTrials.gov to identify possible eligible trials. Potential articles were hand-searched from gray literature, reference lists of included studies, and relevant SRs. In addition, we also consulted the experts in this field.

### Inclusion criteria

#### Types of studies

We included RCTs published in English or Chinese that evaluated the effectiveness and safety of acupuncture for post-stroke spasticity. The included studies should specify the randomization method in detail.

#### Types of participants

Patients with post-stroke spasticity were included. The stroke was diagnosed according to the acknowledged diagnostic criteria and confirmed by magnetic resonance imaging or computed tomography. The spasticity was defined as Brunnstrom stage II–V, the Modified Ashworth Scale (MAS) graded I–IV, or Composite Spasticity Scale (CSS)/Clinical Spasticity Index (CSI) >0 (23). There were no restrictions on age, gender, race, duration of stroke, type of stroke, and position of spasticity.

#### Types of interventions

The experimental group received acupuncture as a monotherapy or adjunctive therapy. We included manual acupuncture, electroacupuncture, body needling, abdominal acupuncture, scalp acupuncture, and eye acupuncture in accordance with definition of acupuncture ^1^.

#### Types of comparisons

Patients in the control group were treated with conventional rehabilitation (CR), sham acupuncture, or Western medicine (WM). CR mainly included general supportive care, kinesiotherapy, occupational therapy, and physical factor therapy. Sham acupuncture was designed using the method of “shallow needling to non-acupoints” (24).

#### Types of outcome measures

The primary outcome was the MAS score of affected limbs. The secondary outcomes included effective rate (ER) that refer to the reduction of MAS by more than one grade, Fugl–Myer Assessment (FMA), Barthel Index (BI), CSS, CSI, integral electromyography (iEMG), root mean square (RMS), a ratio of maximum H-reflex to maximum M response (H_max_/M_max_ ratio), co-contraction rate (CCR), and acupuncture related adverse events.

### Exclusion criteria

Studies were excluded if (1) they were quasi-RCT, crossover RCT, and cluster RCT; (2) patients had no clear diagnostic criteria or suffered from spasticity due to other reasons, such as traumatic brain injury, tumor, or poisoning; (3) studies explored the effect of different types of acupuncture; (4) other types of acupuncture (e.g., warm-needle moxibustion, acupoint injection, floating acupuncture, cutaneous needle, dry needling, and plum-blossom needle) were used as treatment for spasticity; (5) acupuncture combined with other TCM therapy (e.g., Chinese herb, massage, moxibustion, scraping, cupping, and bloodletting) to alleviate spasticity; and (6) data were unavailable by extensive searching.

### Study selection

The retrieved records were imported into Endnote (X9). After removing duplicates, two researchers (WJT and JS) independently reviewed the titles and abstracts to eliminate irrelevant records, and then read the rest records in full text to identify eligible studies. Disagreements were settled through team discussion or consultation with the third reviewer (JL).

### Data extraction

Two reviewers (CX and YXL) independently extracted data from the included studies using a predesigned extraction form. The following information was extracted: (1) the general characteristics of included studies, (2) demographic data of patients at study level, (3) characteristics of interventions and comparators, and (4) outcome measures. After extraction, two reviewers crosschecked to ensure accuracy. For multiarm RCTs, we extracted the eligible comparisons or extracted the comparison with inferior effect size. When the study reported indicators of spasticity more than one position, the recommended formula was used to merge the mean and standard deviation of multiple positions (25, 26). During this process, any ambiguities were resolved by the third author (RJJ).

### Evaluation of reporting quality of interventions in controlled trials of acupuncture

We used Revised Standards for Reporting Interventions in Clinical Trials of Acupuncture (STRICTA) to appraise the reporting quality of interventions in controlled trials of acupuncture (27). The STRICTA consists of six items, including acupuncture rationale, details of needling, treatment regimen, co-interventions, practitioner background, and control or comparator interventions. Each study was assessed by two independent reviewers (CZJ and YYZ) using STRICTA. Discrepancies were resolved by the third reviewer (YF).

### Assessment of risk of bias

The risk of bias was evaluated using the revised Cochrane risk-of-bias tool for randomized trials (ROB 2.0) (28). This tool contains five domains, namely, randomization process, deviations from intended interventions, missing outcome data, measurement of the outcome, and selection of the reported result. Each domain was judged as “low risk of bias,” “some concerns,” or “high risk of bias.” Two trained reviewers (CZJ and YYZ) pre-assessed the five included studies with ROB 2.0. Then, the intraclass correlation coefficient (ICC) statistic was calculated to evaluate the inter-rater agreement. If consistency reached at least 80%, formal evaluations were performed. Any disagreements were arbitrated by discussion or consensus with a third reviewer (YF).

### Statistical analysis

The ICC was calculated using Statistical Package for Social Sciences 25.0 to test consistency between reviewers. According to the ICC, the consistency was defined as poor 0.0–0.2, fair 0.21–0.4, moderate 0.41–0.6, good 0.61–0.8, and very good 0.81–1.00 (29). The Review Manager software (RevMan, version 5.4) and R software (version 4.2.0) were used for data synthesis. Mean difference (MD) or standardized mean difference (SMD) with 95% confidence intervals (CIs) was measured for continuous variables, and relative risk (RR) with 95% CI was calculated for dichotomous data. We defined *p* < 0.05 as a statistically significant difference. If MAS was presented as rank variable, we transformed it into continuous data. Chi-square test and *I*^2^ statistic were conducted to assess the heterogeneity among studies. When *I*^2^ ≤ 50%, *p* > 0.1, we used fixed-effect model to pool data; otherwise, the random-effect model was used.

### Subgroup analysis

We conducted subgroup analysis based on the following factors: (1) position of spasticity (upper limbs, lower limbs); (2) frequency of treatment (once a day, twice a day, once every other day); (3) total sessions of treatment (10–30, 30–60, >60 sessions); (4) needle stimulation (manual acupuncture, electroacupuncture); and (5) follow-up time (1 month after treatment, 3 months after treatment).

### Sensitivity analysis

Sensitivity analysis was carried out to verify the robustness of the result by removing study one by one. Furthermore, we pooled data from the studies with unclear blinding of outcome assessors and explicit blinding of outcome assessors separately. We also explored the impact of risk of bias on the pooled estimate.

### Publication bias

If the number of included trials over 10, funnel plots and *Egger*'s test were applied to detect publication bias.

### Grading of recommendations assessment, development, and evaluation

We assessed the certainty of evidence by using the Grades of Recommendation, Assessment, Development, and Evaluation (GRADE) approach (30) and summarized the evidence profile using the GRADE profiler (version 3.6) software. Each outcome was evaluated from five considerations: limitations, inconsistency, indirectness, imprecision, and publication bias. Then, the certainty of evidence was rated in four grades, namely, high quality, moderate quality, low quality, or very low quality.

## Results

### Search results

**Figure 1:**
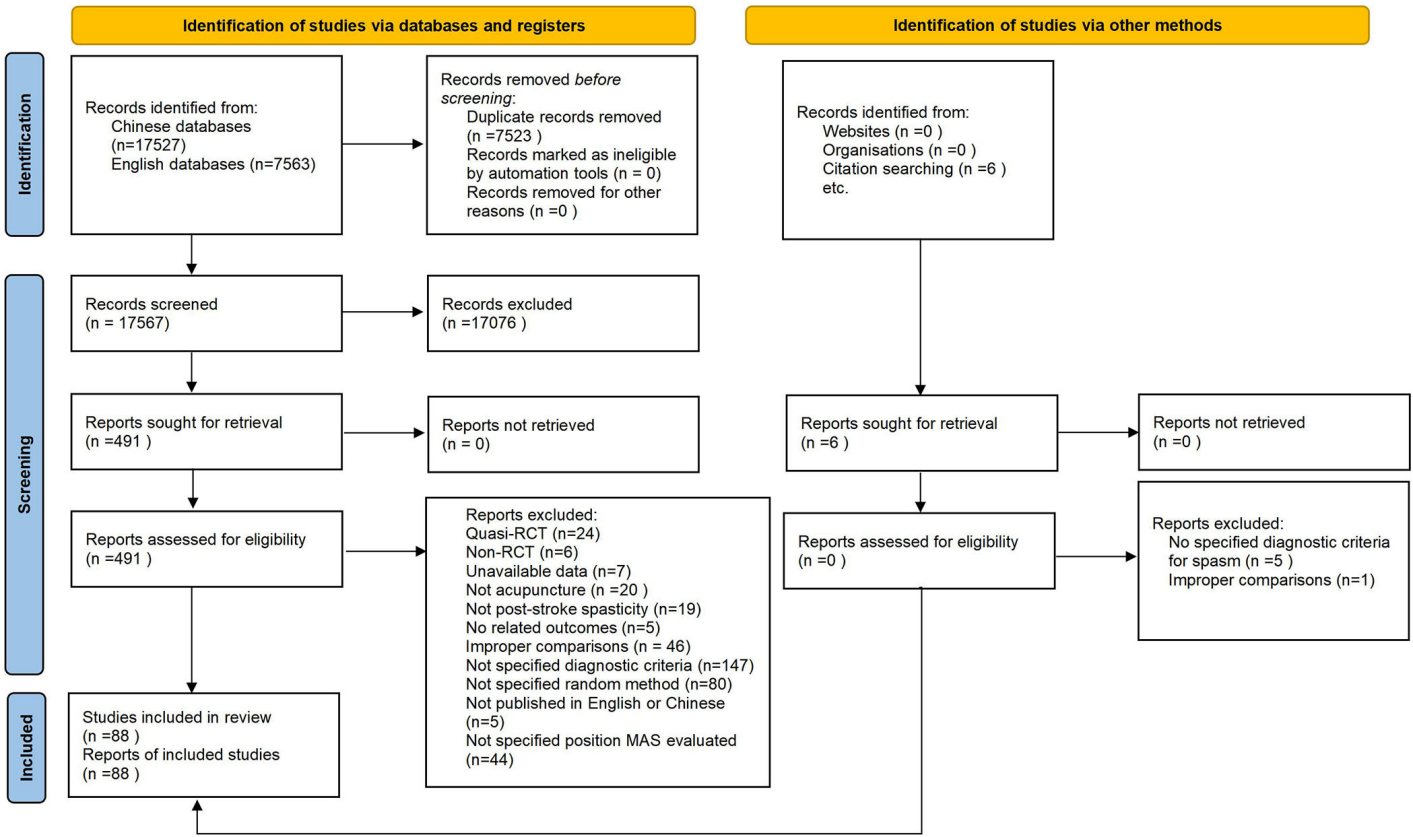
PRISMA flowchart.

A total of 25,096 records were identified, of which 7,523 duplicates were removed. By screening titles and abstracts, 17,076 irrelevant articles were eliminated. Among the remaining 497 articles, 88 studies (31–118) fulfilled the eligible criteria and were eventually included. The reasons for excluding the studies are listed in Supplementary File 3 (Supplementary Table S1). A detailed screening process is presented in Figure 1.

### Studies characteristics

The characteristics of the included studies are presented in Supplementary File 3 (Supplementary Table S2). A total of 88 studies involving 6,431 patients were identified (3,347 in the intervention group and 3,084 in the control group). The average age of the patients ranged from 50 to 78. A total of 38 studies (31, 47, 48, 52, 55, 56, 59, 60, 62, 63, 66, 67, 69, 71–76, 79, 80, 82, 83, 88–90, 92–94, 99–101, 104, 107, 110, 112–114) included patients who suffered from stroke for the first time. The duration of stroke ranged from 2 weeks to 1 year. In addition, 19 studies (35, 40, 41, 44, 45, 47, 51, 52, 61, 65, 67, 68, 70, 77, 85, 100, 108, 109, 116) recruited patients with cerebral ischemia and 1 study (84) focused on cerebral hemorrhage; the rest studies included patients with ischemic stroke and hemorrhagic stroke. A total of 41 studies (33, 36, 38, 39, 41–43, 45, 49, 53–57, 59, 61, 65–67, 70–73, 78, 80, 82, 87, 88, 90, 96, 97, 100–102, 104–106, 108, 110, 116) observed spasticity of upper limbs, 25 studies (31, 32, 44, 47, 51, 62–64, 68, 77, 79, 81, 83, 84, 86, 89, 91, 93, 94, 98, 99, 107, 111–113) focused on lower limbs, and 22 studies (34, 35, 37, 40, 46, 48, 50, 52, 58, 60, 69, 74–76, 85, 95, 103, 109, 114, 115, 117, 118) reported both upper and lower limbs. The included studies involved the comparisons of acupuncture plus CR vs. CR, acupuncture vs. CR, acupuncture vs. WM and verum acupuncture vs. sham acupuncture.

### Acupuncture protocols in included trials

**Figure 2:**
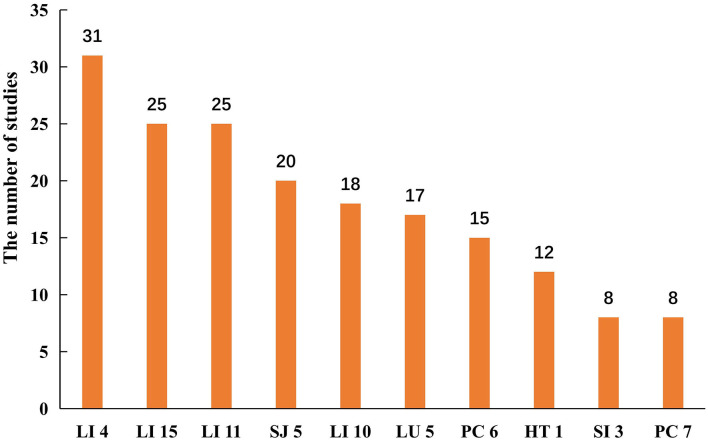
Acupoints selection on upper limbs.

**Figure 3:**
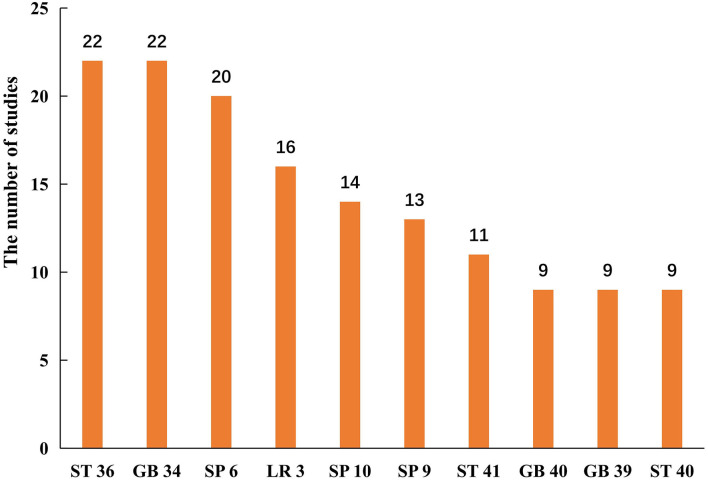
Acupoints selection on lower limbs.

A total of 61 studies (33, 35, 38, 39, 41–43, 45–48, 50, 52, 55–60, 63, 64, 66–73, 75, 78, 79, 81–90, 92, 94–96, 99, 100, 102, 103, 105–110, 112, 114–117) used manual acupuncture, and 27 studies (31, 32, 34, 36, 37, 40, 44, 49, 51, 53, 54, 61, 62, 65, 74, 76, 77, 80, 91, 93, 97, 98, 101, 104, 111, 113, 118) used electroacupuncture. All included studies described the choice of acupoints. As shown in Figure 2, the most frequent acupoints on upper limbs were *Hegu* (LI 4)*, Jianyu* (LI 15), *Quchi* (LI 11), *Waiguan* (SJ 5), and *Shousanli* (LI 10). And *Zusanli* (ST 36), *Yanglinquan* (GB 34)*, Sanyinjiao* (SP 6), *Taichong* (LR 3), and *Xuehai* (SP 10) in the lower limbs (see Figure 3). The retention time for the body acupuncture varied from 15 to 40 min. As for scalp acupuncture, the parietal median line (MS 5), parietal anterior temporal oblique line (MS 6), and parietal posterior temporal oblique line (MS 7) were commonly used. The retention time for scalp acupuncture ranged from 15 min to 6 h. Treatment frequency was once a day (31–37, 39–41, 44, 45, 47–60, 63–67, 69–81, 83–110, 112–114, 116–118) twice a day (42, 43, 61, 62, 82, 111), and once every other day (38, 68, 115). Treatment period ranged from 2 weeks (52, 109) to 6 months (68, 70). The treatment positions were mostly located on affected limbs. Only one study (54) selected acupoints on the unaffected limb (opposing acupuncture). A total of 13 studies (31, 44, 50, 55, 73, 75, 81, 85, 87, 95, 109, 111, 115) used individualized acupoint protocol according to syndrome differentiation. The remaining studies applied fixed acupoint protocol. A total of 54 studies (31, 32, 34, 35, 37, 38, 40–45, 48, 49, 52, 54, 58, 59, 61– 63, 66, 67, 71, 75, 78–80, 83–86, 88, 90, 92–98, 101–103, 105–109, 111, 113–116) emphasized *De qi*, which was a unique needling sensation and was essential for clinical efficacy (119).

### STRICTA checklist for the included studies

The STRICTA checklist is shown in Supplementary File 3 (Supplementary Table S3). Almost all studies reported the style of acupuncture, needle stimulation, acupoint selection, needle retention time, frequency, and total sessions of treatment. More than half of the studies described unilateral or bilateral of acupoints, depth of insertion, *De qi*, and thickness of acupuncture. A total of 23 studies (41, 49, 53–55, 59, 60, 66–71, 75, 79, 83, 86, 87, 89, 92, 94, 95, 100) specified the rationale of acupoint protocol and 12 studies (36, 41, 53, 55, 60, 62, 69, 71, 74, 76, 79, 87) mentioned the number of needle insertions. Except for eight studies (45, 50, 55, 58, 106, 108, 114, 116), the remaining studies described the control in detail, but none of the studies elucidated the rationale of control group. A total of 80 studies (31–44, 46–49, 51–54, 56, 57, 59–105, 107, 109–113, 115, 117, 118) reported details of co-interventions. All included studies did not specify the setting and context of treatment. Among the included studies, merely eight studies (49, 54, 68, 70, 84, 102, 113, 118) provided information about the certification of acupuncturists.

### Risk of bias assessment

**Figure 4:**
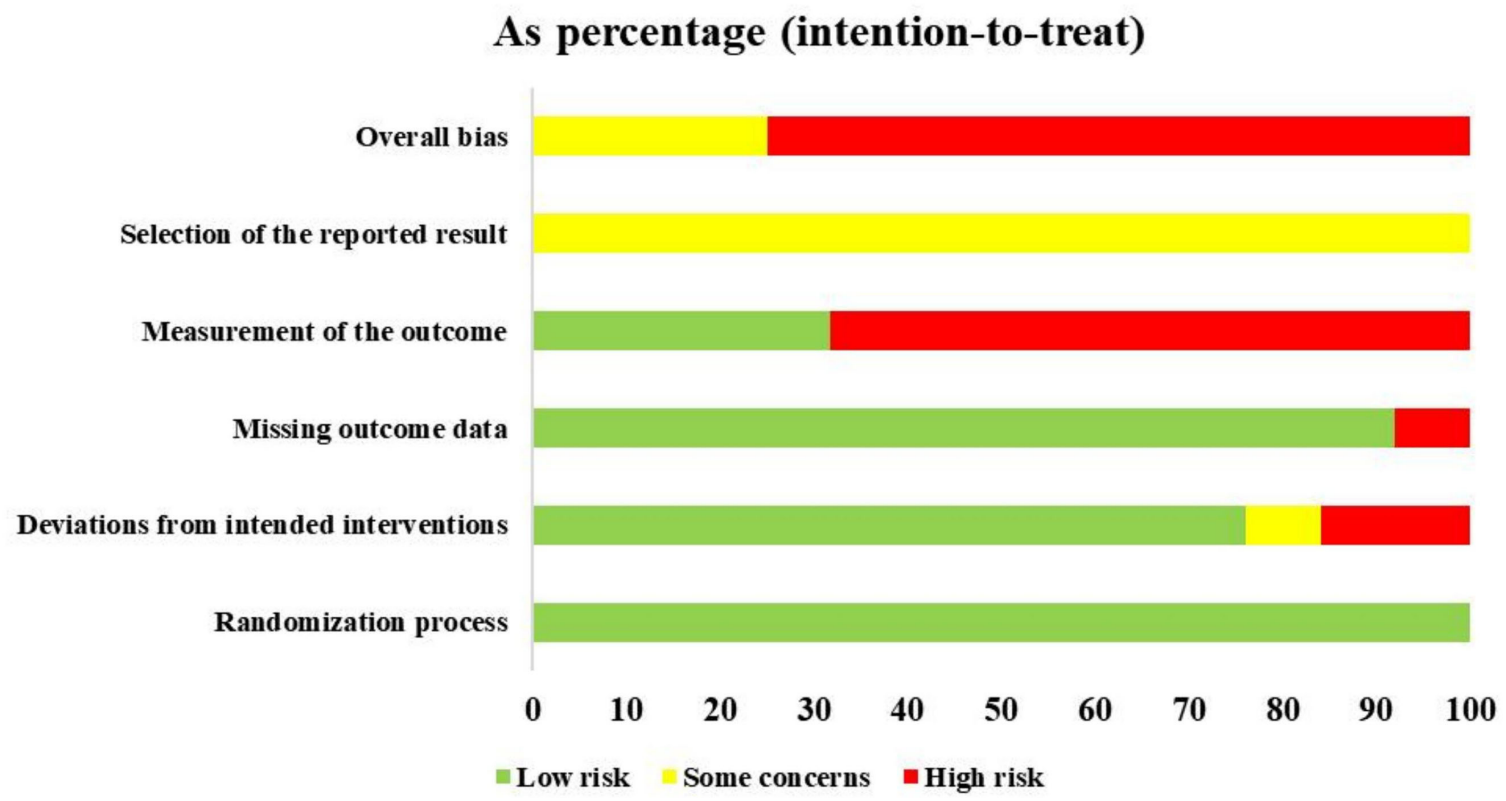
Risk of bias summary.

The ICC value between the two reviewers for ROB 2.0 assessment was 0.917, which indicates very good agreement. The summary of risk of bias is presented in Figure 4, and the graph of risk of bias is provided in Supplementary File 3 (Supplementary Figure S1). Due to no blinding of outcome assessors, deviations from intended interventions, and missing outcome data, the overall risk of bias of 66 studies (31, 34, 35, 37, 39–46, 48–53, 55, 57–59, 61–65, 67–70, 73–79, 81, 83, 85, 86, 89, 91–94, 96, 97, 99, 101–103, 105–112, 114–118) was evaluated as “high risk of bias,” and because of selective reporting results (no protocol), 22 studies (32, 33, 36, 38, 47, 54, 56, 60, 66, 71, 72, 80, 82, 84, 87, 88, 90, 95, 98, 100, 104, 113) were categorized as “some concerns.”

### Primary outcome

#### Acupuncture plus CR vs. CR

**Figure 5:**
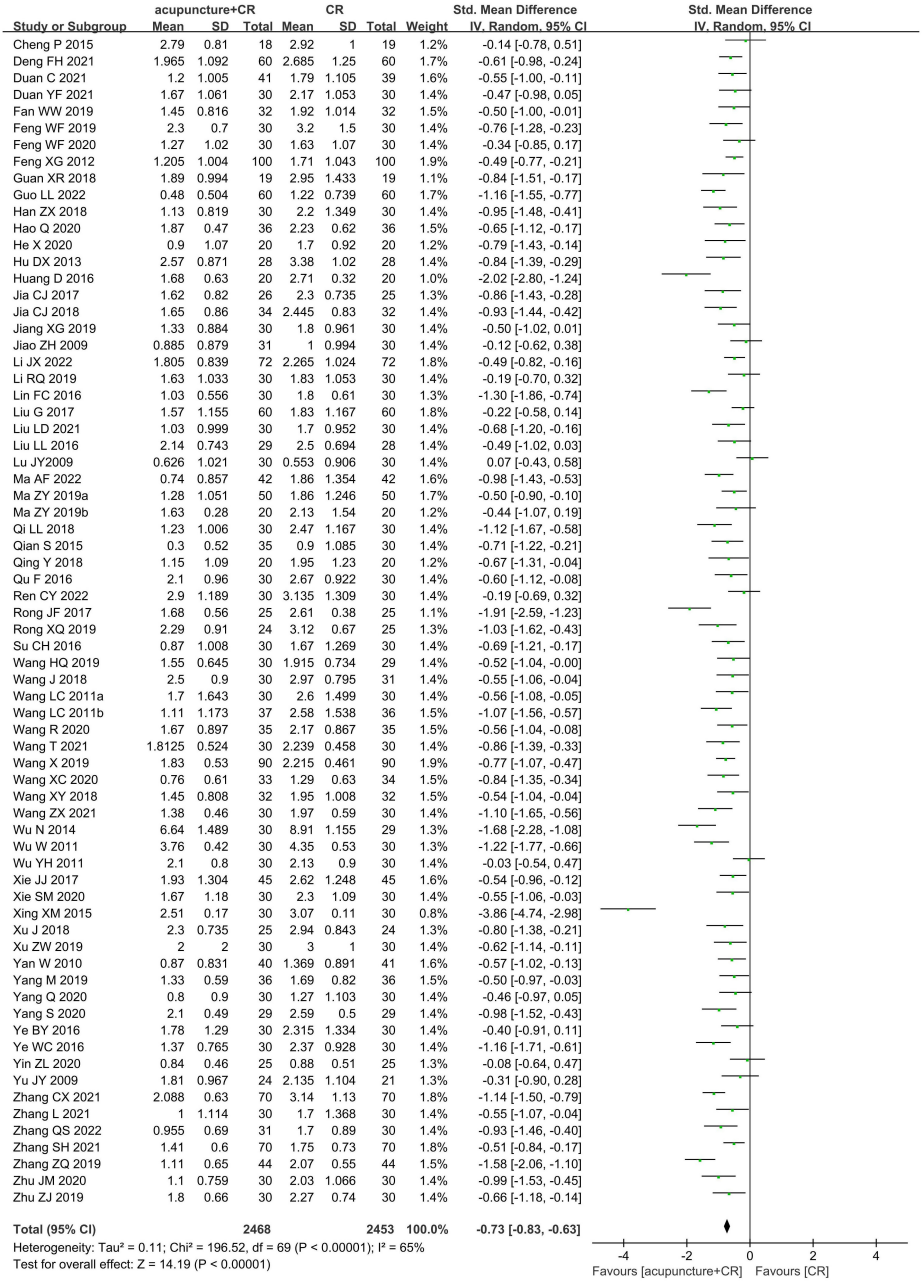
The forest plot of MAS score in comparison of acupuncture plus CR vs. CR.

**Figure 6:**
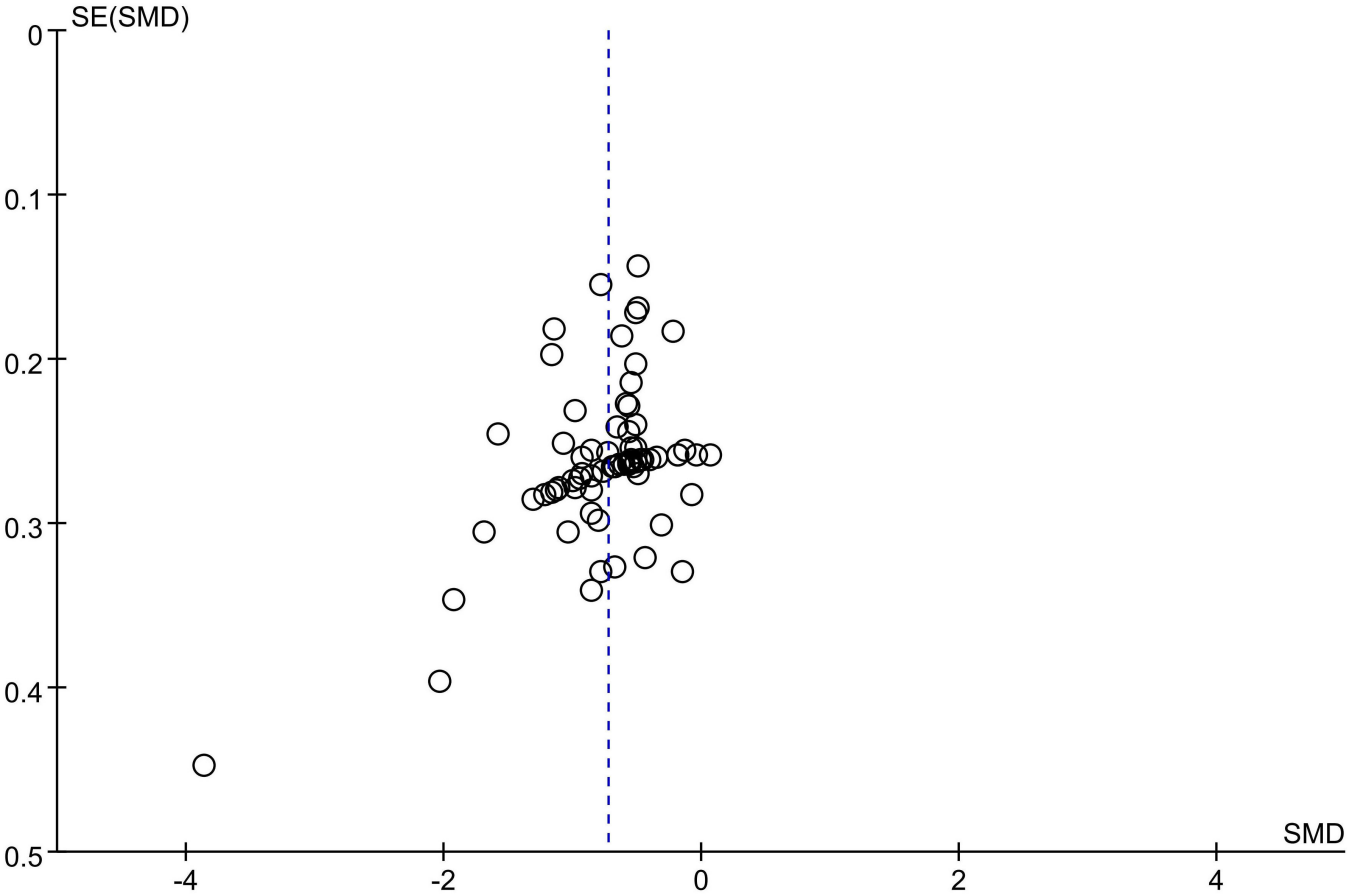
The funnel plot of MAS score in comparison of acupuncture plus CR vs. CR.

A total of 70 trials (35–37, 40, 42–51, 53–60, 62, 63, 65, 67–71, 73–77, 79–89, 91, 93–100, 102–106, 108–112, 114–118) with 4,921 participants used the MAS score to evaluate the therapeutic effect of acupuncture for post-stroke spasticity. The results of meta-analysis revealed that acupuncture plus CR was superior to the CR in decreasing MAS score (SMD = −0.73; 95% CI = −0.83 to −0.63; *p* < 0.00001; *I*^2^ = 65%) (Figure 5). The funnel plot and *Egger*'s test (*p* = 0.005) indicated that potential publication bias might exist (Figure 6).

#### Acupuncture vs. CR

**Figure 7:**
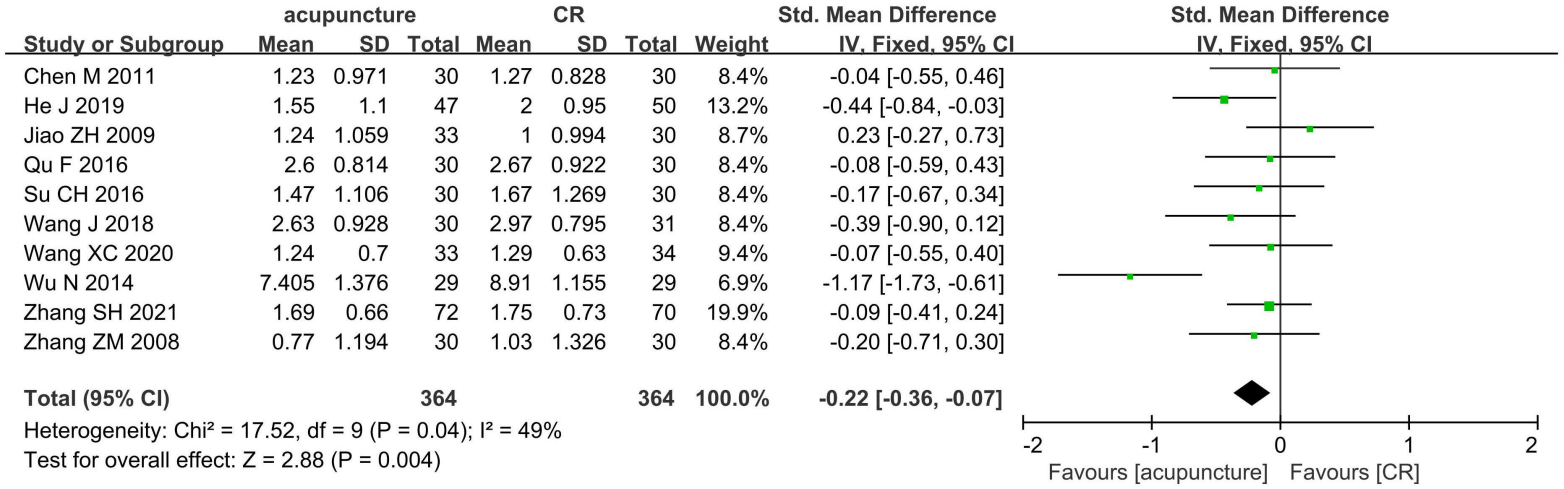
The forest plot of MAS score in comparison of acupuncture vs. CR.

**Figure 8:**
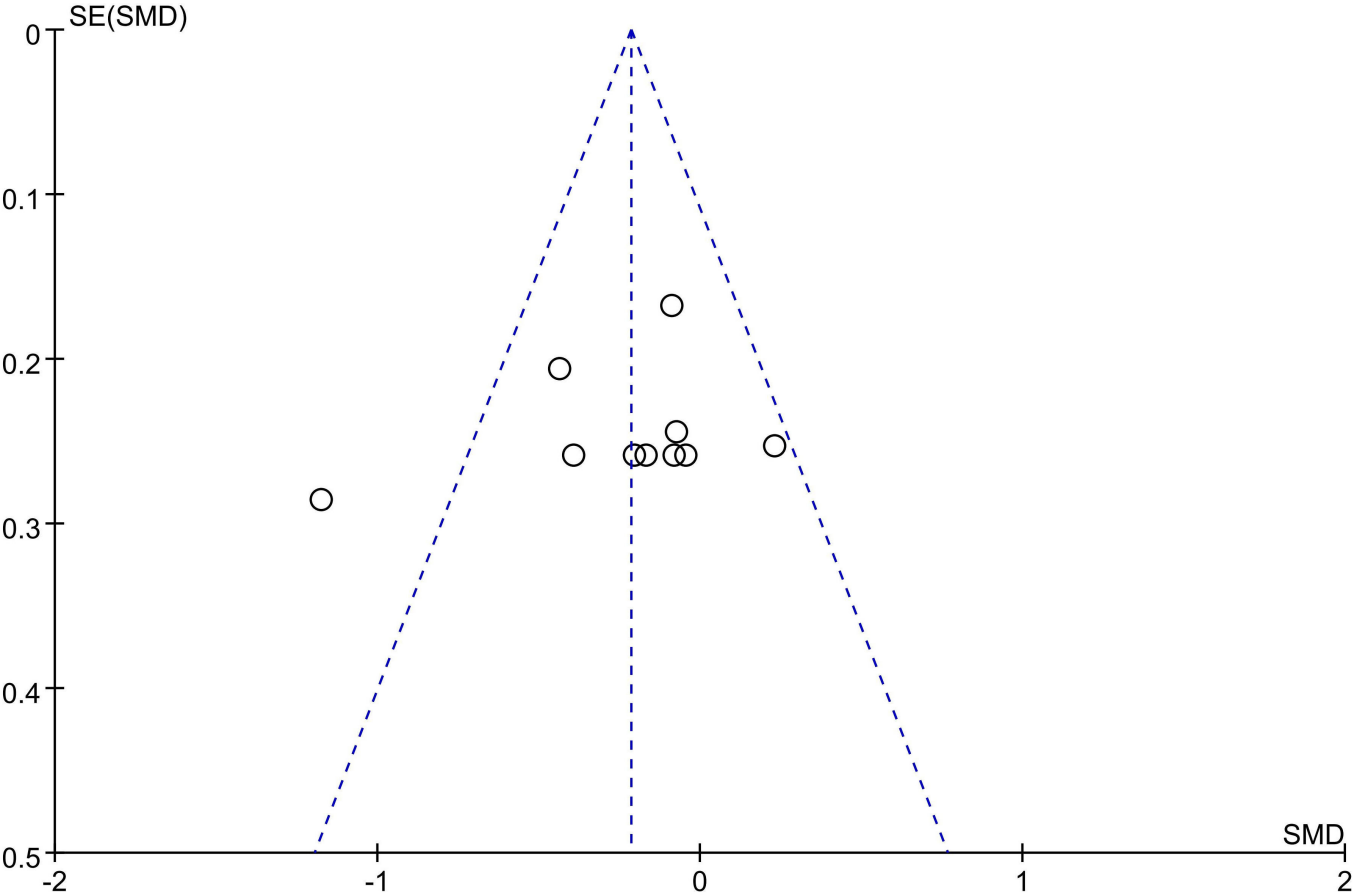
The funnel plot of MAS score in comparison of acupuncture vs. CR.

A total of 10 trials (33, 35, 41, 48, 56, 57, 73, 78, 96, 112) with 728 participants compared the effects of acupuncture with CR. The pooled data showed that acupuncture had a better effect than CR in ameliorating spasticity in patients with stroke (SMD = −0.22, 95% CI = −0.36 to −0.07; *p* = 0.004; *I*^2^ = 49%) (Figure 7). Funnel plot and *Egger*'s test (*p* = 0.486) showed no obvious publication bias (Figure 8).

#### Descriptive Analysis

Two trials (88, 90) reported that acupuncture was more effective than sham acupuncture in relieving spasticity. There was (92, 101) no significant difference between acupuncture and WM in reducing MAS score.

### Subgroup analysis

The results of subgroup analysis (acupuncture plus CR vs. CR) are summarized in Table 1. With regard to sessions of acupuncture treatment, we found that acupuncture treatments of 10–30 sessions (SMD = −0.65, 95% CI −0.76 to −0.55), 30–60 sessions (SMD = −0.79, 95% CI −1.06 to −0.52), and >60 sessions (SMD = −0.97, 95% CI −1.25 to −0.69) were superior to CR in improving post-stroke spasticity. As for acupuncture frequency, acupuncture combined with CR with once a day (SMD = −0.75, 95% CI −0.86 to −0.64) or twice a day (SMD = −0.55, 95% CI −0.85 to −0.25) reduced more MAS score than CR. However, once every other day showed no significant difference in reducing MAS score compared with the CR (SMD = −0.56, 95% CI −1.31 to 0.18). Manual acupuncture (SMD = −0.74, 95% CI −0.84 to −0.64) and electroacupuncture (SMD = −0.71, 95% CI −0.96 to −0.46) combined with CR decreased greater MAS score than CR. For different positions of spasticity, acupuncture plus CR was better than CR in improving spasticity of both upper limbs (SMD = −0.74, 95% CI −0.87 to −0.61]) and lower limbs (SMD = −0.76, 95% CI −0.94 to −0.58). One study (54) reported that acupuncture treatment had long-term effect (3 months) in ameliorating spasticity.

**Table 1:** Subgroup analyses of MAS score.

		**MAS**		
**Subgroups**	**No. of studies**	**Effect size (95% Cl)**	***P*-value**	***I* ^2^**
**Positions of spasticity**				
Upper limbs	50	−0.74[−0.87, −0.61]	<0.00001	72%
Lower limbs	39	−0.76[−0.94, −0.58]	<0.00001	81%
**Frequency of treatment**				
Once a day	62	−0.75[−0.86, −0.64]	<0.00001	66%
Twice a day	5	−0.55[−0.85, −0.25]	<0.001	46%
Once every other day	2	−0.56[−1.31, 0.18]	0.14	76%
**Total sessions of treatment, session**				
10–30	42	−0.65[−0.76, −0.55]	<0.00001	44%
30–60	17	−0.79[−1.06, −0.52]	<0.00001	82%
≥60	10	−0.97[−1.25, −0.69]	<0.00001	66%
**Needle stimulation**				
Manual acupuncture	49	−0.74[−0.84, −0.64]	<0.00001	50%
Electroacupuncture	21	−0.71[−0.96, −0.46]	<0.00001	79%
**Follow-up time**				
Immediately	70	−0.73[−0.83, −0.63]	<0.00001	65%
1 month after-treatment	2	−1.28[−1.98, −0.57]	<0.001	79%
3 months after-treatment	1	−1.17[−1.72, −0.62]	<0.00001	/

*MAS, Modified Ashworth Scale; 95% CI: 95% confidence interval*.

### Sensitivity analysis

**Figure 9:**
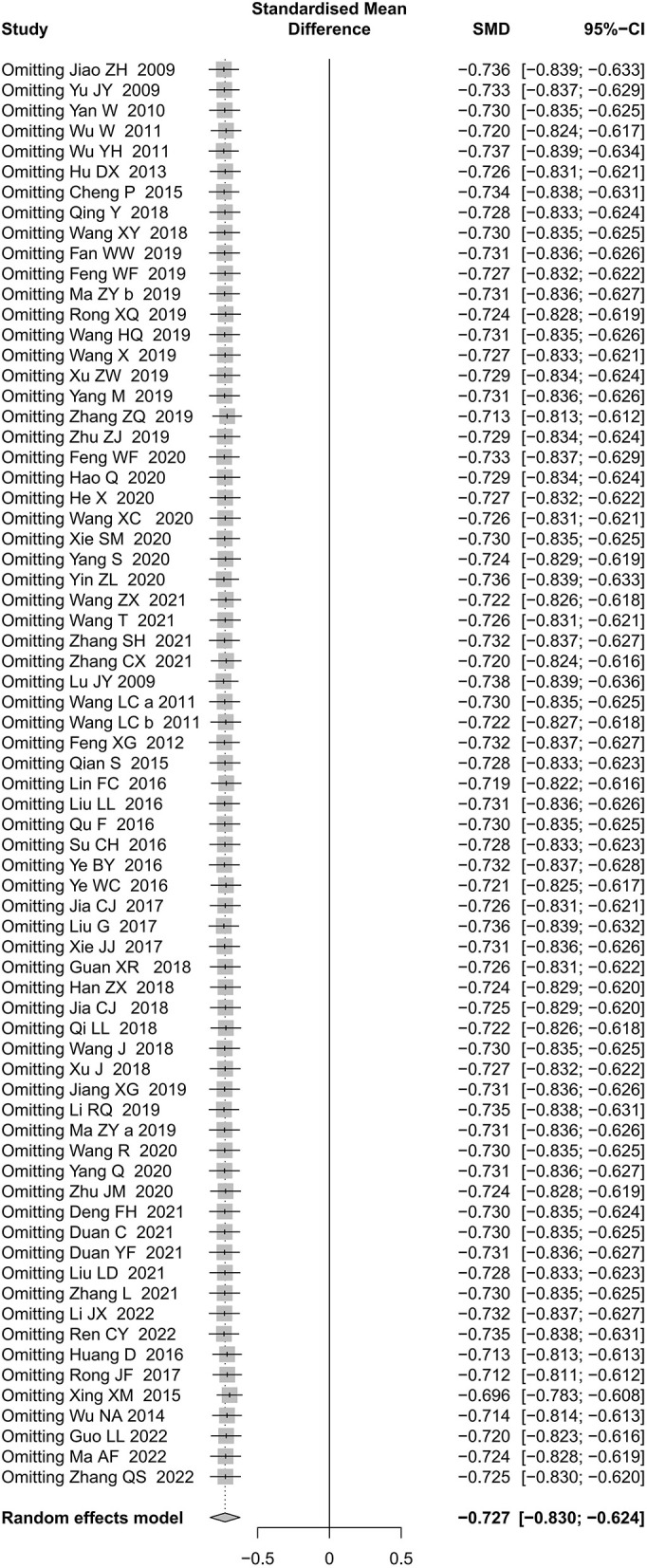
Sensitivity analysis by excluding studies one by one for MAS score (acupuncture plus CR vs. CR).

**Figure 10:**
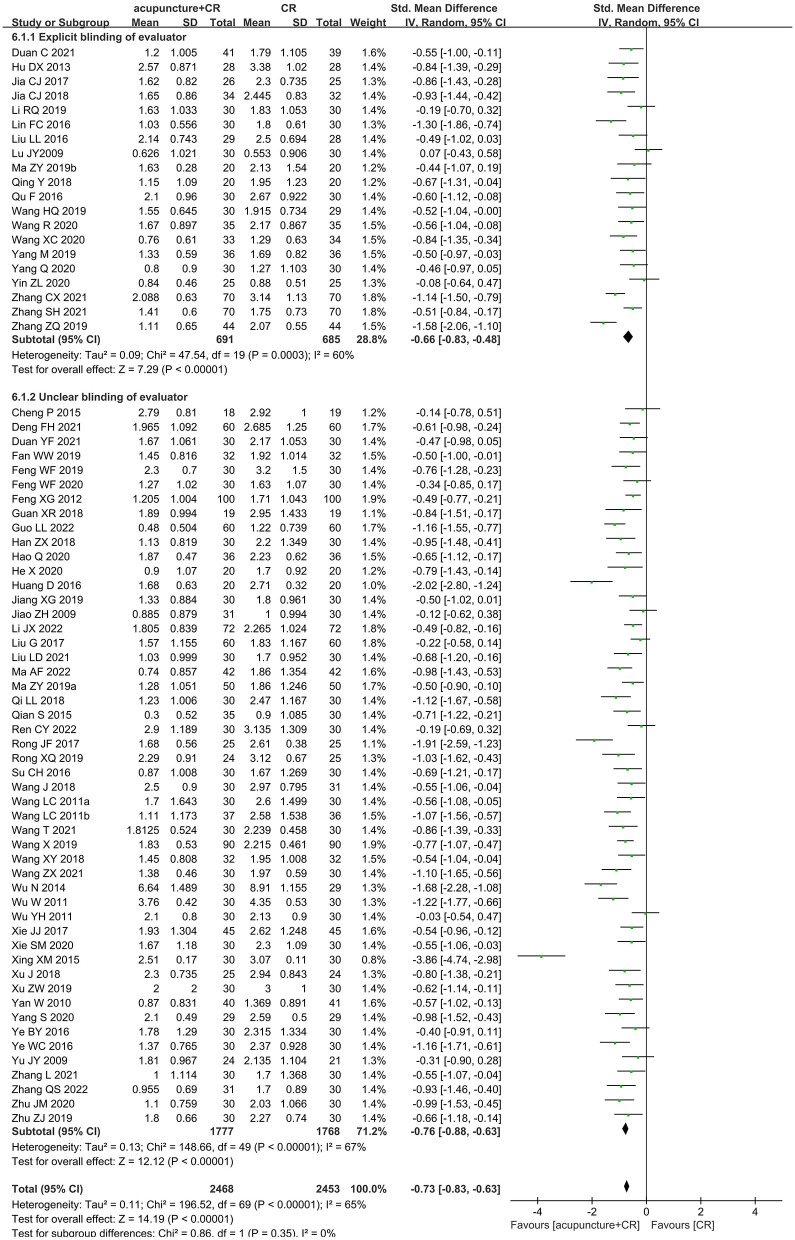
Sensitivity analysis based on blinding of outcome assessor (acupuncture plus CR vs. CR).

**Figure 11:**
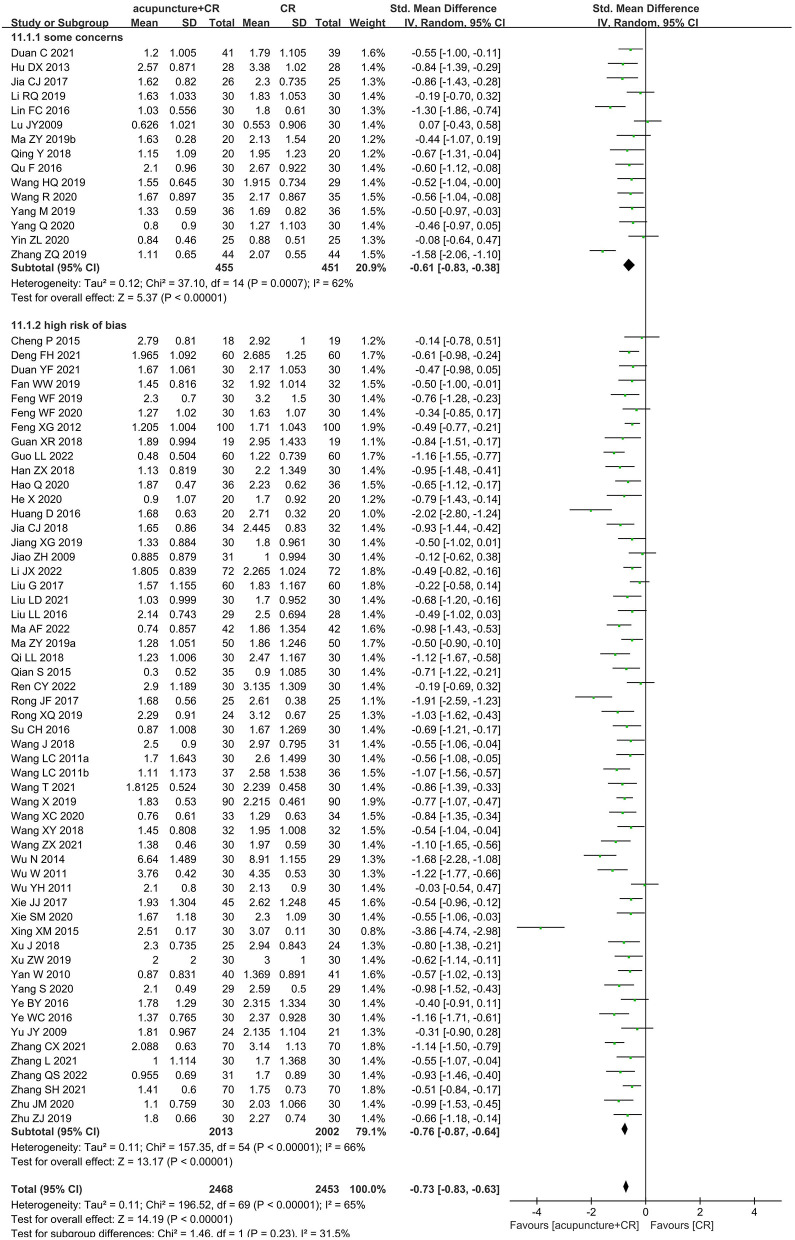
Sensitivity analysis by separately merging “high risk of bias” and “some concerns” studies (acupuncture plus CR vs. CR).

As for primary outcome in the comparison of acupuncture plus CR vs. CR, we used three methods to verify the robustness of the result. By excluding studies one by one, we found that the pooled effect size of MAS score was stable (Figure 9). By synthesizing the data from studies with unclear and explicit blinding of outcome evaluators respectively, the results demonstrated that acupuncture plus CR was superior to CR in relieving post-stroke spasticity (Figure 10). Moreover, the result was stable *via* merging studies with “some concerns” and “high risk of bias” separately (Figure 11).

**Figure 12:**
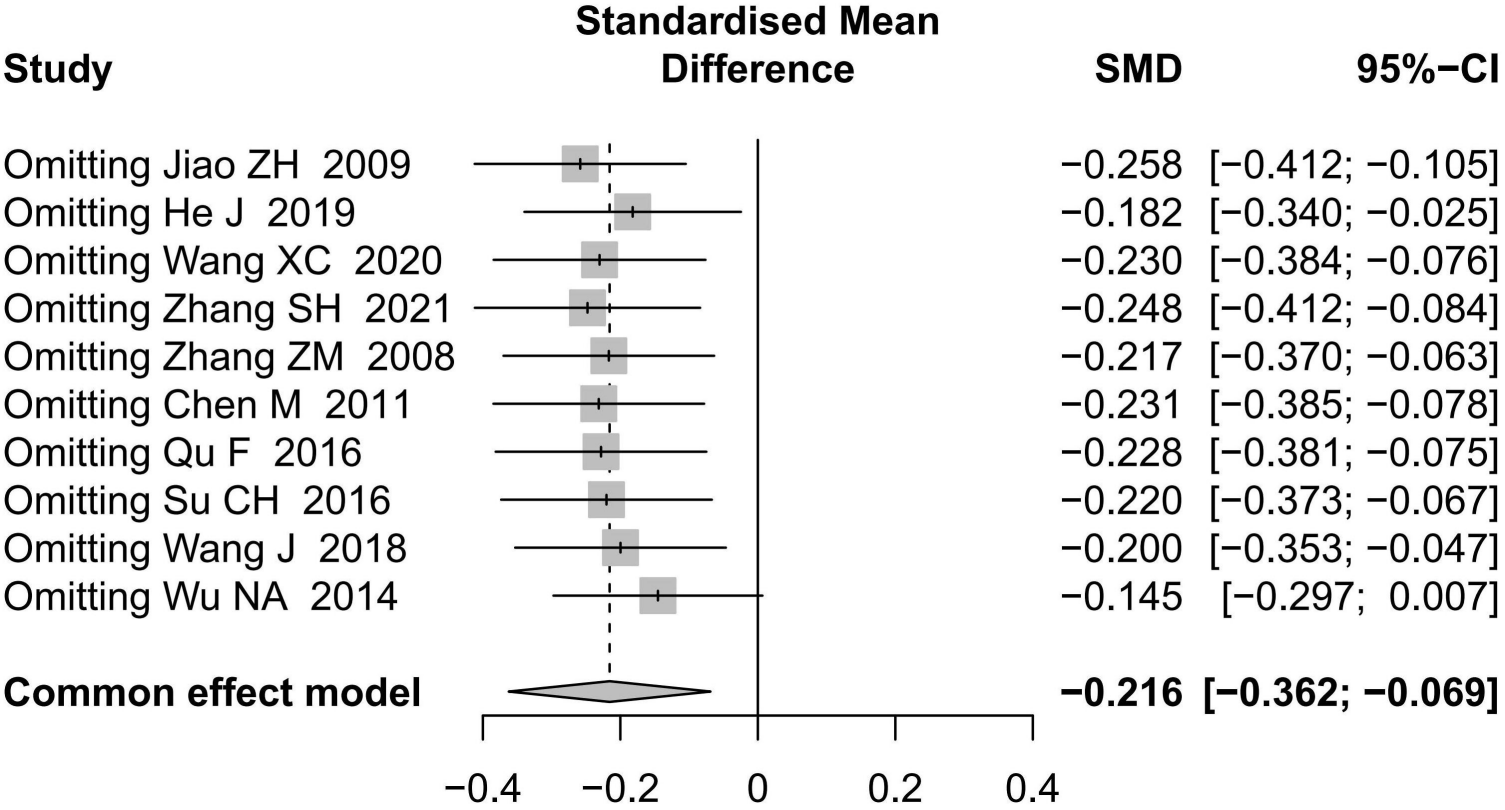
Sensitivity analysis by excluding studies one by one for MAS score (acupuncture vs. CR).

**Figure 13:**
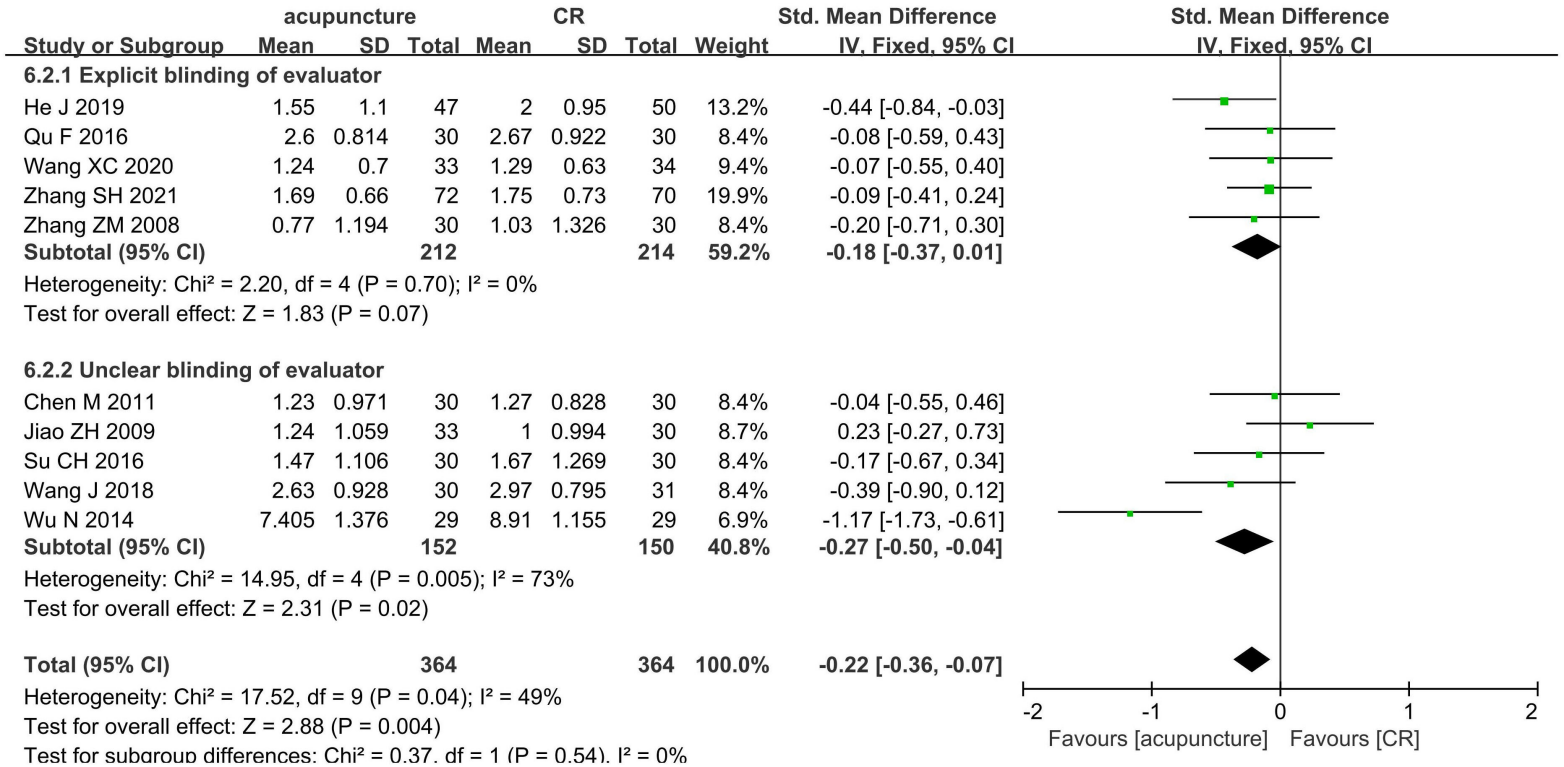
Sensitivity analysis based on blinding of outcome assessor (acupuncture vs. CR).

Regarding the MAS score in the comparison of acupuncture vs. CR, as shown in Figure 12, the result altered when excluding Wu NA 2014 (48). As shown in Figure 13, we pooled data from studies with blinding of outcome assessors and the result changed.

### Secondary outcomes

The pooled data of secondary outcomes are shown in Table 2. Meta-analysis showed that patients receiving acupuncture plus CR achieved better improvements on FMA and BI than those receiving CR alone. In addition, relevant indicators of surface electromyogram such as CCR and H_max_/M_max_ indicated that acupuncture plus CR was effective in relieving the post-stroke spasticity in patients. We also found that acupuncture was better than CR in improving BI and the upper limbs of FMA and ER.

**Table 2:** Meta-analysis of secondary outcomes.

**Outcomes**	**No. of studies**	**Effect size (95% CI)**	***P*-value**	***I* ^2^**
**Acupuncture plus CR vs. CR**				
ER-U	18	RR 1.31[1.15, 1.50]	<0.0001	78%
ER-L	10	RR 1.15[1.01, 1.32]	<0.05	67%
FMA-U	36	MD 5.56[4.42, 6.71]	<0.00001	89%
FMA-L	23	MD 3.68[2.72, 4.65]	<0.00001	86%
BI	50	MD 8.61[6.76, 10.45]	<0.00001	90%
iEMG	6	SMD 1.49[−0.05, 3.02]	0.06	97%
CCR	3	SMD −2.42[−4.69, −0.15]	<0.05	97%
RMS	5	SMD 0.02[−1.31, 1.35]	0.97	97%
CSS	3	MD −0.15[−1.47, 1.16]	0.82	77%
CSI	10	MD −1.59[−2.17, −1.01]	<0.00001	92%
H_max_/M_max_	3	SMD −0.75[−1.01, −0.49]	<0.00001	9%
**Acupuncture vs. CR**				
**ER-U**	5	RR 1.08[0.97, 1.21]	0.16	0%
CSI	3	MD −0.97[−2.23, 0.3]	0.13	79%
FMA-U	9	MD 2.87[0.46, 5.28]	<0.05	84%
FMA-L	4	MD 0.14[−0.92, 1.19]	0.8	0%
BI	9	MD 4.27[0.67, 7.88]	<0.05	69%

*No. of studies, number of studies; MD, mean difference; SMD, standardized mean difference; RR, relative risk; CR, conventional rehabilitation; 95% CI: 95% confidence interval; ER-U, effective rate of upper limb; ER-L, effective rate of lower limb; FMA-U: Fugl–Myer Assessment of upper limb; FMA-L, Fugl–Myer Assessment of lower limb; BI, Barthel Index; iEMG, integral electromyography; CCR, co-contraction rate; RMS: root mean square; CSS, composite spasticity scale; CSI, clinical spasticity index; H_max_/M_max_: ratio of maximum H reflex to maximum M response*.

### Adverse events

A total of 12 studies (33, 48, 60, 69, 75, 87, 88, 92, 94, 95, 106, 117) reported no treatment-related adverse events occurred, whereas, four studies (52, 54, 78, 118) explicitly reported adverse events, such as punctate hemorrhage (118), subcutaneous hematoma (52), subcutaneous ecchymosis (54), and needle syncope (52, 78).

### Certainty of evidence

The ER of upper limbs and MAS score in comparison of acupuncture vs. CR was rated as “moderate” certainty of evidence, while the rest outcomes were considered as “low” or “very low”. The certainty of evidence was downgraded primarily because of the high risk of bias of the included studies and inconsistency of results. A summary of findings table from the GRADE profiler is provided in Supplementary File 3 (Supplementary Figures S2, S3).

## Discussion

This SR and meta-analysis showed that acupuncture as an adjuvant therapy could effectively reduce MAS score, CCR, and H_max_/M_max_, and improve FMA and BI. Subgroup analysis demonstrated that once or twice a day acupuncture treatment and a greater total sessions of acupuncture treatment might be associated with better antispasmodic effects. Notably, 66 studies were evaluated as “high risk of bias,” 22 studies were categorized as “some concerns,” and publication bias might exist. Therefore, the above results should be treated with caution.

Our result showed that acupuncture exerted a better effect than CR in relieving post-stroke spasticity, which was consistent with the previous findings (13, 15, 16). As is known, the minimum clinically important difference (MCID) refers to the smallest change in the outcome measurements, which is considered to be clinically meaningful for patients (120). Chen et al. (120) reported the MCID of MAS with moderate clinical significance and high clinical significance were 0.48 and 0.76, respectively. The effect size of MAS (acupuncture plus CR vs. CR) in our study was 0.73, which indicated that acupuncture combined with CR had a moderate clinical effect to attenuate post-stroke spasticity.

The results of subgroup analysis demonstrated that acupuncture treatment one or two times a day was better than CR in alleviating spasticity after stroke, whereas acupuncture treatment once every other day showed no significant difference. The possible explanation was that, as the prolongation of treatment interval, the effector substance gradually attenuated and the therapeutic effect was unsustainable (121). Besides, our result suggested that more sessions of acupuncture treatment might yield greater effect to relieve spasticity. This result might be attributed to the cumulative effect of acupuncture. Notwithstanding, considering apparent heterogeneity and high risk of bias, rigorous clinical trials are required to explore the optimal protocol of acupuncture treatment for spasticity in the future.

As the spasticity worsens, movement function and daily activities of patients would be unavoidably affected. Our results showed that acupuncture plus CR enhanced motor function and activities of daily living in patients with spasticity following stroke. The MCID of FMA of upper limbs and lower limbs were 4.48 and 3.31, separately, the MCID of BI was 1.85 (122, 123). The effect size of FMA of upper limbs and lower limbs (acupuncture plus CR vs. CR) were 5.56 and 3.68, respectively, and the effect size of BI was 8.61 in our study, which demonstrated that the effects of acupuncture plus CR in improving FMA and BI were clinically meaningful.

Several problems existed in the RCTs of acupuncture for post-stroke spasticity, for example, no blinding of patients and outcome assessors, without objective outcome measures, lack of follow-up, and absence of detailed acupuncture protocol. Such problems hinder us from comprehensively and objectively evaluating the authentic efficacy of acupuncture for post-stroke spasticity. Hence, sham acupuncture should be set as comparison. Additionally, it is crucial to assess spasticity with objective indicators to obtain objective data. Future studies should also focus on the long-term effect of acupuncture for spasticity after stroke. To improve the reporting quality, researchers should report studies in accordance with the Consolidated Standards of Reporting Trials (124) and STRICTA (27).

This is the latest SR and meta-analysis that comprehensively evaluated the effectiveness and safety of acupuncture for post-stroke spasticity. The protocol of this SR and meta-analysis was registered in advance. This SR and meta-analysis was conducted strictly in accordance with AMSTAR 2.0 and reported complying with PRISMA 2020. However, some limitations should also be acknowledged. First, we used MAS score as the primary outcome measure, which is a subjective assessment scale, unclear blinding of outcome assessors and explicit blinding of outcome assessors separately, measurement bias was inevitable. Second, the published language of included RCTs in this SR and meta-analysis was limited to Chinese or English, hence, language bias might exist. Third, the overall risk of bias of the included studies was evaluated as “high risk of bias” and “some concerns.”

## Conclusion

Acupuncture could be recommended as adjuvant therapy for spasticity after stroke. However, due to the high risk of bias and heterogeneity of the included studies, the effectiveness of acupuncture for post-stroke spasticity remains to be confirmed.

## Data availability statement

The original contributions presented in the study are included in the article/Supplementary material, further inquiries can be directed to the corresponding author/s.

## Author contributions

RJ, JL, and YF designed the study. DZ designed the search strategy. WT and JS selected the studies. CXu and YL extracted the data. CJ and YZ assessed the risk of bias and reporting quality. XL, HZ, and CXi analyzed the data. CXu, CJ, and YZ wrote and drafted the manuscript. All authors approved the manuscript.

## Funding

This study was funded by the National Natural Science Foundation of China (grant no. 81674047), the Sichuan Province Science and Technology Program (grant no. 2019YFS0019), and the Sichuan Province Science and Technology Support Program (grant no. 2014SZ0154).

## Conflict of interest

The authors declare that the research was conducted in the absence of any commercial or financial relationships that could be construed as a potential conflict of interest.

## Publisher's note

All claims expressed in this article are solely those of the authors and do not necessarily represent those of their affiliated organizations, or those of the publisher, the editors and the reviewers. Any product that may be evaluated in this article, or claim that may be made by its manufacturer, is not guaranteed or endorsed by the publisher.

## Footnotes

^1^Available online at: https://www.news-medical.net/health/What-is-Acupuncture.aspx.

## References

[1] Kuo CL Hu GC. Post-stroke spasticity: a review of epidemiology, pathophysiology, and treatments. Int J Gerontol. (2018) 12:280–4. 10.1016/j.ijge.2018.05.005

[2] Young RR. Spasticity: a review. Neurology. (1994) 44:S12–20. 7970006

[3] Ward AB. A literature review of the pathophysiology and onset of post-stroke spasticity. Eur J Neurol. (2012) 19:21–7. 10.1111/j.1468-1331.2011.03448.x 21707868

[4] Kwon S Park JH Kim WS Han K Lee Y Paik NJ. Health-related quality of life and related factors in stroke survivors: data from Korea national health and nutrition examination survey (KNHANES) 2008 to 2014. PLoS One. (2018) 13:e0195713. 10.1371/journal.pone.0195713 29634768 PMC5892928

[5] Barnes M Kocer S Murie Fernandez M Balcaitiene J Fheodoroff K. An international survey of patients living with spasticity. Disabil Rehabil. (2017) 39:1428–34. 10.1080/09638288.2016.1198432 27385274

[6] Lundström E Smits A Borg J Terént A. Four-fold increase in direct costs of stroke survivors with spasticity compared with stroke survivors without spasticity: the first year after the event. Stroke. (2010) 41:319–24. 10.1161/strokeaha.109.558619 20044535

[7] Thibaut A Chatelle C Ziegler E Bruno MA Laureys S Gosseries O. Spasticity after stroke: physiology, assessment and treatment. Brain Inj. (2013) 27:1093–105. 10.3109/02699052.2013.804202 23885710

[8] Montané E Vallano A Laporte JR. Oral antispastic drugs in nonprogressive neurologic diseases: a systematic review. Neurology. (2004) 63:1357–63. 10.1212/01.wnl.0000141863.52691.44 15505149

[9] Dressler D Bigalke H. Immunological aspects of botulinum toxin therapy. Expert Rev Neurother. (2017) 17:487–94. 10.1080/14737175.2017.1262258 27852103

[10] NIH Consensus Conference. Acupuncture. Jama. (1998) 280:1518–24. 9809733

[11] Chavez LM Huang SS MacDonald I Lin JG Lee YC Chen YH. Mechanisms of acupuncture therapy in ischemic stroke rehabilitation: a literature review of basic studies. Int J Mol Sci. (2017) 18:2270. 10.3390/ijms18112270 29143805 PMC5713240

[12] Lim SM Yoo J Lee E Kim HJ Shin S Han G . Acupuncture for spasticity after stroke: a systematic review and meta-analysis of randomized controlled trials. Evid Based Complement Alternat Med. (2015) 2015:870398. 10.1155/2015/870398 25628750 PMC4299539

[13] Cai YY Zhang CS Liu SN Wen ZH Zhang AL Guo XF . Electroacupuncture for post-stroke spasticity: a systematic review and meta-analysis. Arch Phys Med Rehabil. (2017) 98:2578–89. 10.1016/j.apmr.2017.03.023 28455191

[14] Fan WJ Kuang X Hu JW Chen XW Yi W Lu LM . Acupuncture therapy for post-stroke spastic hemiplegia: a systematic review and meta-analysis of randomized controlled trials. Complement Ther Clin Pract. (2020) 40:13. 10.1016/j.ctcp.2020.101176 32347210

[15] Zhang J Zhu L Tang Q. Electroacupuncture with rehabilitation training for limb spasticity reduction in post-stroke patients: a systematic review and meta-analysis. Top Stroke Rehabil. (2020) 5:1–22. 10.1080/10749357.2020.1812938 32845210

[16] Zhu JM Zhuang R He J Ding YQ Jiang LL. Acupuncture combined with rehabilitation training in the treatment of hemiplegic upper limb spasm after stroke: a meta-analysis. Chin J Integrat Med Cardio-Cerebrovasc Dis. (2021) 19:1892–8.

[17] Park SW Yi SH Lee JA Hwang PW Yoo HC Kang KS. Acupuncture for the treatment of spasticity after stroke: a meta-analysis of randomized controlled trials. J Altern Complement Med. (2014) 20:672–82. 10.1089/acm.2014.0097 25192034 PMC4155415

[18] Yang L Tan JY Ma H Zhao H Lai J Chen JX . Warm-needle moxibustion for spasticity after stroke: a systematic review of randomized controlled trials. Int J Nurs Stud. (2018) 82:129–38. 10.1016/j.ijnurstu.2018.03.013 29631145

[19] Li Y. Acupuncture and Moxibustion for Spastic Paralysis After Stroke: A Systematic Review and Meta-Analysis of Randomized Controlled Trials [Master thesis]. Heilongjiang University of Chinese Medicine Haerbin, China (2017).

[20] Shi LH Guo LX Zhang HL Li YX Zhong DL Xiao QW . Acupuncture for post-stroke spasticity: a protocol of a systematic review and meta-analysis. Medicine. (2019) 98:e17124. 10.1097/md.0000000000017124 31574812 PMC6775425

[21] Shea BJ Reeves BC Wells G Thuku M Hamel C Moran J . AMSTAR 2: a critical appraisal tool for systematic reviews that include randomized or non-randomized studies of healthcare interventions, or both. Bmj. (2017) 358:j4008. 10.1136/bmj.j4008 28935701 PMC5833365

[22] Page MJ McKenzie JE Bossuyt PM Boutron I Hoffmann TC Mulrow CD . The PRISMA 2020 statement: an updated guideline for reporting systematic reviews. Bmj. (2021) 372:n71. 10.1136/bmj.n71 33782057 PMC8005924

[23] Balci BP. Spasticity measurement. Noro Psikiyatr Ars. (2018) 55:S49–53. 10.29399/npa.23339 30692856 PMC6278623

[24] Birch S Lee MS Kim TH Alraek T. Historical perspectives on using sham acupuncture in acupuncture clinical trials. Integr Med Res. (2022) 11:100725. 10.1016/j.imr.2021.100725 34458094 PMC8379290

[25] Zhang B Kang J Chen XM. Methods to combine standard deviations of different subgroups in meta-analysis. Chin J Evid Based Med. (2016) 16:851–4. 10.7507/1672-2531.2016013026611822

[26] Higgins JPT Thomas J Chandler J Cumpston M Li T Page MJ . Cochrane handbook for systematic reviews of interventions version 6.3 (2022). Available online at: www.training.cochrane.org/handbook

[27] MacPherson H Altman DG Hammerschlag R Youping L Taixiang W White A . Revised standards for reporting interventions in clinical trials of acupuncture (STRICTA): extending the CONSORT statement. J Altern Complement Med. (2010) 16:St1–14. 10.1089/acm.2010.1610 20954957

[28] Sterne JAC Savović J Page MJ Elbers RG Blencowe NS Boutron I . RoB 2: a revised tool for assessing risk of bias in randomized trials. Bmj. (2019) 366:l4898. 10.1136/bmj.l4898 31462531

[29] Ludbrook J. Practical statistics for medical research. Aust NZ J Surg. (1991) 61:12. 10.1111/j.1445-2197.1991.tb00019.x

[30] Atkins D Best D Briss PA Eccles M Falck-Ytter Y Flottorp S . Grading quality of evidence and strength of recommendations. Bmj. (2004) 328:1490. 10.1136/bmj.328.7454.1490 15205295 PMC428525

[31] Shi LT. Clinical Study of Electroacupuncture Combined With Exercise Therapy for Lower Limb Spasm After Stroke [Master thesis]. Heilongjiang: Academy of Traditional Chinese Medicine Haerbin (2004).

[32] He J. Surface Electromyography Observe of Different Frequent Electric Acupuncture [Master thesis]. Guangzhou University of Chinese Medicine Guangzhou, China (2008).

[33] Zhang ZM Feng CL Pi ZK Fan XY Chen HQ Zhang J. Observation on clinical therapeutic effect of acupuncture on upper limb spasticity in the patient of poststroke. Chin Acup Moxib. (2008) 28:257–60. 18481715

[34] Chu GX Yi XQ. Clinical research of electroacupuncture combined with rehabilitation therapy in the treatment of spastic paralysis of apoplexy. J Hubei Trad Chin Med. (2009) 31:13–4. 10.3969/j.issn.1000-0704.2009.08.00625417414

[35] Jiao ZH. Regulate Effects of Yin-Yang Balancing Penetration Acupuncture on Treating Ischemic Patients With Muscle Tension A Randomized Controlled Clinical Observation [Master thesis]. Shanghai University of Chinese Medicine Shanghai, China (2009).

[36] Lu JY. The Corresponds and Central Axis Acupuncture Treatment on Muscle Spasm, Motor Function and ADL of Stroke Patients With Hemiplegia [Master thesis]. Wenzhou Medical University Wenzhou, China (2009).

[37] Yu JY. Therapeutic observation of electroacupuncture and rehabilitation therapy for post-stroke spasm. J Ext Therapy of Trad Chin Med. (2009).

[38] Ni HH. Clinical observation on the treatment of post-stroke upper limb spasticity by acupuncture plus rehabilitation. Shanghai J Acup Moxib. (2010) 29::767–9. 10.3969/j.issn.1005-0957.2010.12.767

[39] Xu YL. Jing JJ. Effect of acupuncture on F wave amplitude of median nerve of hemiplegic upper limb muscle spasm and therapeutic effect. Chin J Viral Dis. (2010) 12:44–6. 10.16505/j.2095-0136.2010.01.013

[40] Yan W. Therapeutic observation of electroacupuncture and rehabilitation therapy for muscle spasm following cerebral infarction. Chin J Phys Med Rehabil. (2010) 32:139–41. 10.3760/cma.j.issn.0254-1424.2010.02.017

[41] Chen M. The Clinical Research on Regulation of Flaccid Paralysis and Spastic Paralysis After Cerebral Infarction by Acupuncture of Regulation of Yin–Yang Balancing Penetration [Doctoral thesis]. Shanghai University of Chinese Medicine Shanghai, China (2011).

[42] Wang LC. A control study of acupuncture Neiguan combined with Bobath rehabilitation training in the treatment of whole hand muscle spasm after stroke. Chin J Infom Tradit Chin Med. (2011) 18:82. 10.3969/j.issn.1005-5304.2011.06.040

[43] Wang LC. Clinical research on acupuncture Neiguan combined with rehabilitation training in the treatment of hand muscle spasm after stroke. Chin J Integrat Med Cardio-Cerebrovasc Dis. (2011) 9:689–90. 10.3969/j.issn.1672-1349.2011.06.026

[44] Wu W. Combined acupuncture and rehabilitation training for post-stroke strephenopodia. Shanghai J Acup Moxib. (2011) 30:321–3. 10.3969/j.issn.1005-0957.2011.05.321

[45] Wu YH Zhu GQ Shao Y Zuo B Hu R Zhong XY. Observation on the efficacy of acupuncture combined with rehabilitation training on hand function recovery of patients with cerebral infarction. Proceedings of 2011 Annual Conference of Chinese Society of Acupuncture and Moxibustion. Beijing (2011). p. 45–9.

[46] Feng XG. Clinical observation of acupuncture combined with rehabilitation training in the treatment of cerebral apoplexy spasm paralysis. Contemporary Med. (2012) 18:83–4. 10.3969/j.issn.1009-4393.2012.27.05523885610

[47] Hu DX Peng H. Effect of rehabilitation training combined with scalp acupuncture on muscle tension and nerve function in patients with limb spasm after cerebral infarction. Pract Clin Med. (2013) 14:25–9. 10.3969/j.issn.1009-8194.2013.07.010

[48] Wu N. *Therapeutic study of abdominal acupuncture combined with rehabilitation training on hemiplegia and spasm of ischemic stroke* [Master thesis]. Shanxi College of Traditional Chinese Medicine Taiyuan, China. (2014).

[49] Cheng P. Clinical research on transcranial direct current stimulation and electrical acupuncture therapy for upper limb spasticity after stroke. Chin Arch Tradit Chin Med. (2015) 33:1994–7. 10.13193/j.issn.1673-7717.2015.08.061

[50] Qian S Zhang LJ Fu XP. Observation on the preventive effect of acupuncture combined with rehabilitation training on the increase of hind limb muscular tension in stroke. J Sichuan Trad Chin Med. (2015) 33:144–6.

[51] Xing XM. Acupuncture and moxibustion combined with rehabilitation treatment of 30 case of spastic hemiplegia after ischemic stroke. J Liaoning Trad Chin Med. (2015) 42:595–7. 10.13192/j.issn.1000-1719.2015.03.062

[52] Yao XH. A Clinical Study on the Treatment of Spastic Paralysis After Ischemic Stroke by a Acupuncture Method Called “Three Parts as a Whole” [Master thesis]. Gansu University of Traditional Chinese Medicine Lanzhou, China (2015).

[53] Huang D Zhong Y Yi N Zhang HF. Electroacupuncture of scalp motor area and foot motor sensory area combined with manipulative treatment for spastic hemiplegia of upper limbs after cerebral apoplexy. Pract Clin Med. (2016) 17:13–5.

[54] Lin FC. Clinical Research on Effect of Treating the Upper Limb Spasticity Caused by Stroke Using Contralateral Needling and Rehabilitation Training [Master thesis]. Chengdu University of Chinese Medicine Chengdu, China (2016).

[55] Liu LL Xu YL. Clinical study of Jingjin Aligning Needling combined with rehabilitation exercise treatmenting upper limb spasm after cerebral apoplexy. Proceedings of the 12th Annual National Conference on Integrative Medicine and Neurology. Lanzhou (2016). p. 128–33.

[56] Qu F Min Y Liu YW Huang Z. Effect of acupuncture combined with vibroacoustic therapy on upper limb spasticity post stroke. Chin J Rehabil Theory Pract. (2016) 22:927–31. 10.3969/j.issn.1006-9771.2016.08.010

[57] Su CH. Clinical study of acupuncture combined with rood therapy for post-stroke upper limb functional disorder. J Guangzhou Trad Chin Med. (2016) 33:35–8. 10.13359/j.cnki.gzxbtcm.2016.01.010

[58] Ye BY Lin Y Kang JJ. Clinical observation of twelve Jing Acupoints Acupuncture and rehabilitation on muscle tension increase after cerebral apoplexy. Nei Mongol J Trad Chin Med. (2016) 35:71–2. 10.16040/j.cnki.cn15-1101.2016.03.083

[59] Ye WC Wang J Pei J Chen YJ. Therapeutic observation of acupuncture at Waiguan (TE5) and Zhigou (TE6) for post-stroke hand spasm. Shanghai J Acup Moxib. (2016) 35:935–8. 10.13460/j.issn.1005-0957.2016.08.0935

[60] Jia CJ Zhang HR Ni GX Zhang YA Su B Xu XL. Spasmodic hemiplegia after stroke treated with scalp acupuncture, music therapy and rehabilitation: a randomized controlled trial. Chin Acup Moxib. (2017) 37:1271–5. 10.13703/j.0255-2930.2017.12.005 29354990

[61] Li BJ Hou Q Wang XN. Observation on the effect of antagonistic acupuncture combined with modified constraint-induced movement therapy in upper limb spasticity after acute cerebral infarction. China Medical Herald. (2017) 14:97–100.

[62] Liu G Gong P Liu Y Shi S Han SW. Clinical study of needling antagonistic muscle with electro-acupuncture combined with bobath therapy in the treatment of strephenopodia after stroke. J Clin Acu Mox. (2017) 33:9–12. 10.3969/j.issn.1005-0779.2017.12.003

[63] Rong JF Huang QM Liu L Wang WN Zhu HW Shi W. Effects of acupuncture at myofascial trigger points on spastic foot drop and inversion after stroke. Chin J Rehabil Theory Pract. (2017) 23:591–4. 10.3969/j.issn.1006-9771.2017.05.022

[64] Su CH Zhang JS Tang MF. Effects of acupuncture combined with Rood therapy on the efficacy, motor function and ability of daily living in patients with ankle dysfunction after stroke. J Sichuan Trad Chin Med. (2017) 35:183–5.

[65] Xie JJ Sun Q Li JX. Effect of electroacupuncture antagonistic muscle acupoint combined with rehabilitation training on patients with upper limb spasm after stroke. Chin J Rehabil Med. (2017) 32:1417–9. 10.3969/j.issn.1001-1242.2017.12.022

[66] Deng SJ Zhu D. The effect of acupuncture du meridian combined with occupational therapy on upper limb motor function in stroke patients. China Modern Doctor. (2018) 56:120–3.

[67] Guan XR. The Clinical Observation of Scalp-Acupuncture Combined With DMS Treating Upper Extremity Muscle Tension After Stroke [Master thesis]. Xinjiang Medical University Urumqi, China. (2018).

[68] Han ZX Chen WH Zhou YX Qi LL Lu JJ Xu WJ . Effect of interactive scalp acupuncture on balance function in spastic hemiplegia after stroke. Chin J Rehabil Med. (2018) 33:948–52. 10.3969/j.issn.1001-1242.2018.08.013

[69] Jia CJ. Clinical Study on Scalp Acupuncture Combined With Music Therapy For Spastic Hemiplegia After Stroke [Doctoral thesis]. Nanjing University of Chinese Medicine Nanjing, China. (2018).

[70] Qi LL Han ZX Zhou YX Chen WH Chu LX Lu JY . Dynamic scalp acupuncture combined with PNF therapy for upper limb motor impairment in ischemic stroke spastic hemiplegia. Chin Acup Moxib. (2018) 38:234–8. 10.13703/j.0255-2930.2018.03.002 29701038

[71] Qing Y Huang DE Kang GH Liu Y Lin MN. Effect observation on repetitive transcranial magnetic stimulation at scalp acupoints on spastic hemiplegia of upper extremity after stroke. Rehabil Med. (2018) 28:21–5. 10.3724/SP.J.1329.2018.06021

[72] Qiu LF. Clinical Study of Abdominal Acupuncture Combined With Bobath Therapy in the Treatment of Spastic Paralysis of the Upper Limb After Stroke [Master thesis]. Guangxi University of Chinese Medicine Nanning, China. (2018).

[73] Wang J Bao YX Xiang YM Hao CH Hou ZP. Observation of curative effect of acupuncture of unblocking meridians and releasing spasticity plus rehabilitation on upper limb spastic paralysis after stroke. Mod J Integr Trad Chin Western Med. (2018) 27:699–702. 10.3969/j.issn.1008-8849.2018.07.005

[74] Wang XY Zhang HF. Effects of electroacupuncture stimulating projection zone of decussation of pyramid on muscular hypertonia after stroke. J Shanghai Trad Chin Med. (2018) 52:46–9. 10.16305/j.1007-1334.2018.11.013

[75] Xu J Zhang J Wei L Yang FM Han RX Liu J. Clinical observation of “relieving spasm and correcting deviation” acupuncture combined with rehabilitation training on the treatment of hemiplegia spasm. Chin J Integrat Med Cardio-Cerebrovasc Dis. (2018) 16:2782–5. 10.12102/j.issn.1672-1349.2018.19.005

[76] Fan WW Wang XY Zhang HF. Clinical study on electroacupuncture at conical cross-projection region for increased muscle tone due to stroke. J New Chin Med. (2019) 51:178–81. 10.13457/j.cnki.jncm.2019.01.047

[77] Feng WF Mai FY Zeng SJ Du SJ Dou YX. Effect observation on acupuncture combined with extracorporeal shock wave in the treatment of gastrocnemius spasm after stroke. J Yunnan Trad Chin Med. (2019) 40:60–1. 10.16254/j.cnki.53-1120/r.2019.02.024

[78] He J Yan MM. Clinical observation of hand three needles combined with temporal three needles in the treatment of stroke patients with hand dysfunction. Lishizhen Med Mat Medica Res. (2019) 3:377–80. 10.3969/j.issn.1008-0805.2019.02.038

[79] Jiang XG. The Clinical Study of Scalp Acupuncture Combined With MOTOmed Intelligent Training on Spasticity of Lower Limbs After Stroke [Master thesis]. Anhui University of Traditional Chinese Medicine Hefei, China (2019).

[80] Li RQ Liu CM Xi JM Feng HX Liu FL Feng XD. Effects of electro-acupuncture at Du meridian in stroke patients with upper-extremity spasticity and its character of sEMG. Chin J Rehabil Med. (2019) 34:1157–61. 10.3969/j.issn.1001-1242.2019.10.004

[81] Ma ZY. Effect of acupuncture plus low-frequency neuromuscular electrical stimulation on limb function in cerebral stroke patients with lower-limb hemiplegia. Shanghai J Acup Moxib. (2019) 38:964–8. 10.13460/j.issn.1005-0957.2019.09.0964

[82] Ma ZY Xu MF Yu XF. Clinical study on scalp acupuncture combined with mirror therapy for spastic paralysis of upper limbs due to stroke. J New Chin Med. (2019) 51:182–5. 10.13457/j.cnki.jncm.2019.01.048

[83] Rong XQ. Effect of Acupuncture Combine With DMS on Walking Ability of Patients With Foot Drooping After Stroke [Master thesis]. Xinjiang Medical University Urumqi, China. (2019).

[84] Wang HQ Hou M Bao CL Min L Li H. Effects of acupuncture treatment on lower limb spasticity in patients following hemorrhagic stroke: a pilot study. Eu neurol. (2019) 81:5–2. 10.1159/000499133 31013499

[85] Wang X Hu ZG Dai CH Chen XY Zhan Y. Effect of acupuncture and functional exercise on recovery of patients with cerebral apoplexy and recovery of cerebral blood flow and function. Chin Arch Tradit Chin Med. (2019) 37:2588–91. 10.13193/j.issn.1673-7717.2019.11.005

[86] Xu ZW. Observation on the Efficacy of Huangdi NeiZhen Combined With New Bobath Technique in Hemiplegic Lower Extremity Spasm After Stroke [Master thesis]. Guangxi University of Chinese Medicine Nanning, China. (2019).

[87] Yang M. Clinical Study of Convalescent Upper Extremity Spasm in Stroke Patients Treated by Head Acupuncture and Bobath [Master thesis]. Yunnan University of Chinese Medicine Kunming, China (2019).

[88] Zhang ZQ. Clinical Study on Acupuncture at “Feng Fascia Point”to Relieve Hand Spasm After Stroke [Master thesis]. Shanghai University of Chinese Medicine Shanghai, China (2019).

[89] Zhu ZJ Xiao HB Chen RQ Rao ML Chen DD Liu HS. Effects of scalp acupuncture interaction MOTOmed intelligent motor training on lower limb spasm and motor function in stroke patients with hemiplegia. J Anhui Med. (2019) 40:642–5. 10.3969/j.issn.1000-0399.2019.06.012

[90] Duan C Li ZL Xia WG Zheng CJ Zhang YP Li SC. Effect of acupuncture plus mirror therapy on upper limb functional recovery in stroke patients. Neural Injury and Functional Reconstruction. (2020) 15:155–8. 10.16780/j.cnki.sjssgncj.2020.03.009

[91] Feng WF Xian CJ Yuan J Mai FY. Clinical research on electroacupuncture combined with extracorporeal shock wave in the treatment of gastrocnemius spasm after stroke. Henan Tradit Chin Med. (2020) 40:1281–4.

[92] Guo QQ Zhao H Huang ZY Zhang Y Gou CY Wang YG. Observation on the effect of dynamic needle retention on upper arm flexor spasm after stroke. J Emerg Tradit Chin Med. (2020) 29:882–4. 10.3969/j.issn.1004-745X.2020.05.037

[93] Hao Q Hao CH Zhuang H Xia MF. Clinical observation on effect of electroacupuncture combined with rehabilitation training on post-stroke strephenopodia. J Shangdong Trad Chin Med. (2020) 39:957–60. 10.16295/j.cnki.0257-358x.2020.09.012

[94] He X. Clinical Observation of Acupuncture Combined With Whole Body Vertical Vibration in the Treatment of Triceps Spasm After Cerebral Apoplexy [Master thesis]. Hunan University of Chinese Medicine Changsha, China. (2020).

[95] Wang R. Clinical Research of Acupuncture on Antagonistic Muscles Combined With Rehabilitation Training for Treatment of Spastic Hemiplegia After Stroke [Master thesis]. Liaoning University of Traditional Chinese Medicine Shenyang, China. (2020).

[96] Wang XC Liu T Wang JH Zhang JJ. Post-stroke hand spasm treated with penetrating acupuncture combined with kinesiotherapy: a randomized controlled trial. Chin Acup Moxib. (2020) 40:21–5. 10.13703/j.0255-2930.20190106-k0003 31930894

[97] Xie SM Li JX. Clinical study on effects of electroacupuncture (EA) by reciprocal inhibition method on upper limb spasticity post-stroke. Chin Manip Rehabil Med. (2020) 11:11–6. 10.19787/j.issn.1008-1879.2020.03.004

[98] Yang Q Ni L. Rehabilitation effects of acupuncture combined with mirror feedback therapy on the rehabilitation of lower limb spasm in patients with cerebral apoplexy. World Chin Med. (2020) 15:2983–7. 10.3969/j.issn.1673-7202.2020.19.027

[99] Yang S. Clinical Application and Time-Dependent Effect Study of Eye Acupuncture and Rehabilitation Training in Spasm of Cerebral Apoplexy [Doctoral thesis]. Liaoning University of Traditional Chinese Medicine Shenyang, China (2020).

[100] Yin ZL Meng ZX Ge S Zhang MJ Huang LH. Clinical observation of dynamic scalp acupuncture combined with task-oriented mirror therapy for upper limbs function impairment in patients with hemiplegia after ischemic stroke. Chin Acup Moxib. (2020) 40:918–22. 10.13703/j.0255-2930.20190819-0001 32959583

[101] Zhang Q Qing H Song YN Gao LJ. Application effect of acupuncture and modern rehabilitation exercise therapy in the cerebral vascular disease of upper limb with spasmodic pattern. China Medical Herald. (2020) 17:152–9.

[102] Zhu JM Zhuang R He J Wang XX Wang H Zhu HY. Yin-yang balance penetrating acupuncture combined with rehabilitation training on upper limb spasticity in stroke hemiplegia. Chin Acup Moxib. (2020) 40:697–701. 10.13703/j.0255-2930.20190531-k0005 32648390

[103] Deng FH. Acupuncture combined with rehabilitation training to treat 60 cases of spastic hemiplegia after stroke. J Guangxi Univer Chin Med. (2021) 24:35–8. 10.3969/j.issn.2095-4441.2021.02.011

[104] Duan C Li ZL Xia WG Zheng CJ Zhang PY Li SC. Effect of“Nourishing Liver and Kidney” acupuncture combined with rehabilitation training on upper limb functional recovery in stroke patients. J Clin Acu Mox. (2021) 37:15–9. 10.19917/j.cnki.1005-0779.021027

[105] Duan YF Li YH Sun WJ Li Y Yan RR Chen SQ . Observation on therapeutic effect of yuan-source points and collateral-points combination with rehabilitation training on hand spasm after stroke. Mod J Integr Trad Chin Western Med. (2021) 30:497–501. 10.3969/j.issn.1008-8849.2021.05.009

[106] Liu LD. Clinical Study of Needling in Antagonistic Muscle Combined With Rehabilitation Exercise in the Treatment of Upper Limb Spasm After Stroke [Master thesis]. Changchun University of Chinese Medicine Changchun, China. (2021).

[107] Song YJ Zhang DQ Xu SB Jia J Qu YY Sun XW. Therapeutic research of extracorporeal shock wave combined with acupuncture on lower limb spasm after stroke. Neural Inj Funct. (2021) 16:414–22. 10.16780/j.cnki.sjssgncj.20201247

[108] Wan ZX Luo Q. Clinical effect of Hegu penetration acupuncture Houxi point in the treatment of increased muscle tension of whole hand after cerebral infarction and its influence on the daily activity ability of patients. Clin Res Pract. (2021) 6:125–7. 10.19347/j.cnki.2096-1413.202122038

[109] Wang T Liu K Li PF Sun PY Wu J Liu H. Clinical efficacy of acupuncture combined with rehabilitation training in treatment of ischemic stroke. Chin Arch Tradit Chin Med. (2021) 39:215–8. 10.13193/j.issn.1673-7717.2021.08.052

[110] Zhang CX Wang YL Zhang SH Li QF Liang WR Pan XH . Effects of scalp acupuncture combined with intelligent upper-limb feedback training for post-stroke upper-limb spasticity. Shanghai J Acup Moxib. (2021) 40:937–44. 10.13460/j.issn.1005-0957.2021.08.0937

[111] Zhang L Ran MH Xin GL Wang L Guan Y Guo XJ . Effect of acupuncture-rehabilitation method on muscle tension and motor function in patients with post-stroke lower limb spasm. J Clin Acu Mox. (2021) 37:34–8. 10.19917/j.cnki.1005-0779.021158

[112] Zhang SH Wang YL Zhang CX Xiao P Li QF Pan XH . Combining scalp acupuncture with feedback can relieve post-stroke spasticity and paralysis of the lower extremities. Chin J Phys Med Rehabil. (2021) 43:787–92. 10.3760/cma.j.issn.0254-1424.2021.09.004

[113] Huang H Chen J Qiu F Lin XM Lin ZK. Effect of electroacupuncture on motor function and gait in patients with post-stroke spasticity in lower limbs. Chin Acup Moxib. (2022) 42:23–7. 10.13703/j.0255-2930.20201201-k0004 35025153

[114] Li JX Zheng J. Clinical observation antispasmodic balanced acupuncture combined with rehabilitation training on spasmodic hemiplegia after stroke. Chin J Tradit Med Sci Technol. (2022) 29:119–21. Available online at: https://kns-cnki-net-443.webvpn.cams.cn/kcms/detail/detail.aspx?dbcode=CJFD&dbname=CJFDLAST2022&filename=TJYY202201052&uniplatform=NZKPT&v=-EdV-GZVRok-8bNAYYkiXdzc159JGTBatDLNo5SPcDflEpObEjNk8fa2Q2YCtnE0

[115] Ren CY. Acupuncture combined with rehabilitation training on clinical effect, CSI and MAS score of patients with limb spasm after cerebral apoplexy. Trad Chin Med Res. (2022) 35:12–5. 10.3969/j.issn.1001-6910.2022.02.04

[116] Guo LL Liu J. Effect of acupuncture in the treatment of patients with post-stroke upper limb hemiplegia and its influence on limb motor function. China Medical Herald. (2022) 19.

[117] Ma AF Xing YS. Effects of acupuncture combined with rehabilitation training on the electromyographic physiological indicators and motor function rehabilitation in patients with post-stroke plastic hemiplegia. Shanghai J Acup Moxib. (2022) 41:213–8. 10.13460/j.issn.1005-0957.2022.03.0213

[118] Zhang QS Zhang Y Ji GC Xu XH Wang YF Song BL. Clinical observation on cluster acupuncture at scalp points combined with exercise therapy in treatment of limb spasm after stroke. Chin Acup Moxib. (2022) 42:377–80. 10.13703/j.0255-2930.20210506-k0001 35403394

[119] Hui KK Nixon EE Vangel MG Liu J Marina O Napadow V . Characterization of the “deqi” response in acupuncture. BMC Complement Altern Med. (2007) 7:33. 10.1186/1472-6882-7-33 17973984 PMC2200650

[120] Chen CL Chen CY Chen HC Wu CY Lin KC Hsieh YW . Responsiveness and minimal clinically important difference of modified ashworth scale in patients with stroke. Eur J Phys Rehabil Med. (2019) 55:754–60. 10.23736/s1973-9087.19.05545-x 30868834

[121] Zong CS. Clinical significance of study on time-effect relationship of acupuncture. J Clin Acu Mox. (2008) 3:1–3.

[122] Chen R WU J Shen X. A research on the minimal clinically important differences of Chinese version of the Fugl-Meyer motor scale. J Anhui Med Univ. (2015) 50:519–22. 10.19405/j.cnki.issn1000-1492.2015.04.025

[123] Hsieh YW Wang CH Wu SC Chen PC Sheu CF Hsieh CL. Establishing the minimal clinically important difference of the Barthel Index in stroke patients. Neurorehabil Neural Repair. (2007) 21:233–8. 10.1177/1545968306294729 17351082

[124] Schulz KF Altman DG Moher D . CONSORT 2010 statement: updated guidelines for reporting parallel group randomized trials. Ann Intern Med. (2010) 152:726–32. 10.7326/0003-4819-152-11-201006010-0023 20335313

