# Supplementary material for: Effectiveness and safety of acupuncture for post-stroke spasticity: A systematic review and meta-analysis
Source: Front Neurol. 2022 Aug 17;13:942597. doi: 10.3389/fneur.2022.942597 (PMC9428153; doi:10.3389/fneur.2022.942597)
Supplement: Supplementary file 1 [file Data_Sheet_1.docx]

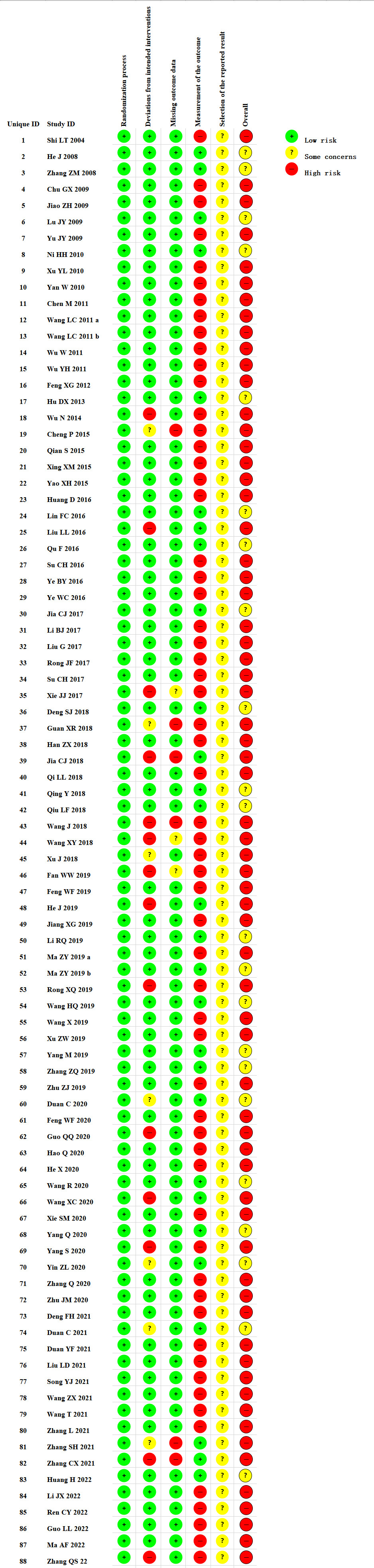


Figure S1 Risk of Bias Graph


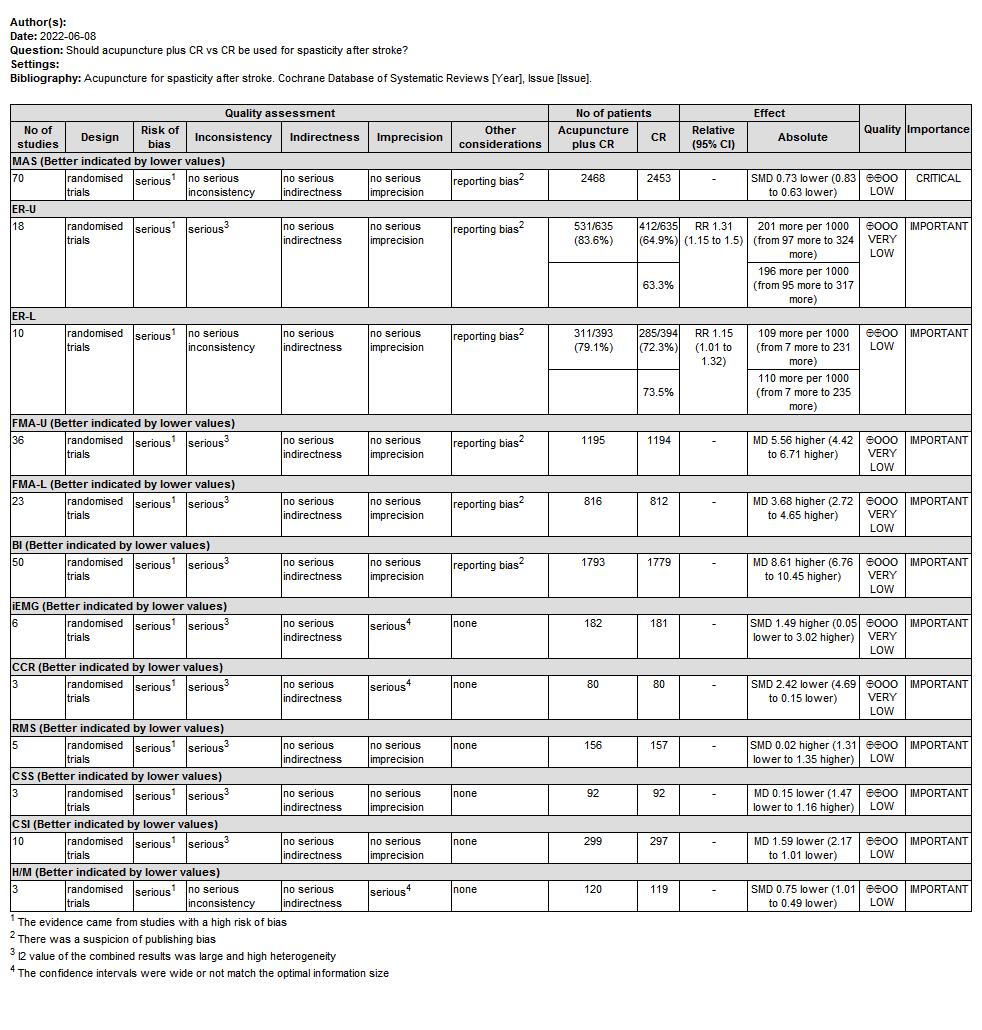


Figure S2 Results of GRADE of acupuncture plus CR vs. CR


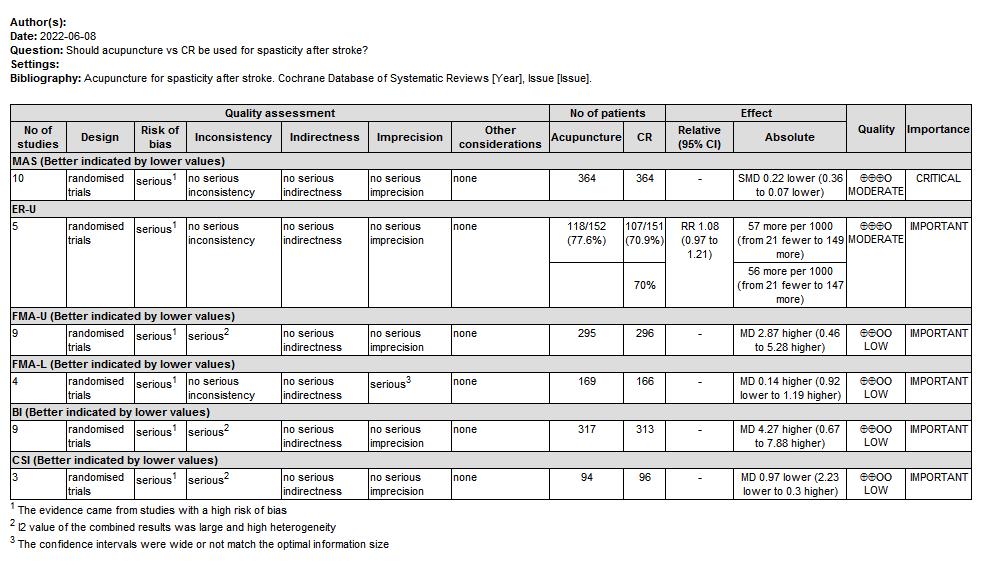


Figure S3 Results of GRADE of acupuncture vs. CR

**Table S1: A list of excluded studies and reasons for exclusion**

| **No.** | **First Author** | **Title** | **Excluded Reason** |
| --- | --- | --- | --- |
| 1 | Shukai, Han | Clinical observation on Interior-Exterior Meridians acupuncture treatment of upper limb for post stroke spasticity | Not acupuncture |
| 2 | Hongxin, Wu | Clinical Study of Acupuncture in the Treatment of Post stroke Spastic Hemiplegia | Not acupuncture |
| 3 | Xueqing, Zeng | Observation on 30 cases of spastic paralysis of upper limbs treated by acupuncture Well points combined with rehabilitation training | Not acupuncture |
| 4 | Yanping, Chen | Clinical study and application of acupuncture in the treatment of spastic hemiplegia after stroke | Not acupuncture |
| 5 | Lin, Zhang | Clinical research on acupuncture combined with rehabilitation training in the treatment of spastic paralysis of upper limbs after apoplexy | Not acupuncture |
| 6 | Hong, Li | Discuss the treatment of spastic hemiplegia after stroke by acupuncture | Not acupuncture |
| 7 | Fenfen, Chen | Clinical research on acupuncture combined with rehabilitation training in the treatment of spastic paralysis of upper limbs after apoplexy | Not acupuncture |
| 8 | Yong, Chen | Exploration on the effect of acupuncture on muscular tension after stroke | Not acupuncture |
| 9 | Pingping, Ge | Clinical study of acupuncture rehabilitation on improving limb muscle tension in stroke patients with hemiplegia | Not acupuncture |
| 10 | Jianhong, Han | Efficacy of acupuncture with rehabilitation training in treating spastic paralysis of the upper limbs after a stroke and its effect on life activities | Not acupuncture |
| 11 | Xing, Wang | Effectiveness of acupuncture and rehabilitation training in the treatment of spastic paralysis of the upper limbs after stroke | Not acupuncture |
| 12 | Yafei, Nie | Analysis of the clinical application of Chinese medicine acupuncture in the treatment of stroke hemiplegia | Not acupuncture |
| 13 | Lanlan, Yang | Analysis of the value of acupuncture in the treatment of post-stroke spastic hemiplegia | Not acupuncture |
| 14 | Yuan, Gao | Effect of acupuncture on motor function and activities of daily living in elderly patients with post-stroke spastic hemiplegia | Not acupuncture |
| 15 | Yanyan, Liu | The value of acupuncture rehabilitation care in upper limb paralysis after cerebral infarction | Not acupuncture |
| 16 | Kunpeng, Yang | Clinical Observation on Treatment of Upper Limb Spasm after Apoplexy by Compound Acupuncture | Not acupuncture |
| 17 | Yuchang, Zhao | Investigate the Clinical Effect of Acupuncture on Spastic Hemiplegia after Stroke | Not acupuncture |
| 18 | Yu, Wang | Therapeutic Effect of Acupuncture and Rehabilitation Training on Spastic Paralysis of Upper Limbs After Stroke | Not acupuncture |
| 19 | Xiaofeng, Yang | Clinical observation of acupuncture combined with rehabilitation training in the treatment of upper limb spastic paralysis after stroke | Not acupuncture |
| 20 | Liping, Xin | Effect of acupuncture combined with rehabilitation training on upper limb spastic paralysis after stroke | Not acupuncture |

**Continued**

| **No.** | **First Author** | **Title** | **Excluded Reason** |
| --- | --- | --- | --- |
| 21 | Changming, Zhong | Effects of rehabilitation on acupuncture and balance facilitation of muscular tension in early stroke hemiplegia | Not post-stroke spasticity |
| 22 | Rui, Qi | Clinical observation of early comprehensive rehabilitation of ischemic stroke and study of elbow flexor and extensor isokinetic muscle strength and surface electromyography in patients with hemiplegia | Not post-stroke spasticity |
| 23 | Wenhai, Guo | Effects of early intervention of acupuncture on spasticity after cerebral infarction | Not post-stroke spasticity |
| 24 | Tianshen, Ye | Research of Resuscitating Acupuncture Therapy and Scalp Acupuncture Intervention on Motor Function Recovery in Patients With Acute Cerebral infarction | Not post-stroke spasticity |
| 25 | Yuan, Wang | Clinical observation on effect of Yin-Yang Balancing Penetration Acupuncture Therapy in the treatment of Hemorrhage Apoplexy with muscular tension dysfunction | Not post-stroke spasticity |
| 26 | Jiamei, Chu | The Effect of Electroacupuncture Combined with Acilitation Techniques in Treating Patients with Stroke | Not post-stroke spasticity |
| 27 | Qinhui, Fu | Clinical research protocol of acupuncture for post-stroke hemiplegia | Not post-stroke spasticity |
| 28 | Xu, Zhang | The effect of a motor relearning programme combined acupuncture on muscle tension and motor function recovery after cerebral infarction | Not post-stroke spasticity |
| 29 | Wei, Chen | Clinical observation on treating hemiplegia strephenopodia by kinesiotherapy combined with the acupuncture therapy of “Qiuxu Tou Zhaohai” | Not post-stroke spasticity |
| 30 | Xiaoxin, You | Clinical evaluation of acupuncture combined with rehabilitation in the treatment of motor dysfunction in patients with ischemic stroke | Not post-stroke spasticity |
| 31 | Yang, Zhang | To investigate rehabilitation efficacy of the regulating DU meridian, Xingshen and reinforcing kidney acupuncture therapy combined with rich environment and rehabilitation training in treating ischemic stroke | Not post-stroke spasticity |
| 32 | Yongmei, Tu | Efficacy and fMRI study of acupuncture combined with stretching rehabilitation training in the treatment of post-infarction muscle spasm | Not post-stroke spasticity |
| 33 | Yinghua, Hu | Effects of early intervention of Yin-Yang Balancing Acupuncture on limb spasticity in patients with acute stroke | Not post-stroke spasticity |
| 34 | Yanqian, Fu | Clinical study on exercise acupuncture therapy on the recovery of hand dysfunction in patients with hemiplegia | Not post-stroke spasticity |
| 35 | Qi, Wang | Clinical observation on acupuncture treatment of hand dysfunction after stroke | Not post-stroke spasticity |
| 36 | Ye, Zhang | Clinical Observation on Tongjing Jiejing Acupuncture in the Treatment of Spastic Paralysis of Upper Extremity after Stroke | Not post-stroke spasticity |
| 37 | Qiuyi, Lin | Effects of electroacupuncture combined with rehabilitation training on muscle tone and motor function recovery of upper limbs during flaccid paralysis of stroke | Not post-stroke spasticity |
| 38 | Ming, Xu | Abdominal needle treatment the clinical curative effect of convalescence cerebral apoplexy hemiplegia spasm | Not post-stroke spasticity |

| **No.** | **First Author** | **Title** | **Excluded Reason** |
| --- | --- | --- | --- |
| 39 | Guo, X. | Clinical Effects of Acupuncture for Stroke Patients Recovery | Not post-stroke spasticity |
| 40 | Youqiang, Fan | Evaluation of the effect of acupuncture combined with rehabilitation training in the treatment of stroke | No related outcomes |
| 41 | Shukai ,Han | Clinical application and effect evaluation of acupuncture in the treatment of upper limb spastic paralysis after stroke | No related outcomes |
| 42 | Bo, Wang | Analysis of clinical effect of acupuncture on spastic paralysis after stroke | No related outcomes |
| 43 | Tianye, Yang | Effect of acupuncture combined with comprehensive rehabilitation therapy on neurological impairment, lower limb spasm, flaccid paralysis and cerebral blood flow in patients with early ischemic stroke | No related outcomes |
| 44 | Leem, J. | Is acupuncture effective for functional recovery in ischemic stroke? | No related outcomes |
| 45 | Yong, He | Effects of acupuncture on neurological deficit and limb motor function in stroke patients | Improper comparisons |
| 46 | Wei, Li | Clinically Therapeutic Effect and EMG Analysis on Stroke Patients with Lower -extremity Spasticity by External Counterpulsation Combined with“Xingnao -Kaiqiao”Acupuncture | Improper comparisons |
| 47 | Zhiwei, Ma | Observation on Curative Effect on Acupuncture and Rehabilitation Techniques for the Treatment of Stroke Hemiplegia | Improper comparisons |
| 48 | Xiaochuan, Rong | Analysis of the curative effect of acupuncture combined with modern rehabilitation in the treatment of Spasm on the hemiplegic side after stroke | Improper comparisons |
| 49 | Xingyuan, Wang | Effects of acupuncture on limb motor function and mobility in patients with upper limb spastic paralysis after stroke | Improper comparisons |
| 50 | Bing, Deng | Analysis of the effect of acupuncture combined with clinical rehabilitation on limb function recovery in stroke patients with hemiplegia | Improper comparisons |
| 51 | Xiaohong, Gao | Analysis of the effect of acupuncture therapy on spastic hemiplegia after stroke | Improper comparisons |
| 52 | Xudong, Gao | Analysis of the Clinical Efficacy of Acupuncture Joint Rehabilitation Therapy in the Treatment of the Sequelae of Cerebrovascular Disease in the Elderly | Improper comparisons |
| 53 | Zikang, Li | A Clinical Observation on Jin's Three-Needle Therapy Combined with Physical Exercise Reduction Therapy for Post-Stroke Spastic Hemiplegia | Improper comparisons |
| 54 | Guicai, Lu | Observation of curative effect and quality of life analysis of Needling Zhuiti region combined with suspension therapy in the treatment of post-stroke spasticity | Improper comparisons |
| 55 | Cong, Cui | Observation on the Clinical Effect of Eye Acupuncture Combined with Tong Therapy in the Treatment of Increased Muscle Tension after Stroke | Improper comparisons |
| 56 | Haiming, Wang | Clinical Observation on 48 Cases of Ischemic Stroke Hemiplegia Treated by Integrated Traditional Chinese and Western Medicine | Improper comparisons |

**Continued**

**Continued**

| **No.** | **First Author** | **Title** | **Excluded Reason** |
| --- | --- | --- | --- |
| 57 | Shaohua, Zhang | Scalp acupuncture combined with lower-limb intelligent feedback training for lower-limb motor dysfunction after stroke: a randomized controlled trial | Improper comparisons |
| 58 | Zimao, Zhang | Clinical Observation on Needle Treatment Of Upper Limb Spasticity For Post-stroke Patients | Improper comparisons |
| 59 | Feng, Xu | Effects of acupuncture combined with rehabilitation training on spasticity of post-stroke hemiplegia | Improper comparisons |
| 60 | Guoliang, Ma | Application of acupuncture combined with rehabilitation training in the treatment of hemiplegia after stroke in the elderly | Improper comparisons |
| 61 | Lin, Du | Application of acupuncture combined with rehabilitation training in the treatment of hemiplegia after stroke in the elderly | Improper comparisons |
| 62 | Yuanyuan, Han | Observation on the effect of stretch training combined with "balance yin and yang" electro-acupuncture in the treatment of strephenopodia during post stroke spasticity | Improper comparisons |
| 63 | Lixia, Hao | Clinical study on improving the muscle spasticity of upper limbs after stroke with comprehensive rehabilitation program of traditional Chinese medicine combined with modern rehabilitation training | Improper comparisons |
| 64 | Shuping, Huang | Clinical effect of electroacupuncture combined with Modified constraint-induced movement therapy on limb function recovery in stroke patients with hemiplegia | Improper comparisons |
| 65 | Libo, Sun | Application of Bobath rehabilitation technique combined with antispasmodic and rectifying acupuncture in post-stroke hemiplegia patients | Improper comparisons |
| 66 | Shuo, Yang | Clinical effect of comprehensive rehabilitation therapy in the treatment of increased upper limb muscle tone after stroke | Improper comparisons |
| 67 | Gang, Chen | Acupuncture combined with rehabilitation training for the treatment of 36 cases of upper limb spasticity after stroke | Improper comparisons |
| 68 | Shiwei, Wang | The Clinical Observation of Palm through thorn Treatment for the Chirismus after Cerebral Arterial Thrombosis | Improper comparisons |
| 69 | Xiangfa, Xu | The clinical research in treating stroke by head needle combined with acupuncture | Improper comparisons |
| 70 | Dongdi, Zhao | Clinical study on 50 cases of post-stroke spastic paralysis treated by comprehensive therapy | Improper comparisons |
| 71 | Hanliang, Ceng | Therapeutic Effects of Motor Acupuncture on Improving Walking Functions of Spastic Paralysis after Stroke | Improper comparisons |
| 72 | Shaohui, Huang | Observation on the effect of "interactive inhibition" acupuncture on 36 cases of upper limb muscle tension increased after stroke | Improper comparisons |
| 73 | Jianfang, Wang | Clinical Observation on Treatment of Spasticity after Ischemic Stroke with“Xing Nao Tong Du”Acupuncture Therapy | Improper comparisons |

**Continued**

| **No.** | **First Author** | **Title** | **Excluded Reason** |
| --- | --- | --- | --- |
| 74 | Junfeng, Xu | Influence of comprehensive rehabilitation therapy combined with “Ziwu Liuzhu” low frequency therapeutic apparatus on limb function of stroke patients with hemiplegia | Improper comparisons |
| 75 | Qin, Yuan | Eye acupuncture retaining needle combined with rehabilitation exercise clinical research for the treatment of spastic hemiplegia after stroke | Improper comparisons |
| 76 | Zhuo, Chen | The clinical observation of electric acupuncture for upper limb spasticity after stroke | Improper comparisons |
| 77 | Xiaoxiao, Lv | The effect of rehabilitation combined with acupuncture on early interventional therapy for cerebral infarction hemiplegia | Improper comparisons |
| 78 | Guobin, Sheng | Clinical observation of electro-acupuncture meridian sinew nodal points at elbow in the treatment of 30 cases of upper limb spastic paralysis after stroke | Improper comparisons |
| 79 | Li, Sun | Application of surface electromyography to evaluate the clinical efficacy of acupuncture at “Sijian” points in the treatment of hand spasm after stroke | Improper comparisons |
| 80 | Hongyang, Fan | Clinical observation on the treatment of spasmodic palsy of fingers after apoplexy with articular needling “Sifen” (EX-UE 10) | Improper comparisons |
| 81 | Xianfeng, He | Effect of Acupuncture of Myofascial Trigger Point on Spasticity of Ischemic Stroke | Improper comparisons |
| 82 | Benyuan, Li | Clinical study of acupuncture combined with modern rehabilitation in the treatment of stroke sequelae | Improper comparisons |
| 83 | Xiaofeng, Mi | Clinical observation of acupuncture combined with rehabilitation in the treatment of stroke hemiplegia | Improper comparisons |
| 84 | Xiangxiang, Guo | Acupuncture combined with rehabilitation training for the treatment of 50 cases of lower extremity spasm after stroke | Improper comparisons |
| 85 | Wenlong, Li | Therapeutic effect of Yang Xue Rou Gan acupuncture on spastic paralysis of upper limb after ischemic stroke | Improper comparisons |
| 86 | Yuxuan, Peng | Effect of Meridian Acupuncture Combined with Functional Electrical Stimulation on Lower Limb Function for Patients with Hemiplegia after Stroke | Improper comparisons |
| 87 | Yang, Tian | Clinical observation of 40 cases of post-stroke limb spasm controlled by electroacupuncture | Improper comparisons |
| 88 | Yuanzheng, Sun | Upper Limb Spastic Hemiplegia Following Cerebral Infarction Treated by Antagonistic Acupuncture Combined with Rehabilitation | Improper comparisons |
| 89 | Ailian, Hu | Clinical observation and nursing analysis of acupuncture combined with early functional exercise in the treatment of stroke patients with lower extremity spasm | Improper comparisons |
| 90 | Yunjing, Wu | Effect of electroacupuncture on limb motor function in patients with hemiplegic spasticity after stroke | Improper comparisons |
| 91 | Ye, Yuan | Observation on the Clinical Efficacy of Electroacupuncture Acupoints on Antagonistic Muscle in the Treatment of Lower Limb Dysfunction in Stroke Hemiplegia Patients | Improper comparisons |

**Continued**

| **No.** | **First Author** | **Title** | **Excluded Reason** |
| --- | --- | --- | --- |
| 92 | Rong, Du | Therapeutic Observation on Spastic Paralysis after Stroke Treated with Xingnao Kaiqiao Needling Therapy | Non-RCT |
| 93 | Weiming, Sun | Clinical observation of 30 cases of post-stroke limb spasm treated with Tiao Shen Shu Jin Acupuncture method | Non-RCT |
| 94 | Huawei, Liao | Treatment of 50 cases of post-stroke limb spasm by Zhulian’s type II inhibition needling technique at specific acupoints | Non-RCT |
| 95 | Changyin, Wang | Observation on the curative effect of acupuncture on upper limb spasm after stroke | Non-RCT |
| 96 | Xiqing, Wu | 36 cases of finger spasm after apoplexy treated by cross-penetrating needling | Non-RCT |
| 97 | Guobing, Shen | Effect of Electro-Needling Elbow Meridian Sinew K(not on Elbow Motion in Post-Stroke Spastic Period | Non-RCT |
| 98 | Gomirato, G. | Clinical results with cranial acupuncture in the treatment of spasticity | Not published in English and Chinese |
| 99 | Naeser, M. A. | Acupunture in the treatment of paralysis after stroke. I. Hemiparesis of arms and legs | Not published in English and Chinese |
| 100 | Ryu, S. H. | Effects of Electroacupuncture on the Hemiplegic Upper Extremity after Stroke | Not published in English and Chinese |
| 101 | Lee, S. W. | The Effect of Electroacupuncture on Upper-Extremity Spasticity of Stroke Patients | Not published in English and Chinese |
| 102 | Noh, J. H. | Effect of Bee-venom Acupuncture on Upper Limb Spasticity of Stroke Patients | Not published in English and Chinese |
| 103 | Shifei, Hao | Abdominal acupuncture combined with rehabilitation training in the treatment of spastic paralysis in the recovering stage of cerebral infarction | Unavailable data |
| 104 | Siqi, Jing | Clinical effect of eye acupuncture movement therapy on upper limb spasm after stroke | Unavailable data |
| 105 | Zhang, H. | Impact on rehabilitation effect of post-stroke abnormal movement pattern prevented and treated with multi-needle puncture of scalp-points | Unavailable data |
| 106 | Cai, Y. Y. | Electroacupuncture for poststroke spasticity (EAPSS): protocol for a randomised controlled trial | Unavailable data |
| 107 | Son, C. | Prospective observational study about electroacupuncture treatment for post-stroke spasticity in upper extremities: study protocol | Unavailable data |
| 108 | Zhang, Z. Q. | Acupuncture of fascia points to relieve hand spasm after stroke: a study protocol for a multicenter randomized controlled trial | Unavailable data |
| 109 | Li, J. W. | Using Surface Electromyography to Evaluate the Efficacy of Governor Vessel Electroacupuncture in Poststroke Lower Limb Spasticity: Study Protocol for a Randomized Controlled Parallel Trial | Unavailable data |

**Continued**

| **No.** | **First Author** | **Title** | **Excluded Reason** |
| --- | --- | --- | --- |
| 110 | Jinbing, Guo | Clinical Observation on Acupuncture Treating Upper Limb Dysfunction After Cerebral Infarction | No specified diagnostic criteria |
| 111 | Shaokun, Peng | Sequelae of Stroke Hemiplegia Randomized Parallel Group Study of Acupuncture Combined with Rehabilitation Therapy | No specified diagnostic criteria |
| 112 | Xiu, Zhang | Observation on the curative effect of acupuncture combined with rehabilitation training in the treatment of upper limb spasm after cerebral infarction | No specified diagnostic criteria |
| 113 | Xiaohui, Zhao | Clinical observation on the treatment of spastic paralysis of lower limbs after stroke by triple acupuncture combined with rehabilitation training | No specified diagnostic criteria |
| 114 | Zhijiang, Zhuang | Acupuncture for 54 cases of lower limb spasm after stroke | No specified diagnostic criteria |
| 115 | Guojun, Dai | Clinical Study of the Treatment for Post-stroke Spastic Hemiplegia with Electroacupuncture Combined with Rehabilitation Training | No specified diagnostic criteria |
| 116 | Ming, Liu | Clinical Evaluation of Electric Acupuncture at Antagonistic Muscle Acupoints Combined with Rehabilitation Training for the Treatment of Apoplexy Spastic Paralysis | No specified diagnostic criteria |
| 117 | Yujun, Qi | Acupuncture combined with rehabilitation training for foot drop after stroke | No specified diagnostic criteria |
| 118 | Lifeng, Qian | Clinical observation of Xingnao Kaiqiao acupuncture combined with rehabilitation training in the treatment of foot drop after stroke | No specified diagnostic criteria |
| 119 | Yanjie, Shang | Study on acupuncture combined with MOTOmed repetitive training in the treatment of post-stroke spasticity | No specified diagnostic criteria |
| 120 | Fei, Wang | Clinical observation of acupuncture combined with exercise rehabilitation training in the treatment of foot drop in the recovering stage of cerebral apoplexy | No specified diagnostic criteria |
| 121 | Xi, Wang | Curative Effect of Sidu and Hegu Acupoints in the Treatment of Finger Contracture after Stroke | No specified diagnostic criteria |
| 122 | Jingxia, Xie | Randomized Parallel Controlled Study of Acupuncture combined with Rehabilitation Treatment for Stroke Hemiplegia | No specified diagnostic criteria |
| 123 | Qiong, Yang | Effect of Chinese medicine combined with rehabilitation care on motor function recovery and quality of life in stroke paralysis patients | No specified diagnostic criteria |
| 124 | Tongtong, Zhao | Analysis on the Application of Acupuncture and Moxibustion combined with Rehabilitation Exercise in the Treatment of Elderly Stroke Hemiplegia | No specified diagnostic criteria |
| 125 | Han, Zhou | Clinical study of acupuncture combined with rehabilitation training in the treatment of spastic foot varus in stroke patients | No specified diagnostic criteria |
| 126 | Hui, Zhu | To explore the clinical effect of electro-acupuncture and rehabilitation therapy in the treatment of stroke patients with spastic hemiplegia | No specified diagnostic criteria |

**Continued**

| **No.** | **First Author** | **Title** | **Excluded Reason** |
| --- | --- | --- | --- |
| 127 | Weiwei, Dong | Clinical Efficacy and Safety of Systemic Rehabilitation Training Combined with Acupuncture Treatment for Improving the Symptoms of Foot Drop in Patients with Ischemic Stroke | No specified diagnostic criteria |
| 128 | Yi, Song | Clinical efficacy and Safety of Lushi Acupuncture in Improving Motor Dysfunction of 42 Patients with Ischemic Stroke | No specified diagnostic criteria |
| 129 | Liang, Tian | A clinical research of scalp acupuncture twisting manipulation for treatment of hemiplegia after acute ischemic stroke | No specified diagnostic criteria |
| 130 | Chunrong, Xie | A clinical study of the effect of the combination of penetration needling and modern rehabilitation on strephenopodia post stroke | No specified diagnostic criteria |
| 131 | Yun, Yang | Clinical observation acupuncture combined with modern rehabilitation therapy for upper spasm in stroke recovery | No specified diagnostic criteria |
| 132 | Yang, Yang | Effect of Electroacupuncture Combined with Bobath on Lower Limb Motor Function in Stroke Patients | No specified diagnostic criteria |
| 133 | Xuedong, Zhu | Clinical effect of acupuncture on spastic hemiplegia after stroke | No specified diagnostic criteria |
| 134 | Zonghua, Chen | Analysis of the effect of electroacupuncture combined with baclofen in the control of stroke spasticity | No specified diagnostic criteria |
| 135 | Hui, Geng | Effective observation on treating intensified muscular tension after stroke by antagonistic muscle electro-acupuncture plus rehabilitation therapy | No specified diagnostic criteria |
| 136 | Zhixia, Guo | Observation on the clinical effect of acupuncture combined with rehabilitation in the treatment of stroke hemiplegia | No specified diagnostic criteria |
| 137 | Guoping, Hong | Observation on the curative effect of acupuncture combined with rehabilitation training in the treatment of post-stroke hemiplegia | No specified diagnostic criteria |
| 138 | Yu, Liu | Effects of acupuncture on limb motor function, spastic symptoms and BI score in elderly patients with post-stroke spastic hemiplegia | No specified diagnostic criteria |
| 139 | Mengyao, Wan | The clinical observation of acupuncture combined with kinesio taping treating the shoulder-hand syndrome after stroke | No specified diagnostic criteria |
| 140 | Shaowei, Wang | Efficacy of acupuncture combined with stretching rehabilitation training in the treatment of muscle spasm after cerebral infarction | No specified diagnostic criteria |
| 141 | Yilin, Weng | Electromyographic biofeedback assessment of wrist dorsiflexion dysfunction rehabilitation by electroacupuncture after stroke | No specified diagnostic criteria |
| 142 | Rongzhen, Xu | Observational study on the effect of electro-acupuncture combined with rehabilitation therapy on post-stroke spasticity | No specified diagnostic criteria |
| 143 | Yang, Yang | Clinical effect of electronic biofeedback combined with acupuncture in restoring upper limb functional muscle strength of hemiplegic stroke patients | No specified diagnostic criteria |
| 144 | Chaobing, Zhang | Clinical observation of electromyography biofeedback combined with acupuncture in the treatment of hemiplegic foot drop after stroke | No specified diagnostic criteria |

**Continued**

| **No.** | **First Author** | **Title** | **Excluded Reason** |
| --- | --- | --- | --- |
| 145 | Liao, Zhang | Effect of TCM Xingnao Kaiqiao Acupuncture on Lower Limb Dysfunction after Stroke | No specified diagnostic criteria |
| 146 | Yisheng, Cai | Observation on the effect of acupuncture combined with rehabilitation training in the treatment of upper limb spastic paralysis after stroke | No specified diagnostic criteria |
| 147 | Yixia, Du | Clinical observation of acupuncture combined with rehabilitation training in the treatment of spastic foot varus in stroke patients | No specified diagnostic criteria |
| 148 | Xiaosheng, He | Clinical effect of electroacupuncture combined with rehabilitation training in the treatment of post-stroke spastic hemiplegia and its effect on the quality of life of patients | No specified diagnostic criteria |
| 149 | Danfeng, Liu | Clinical effect of acupuncture combined with rehabilitation training in the treatment of stroke patients with hemiplegic spasticity | No specified diagnostic criteria |
| 150 | Hongwei, Qu | Clinical Study on Acupuncture Combined with Rehabilitation Training for Spastic Hemiplegia After Stroke | No specified diagnostic criteria |
| 151 | Zhiyuan, Xi | Observation on the clinical curative effect of Dong's special acupoints combined with rehabilitation training in the treatment of spastic hemiplegia of lower limbs after stroke | No specified diagnostic criteria |
| 152 | Xiaodong, Xing | Cephalo-spinal Electroacupuncture Combined With Occupational Therapy for Treatment of Upper Limb Muscle Tension | No specified diagnostic criteria |
| 153 | Hua, Zheng | Acupuncture combined with rehabilitation for the treatment of 46 cases of post-stroke hemiplegia | No specified diagnostic criteria |
| 154 | Jiyang, Bai | Effect of acupuncture and moxibustion combined with rehabilitation function exercise on spasm state and nerve function defect in convalescent patients with cerebral apoplexy | No specified diagnostic criteria |
| 155 | Zhaohuizi, Feng | Effect of Antispasmodic Acupuncture Combined with Exercise Relearning Therapy on Muscle Tone and Motor Function in Patients with Cerebral Stroke | No specified diagnostic criteria |
| 156 | Ruiting, Gao | Application of “relieving spasm and correcting deviation” acupuncture method combined with “neurophysiological and developmental treatment technology” in post-stroke limb spasticity | No specified diagnostic criteria |
| 157 | Changling, Hu | Clinical effects of acupuncture plus rehabilitation training on muscle strength in patients with hemiplegia during cerebral infarction recovery period | No specified diagnostic criteria |
| 158 | Guilan, Huang | Effect of Spasm Three-needle Therapy combined with Repetitive Transcranial Magnetic Stimulation on Spasticity of Upper Extremity After Stroke based on sEMG | No specified diagnostic criteria |
| 159 | Yonghua, Li | To observe the clinical effect of comprehensive rehabilitation therapy in the treatment of increased upper limb muscle tone after stroke | No specified diagnostic criteria |
| 160 | Jingshao, Liu | Study on the functional recovery of patients with cerebral infarction by acupuncture combined with rood technique | No specified diagnostic criteria |

**Continued**

| **No.** | **First Author** | **Title** | **Excluded Reason** |
| --- | --- | --- | --- |
| 161 | Weiming, Liu | Clinical study on electroacupuncture at Zusanli and Fenglong points on foot varus in stroke patients | No specified diagnostic criteria |
| 162 | Lifei, Ma | Clinical effect of acupuncture on upper extremity dysfunction after stroke based on EEG and EMG research | No specified diagnostic criteria |
| 163 | Jinli, Zhu | Effect of Jiaji Point-based Acupuncture Therapy Combined with Early Rehabilitation Training on Stroke Patients | No specified diagnostic criteria |
| 164 | Qingqing, Liu | Effects of “Tiaoshen Jiejing” Acupuncture combined with PNF therapy on patients with upper limb spastic hemiplegia after stroke | No specified diagnostic criteria |
| 165 | Weizhe, Liu | The effects of the Miuci Juci acupuncture plus Bobath therapy on shoulder-hand syndrome after stroke | No specified diagnostic criteria |
| 166 | Fengli, Lv | Effect of Acupuncture at Points of Foot Shaoyang Meridian and Yinqiaomai Combined with Standing Center of Gravity Control Exercise on Rehabilitation of Patients with Foot Varus after Stroke | No specified diagnostic criteria |
| 167 | Changming, Zhong | Effects of rehabilitation on acupuncture and balance facilitation of muscular tension in early stroke hemiplegia | No specified diagnostic criteria |
| 168 | Jianmin, Lu | Clinical observation on needling combined with rehabilitation treatment of foot-drop for post-stroke patients | No specified diagnostic criteria |
| 169 | Yun, Qu | Rehabilitation therapy centralized on facilitation and acupuncture on upper extremities spasm after stroke | No specified diagnostic criteria |
| 170 | Wei, Yan | Acupuncture combined with rehabilitation training to relieve hemiplegia spasm after stroke | No specified diagnostic criteria |
| 171 | Tian, Shen | Clinical Observation on Acupuncture Treatment of Increased Lower Limb Muscle Tension in Patients with Stroke | No specified diagnostic criteria |
| 172 | Haihui, Zeng | Clinical study on acupuncture combined with rehabilitation training to improve muscle spasm after stroke | No specified diagnostic criteria |
| 173 | Yuying, Li | Acupuncture in the treatment of hemiplegic spasticity and functional exercise guidance for ischemic stroke | No specified diagnostic criteria |
| 174 | Tianzheng, Wang | Observation on the effect of acupuncture on upper limb spasticity after stroke | No specified diagnostic criteria |
| 175 | Qian, Xue | Effect of Electroacupuncture at Points of Yangming Meridians on Motor Function in Hemiplegic Patients | No specified diagnostic criteria |
| 176 | Yafeng, Ren | Clinical study of acupuncture combined with rehabilitation therapy in the treatment of post-stroke spasm | No specified diagnostic criteria |
| 177 | Huaan, Cai | 30 cases of post-stroke spasticity treated with “neurophysiological and developmental treatment technology”and acupuncture | No specified diagnostic criteria |
| 178 | Yanni, Chen | Observation on the effect of acupuncture on upper limb spasticity after stroke | No specified diagnostic criteria |

**Continued**

| **No.** | **First Author** | **Title** | **Excluded Reason** |
| --- | --- | --- | --- |
| 179 | Hongxing, Li | Electroacupuncture combined with rehabilitation training for foot drop after stroke: 30 cases | No specified diagnostic criteria |
| 180 | Huanhuan, Ni | Acupuncture combined with rehabilitation training for upper limb spasm after stroke: 20 cases of clinical observation | No specified diagnostic criteria |
| 181 | Juan, Yang | Study on acupuncture therapy of dredging governor vessel for regulating mentality in post ischemic stroke hemiplegic patients | No specified diagnostic criteria |
| 182 | Huimin, Zhang | Rehabilitation evaluation on post-stroke abnormal movement pattern prevented and treated with acupuncture and rehabilitation | No specified diagnostic criteria |
| 183 | Qinning, Cao | Effects of acupuncture on antagonistic muscles combined with rehabilitation therapy on spasticity of hemiplegia after acute cerebral apoplexy | No specified diagnostic criteria |
| 184 | Hongliang, Cheng | Clinical research on Tongdu Tiaoshen Acupuncture method on the treatment of post-stroke spasticity | No specified diagnostic criteria |
| 185 | Yunhua, Gao | Clinical observation of electro-acupuncture treatment for 82 cases with cerebral infarction sequela | No specified diagnostic criteria |
| 186 | Ruiying, Ge | 129 cases of spastic paralysis treated by electroacupuncture combined with modern rehabilitation techniques | No specified diagnostic criteria |
| 187 | Yihuang, Han | Evaluation of clinical efficacy of spastic gait after stroke based on footprint analysis | No specified diagnostic criteria |
| 188 | Meiyu, Jiang | Observation on the curative effect of acupuncture at Qiaomai point combined with rehabilitation in the treatment of foot varus after apoplexy | No specified diagnostic criteria |
| 189 | Tao, Zong | Therapeutic Observation on Acupuncture plus Rehabilitation for Upper-limb Spasticity after Cerebral Apoplexy | No specified diagnostic criteria |
| 190 | Na, Liu | Preliminary discussion on the significance of foot angle in evaluating the improvement of spastic gait in stroke | No specified diagnostic criteria |
| 191 | Tianzheng, Wang | Observation on the curative effect of acupuncture combined with modern rehabilitation therapy on foot drop after stroke | No specified diagnostic criteria |
| 192 | Zhenglu, Yin | Effects of Acupuncture on Ankle Varus for Hemiplegics | No specified diagnostic criteria |
| 193 | Ming, Zhang | 33 Cases of Spastic Hemiplegia Treated by Acupuncture Combined with Rehabilitation Therapy | No specified diagnostic criteria |
| 194 | Hua, Mao | Clinical observation of 32 cases of hemiplegia spasticity after cerebral hemorrhage treated by acupuncture antagonist muscle therapy and Bobath technique | No specified diagnostic criteria |
| 195 | Chanjuan, Ouyang | Effects of acupuncture at Zusanli and Yanglingquan through the thorns yinlingquan on foot drop and walking ability in patients with stroke | No specified diagnostic criteria |

**Continued**

| **No.** | **First Author** | **Title** | **Excluded Reason** |
| --- | --- | --- | --- |
| 196 | Yuanzheng, Sun | Efficacy of Electro-acupuncture Combined with Rehabilitative Technology on Foot Drop after Stroke | No specified diagnostic criteria |
| 197 | Changchun, Wei | Observation on the curative effect of rehabilitation combined with acupuncture in the treatment of post-stroke hypertonia | No specified diagnostic criteria |
| 198 | Haiyan, Xu | Observation on the curative effect of acupuncture combined with rehabilitation training in the treatment of foot drop in the recovering stage of stroke | No specified diagnostic criteria |
| 199 | Dan, Yang | Therapeutic Observation of Acupuncture at the Heel Vessels plus Rehabilitation for Post-stroke Strephenopodia | No specified diagnostic criteria |
| 200 | [Jongbae Park](https://pubmed.ncbi.nlm.nih.gov/?sort=pubdate&term=Park+J&cauthor_id=16186474) | Acupuncture for subacute stroke rehabilitation: A sham-controlled, subject- and assessor-blind, randomized trial | No specified diagnostic criteria |
| 201 | Wayne, P. M. | Acupuncture for upper-extremity rehabilitation in chronic stroke: A randomized sham-controlled study | No specified diagnostic criteria |
| 202 | Huanqin, Li | Effect of "Deqi" during the Study of Needling "Wang's Jiaji" Acupoints Treating Spasticity after Stroke | No specified diagnostic criteria |
| 203 | Xiaolong, Lv | Clinical study on the treatment of spastic hemiplegia after stroke with acupuncture combined with neuromuscular electrical stimulation | No specified diagnostic criteria |
| 204 | Zhengyang, Zhang | Clinical study on the treatment of spastic hemiplegia after stroke with acupuncture combined with neuromuscular electrical stimulation | No specified diagnostic criteria |
| 205 | Liping, Chen | Observation on the curative effect of acupuncture combined with rehabilitation training in patients with upper limb spasm after stroke | No specified diagnostic criteria |
| 206 | Xiaona, Du | Clinical observation of aligned acupuncture therapy for antagonistic muscles in the treatment of post-stroke hypertonia | No specified diagnostic criteria |
| 207 | Fanghong, Lan | Clinical effect of whole meridian acupuncture combined with rehabilitation training in the treatment of convalescent hemiplegia spasticity | No specified diagnostic criteria |
| 208 | Qin,Wan | Clinical Analysis of Rehabilitation Combined with Acupuncture Treatment of Post-stroke Hypermyotonia | No specified diagnostic criteria |
| 209 | Zhanjun, Cui | Clinical study of Applying JIN's Acupuncture Method and Comprehensive Rehabilitation Exercise in the Treatment of Post Stroke Spastic Hemiplegia | No specified diagnostic criteria |
| 210 | Zanhua, Wu | Observation on the effect of acupuncture on corresponding points of antagonistic muscles combined with Bobath therapy in the treatment of hemiplegic spasticity | No specified diagnostic criteria |
| 211 | Yupeng, Ying | To explore the clinical efficacy of acupuncture combined with rehabilitation training in the treatment of upper limb spasticity after stroke | No specified diagnostic criteria |
| 212 | Lin, Ding | Effects of Acupuncture on Hand Dysfunction after Stroke: Evaluated with Hand Function Laserimager | No specified diagnostic criteria |
| 213 | Cuili, Huang | Clinical value of opposing acupuncture combined with rehabilitation therapy in 64 patients with post-stroke spastic hemiplegia | No specified diagnostic criteria |

**Continued**

| **No.** | **First Author** | **Title** | **Excluded Reason** |
| --- | --- | --- | --- |
| 214 | Sheng, Huang | Analysis of clinical efficacy of acupuncture in the treatment of spastic hemiplegia after stroke | No specified diagnostic criteria |
| 215 | Xiaoxue, Liu | Study on the effect of Jin's three-needle therapy combined with rehabilitation training in the treatment of spastic hemiplegia caused by stroke | No specified diagnostic criteria |
| 216 | Lei, Tao | Observation on the therapeutic effect of acupuncture combined with rehabilitation training on upper limb spastic paralysis after stroke | No specified diagnostic criteria |
| 217 | Rongyan, Zeng | Effect of electroacupuncture combined with physical therapy on balance function in patients with foot drooping after convalescent stroke | No specified diagnostic criteria |
| 218 | Jiajia, Chen | Clinical evaluation of electro-acupuncture at antagonizing muscle points combined with rehabilitation training in the treatment of hemiplegic spasticity after cerebral apoplexy | No specified diagnostic criteria |
| 219 | Zhi, Ming | Effects of rehabilitation training combined with electro-acupuncture on antagonizing muscle points on stroke patients with hemiplegic spasm | No specified diagnostic criteria |
| 220 | Yaqiao, Wang | Effect of acupuncture combined with rehabilitation training on the recovery effect of upper limb spastic hemiplegia after stroke | No specified diagnostic criteria |
| 221 | Zhaoxin, Xia | Analysis of Curative Effect of Acupuncture Combined with Rehabilitative Therapy in Treatment of Spastic Hemiplegia after Cerebrovascular Accident | No specified diagnostic criteria |
| 222 | Zhiqiang, Zhang | Observation of limb spasticity in patients with hemiplegia after cerebrovascular accident treated with acupuncture and rehabilitation therapy | No specified diagnostic criteria |
| 223 | Hongxia, Fan | Rehabilitation combined with acupuncture for early interventional treatment of 50 cases of cerebral infarction with hemiplegic spasm | No specified diagnostic criteria |
| 224 | Feiwei, Liao | Observation on the effect of electro-acupuncture at antagonistic muscle points combined with new Bobath technique in the treatment of lower extremity spasm after stroke | No specified diagnostic criteria |
| 225 | Shamisinur Abipol | Observation on the curative effect of acupuncture combined with rehabilitation training in the treatment of upper limb spastic paralysis after stroke | No specified diagnostic criteria |
| 226 | Changming, Jin | Clinical effect of acupuncture and moxibustion on spastic hemiplegia after stroke | No specified diagnostic criteria |
| 227 | Yiting, Xia | The effect of acupuncture combined with rehabilitation training in the treatment of upper limb spastic paralysis after stroke | No specified diagnostic criteria |
| 228 | Xinlong, Xu | Therapeutic effect of Jin’s three-needle on patients with spastic hemiplegia after stroke | No specified diagnostic criteria |
| 229 | Xinmin, Pan | Application of Santongjing antispasmodic acupuncture combined with contralateral limb movement induced by ipsilateral limb movement in patients with post-stroke hemiplegia | No specified diagnostic criteria |
| 230 | Dongxia, Li | Clinical study on the treatment of limb spasm after cerebral infarction with acupuncture at Jing point | No specified diagnostic criteria |
| 231 | Chaobo, Wei | Clinical Observation on Treatment of Spastic Paralysis After Apoplexy Treated by Yin-Yang Acupuncture | No specified diagnostic criteria |

**Continued**

| **No.** | **First Author** | **Title** | **Excluded Reason** |
| --- | --- | --- | --- |
| 232 | Mingfang, Ni | Clinical Observation of Acupuncture Combined with Rehabilitation Training in Treating Apoplexy Spastic Hemiplegia | No specified diagnostic criteria |
| 233 | Xiubing, Li | Observation of curative effect of acupuncture combined with rehabilitation training in the treatment of stroke spastic hemiplegia | No specified diagnostic criteria |
| 234 | Songwu, Ma | Efficacy of acupuncture combined with rehabilitation training in the treatment of post-stroke spastic hemiplegia | No specified diagnostic criteria |
| 235 | Bifen, Wu | Comparison of the effects of acupuncture and comprehensive rehabilitation exercise in the treatment of post-stroke spastic hemiplegia | No specified diagnostic criteria |
| 236 | Yue, Ding | Observation on the curative effect of acupuncture combined with rehabilitation training in the treatment of upper limb spastic paralysis after stroke | No specified diagnostic criteria |
| 237 | Linqing, Jin | Clinical observation of abdominal acupuncture combined with exercise therapy in treating spastic hemiplegia after stroke | No specified diagnostic criteria |
| 238 | Man, Yang | Xingnao Kaiqiao acupuncture combined with rehabilitation training and western medicine in the treatment of 42 cases of upper limb spastic paralysis after stroke | No specified diagnostic criteria |
| 239 | Dongdong, Xu | Clinical effect of rehabilitation training combined with electroacupuncture in patients with post-stroke spastic hemiplegia | No specified diagnostic criteria |
| 240 | Xiaobo, Yang | Effects of acupuncture on muscle spasm in stroke patients with hemiplegia | No specified diagnostic criteria |
| 241 | Wenquan, Yu | Observation on the curative effect of electroacupuncture stimulating antagonistic muscle acupoints combined with rehabilitation training in treatment of hemiplegia spasm after stroke | No specified diagnostic criteria |
| 242 | Fenglin, Zhang | Effect of Jin's three-needle therapy combined with rehabilitation training in the treatment of spastic hemiplegia caused by stroke | No specified diagnostic criteria |
| 243 | Hailin, Chen | Analysis of acupuncture and rehabilitation nursing for upper limb spasm after stroke | No specified diagnostic criteria |
| 244 | Limei, Chen | Analysis of application effect of modified bobath rehabilitation training combined with spasm three-needle in patients with limb spastic paralysis | No specified diagnostic criteria |
| 245 | Mingjun, Hao | Curative efficacy of acupuncture combined with western medicine in treatment of stroke spastic paralysis | No specified diagnostic criteria |
| 246 | Fangrui, Li | Observation on the curative effect of acupuncture combined with rehabilitation training in the treatment of upper limb spastic paralysis after stroke | No specified diagnostic criteria |
| 247 | Wangli, Song | Observation on the effect of acupuncture and rehabilitation nursing on upper limb spasm after stroke | No specified diagnostic criteria |
| 248 | Pan, Yang | Effect of Xingnao Kaiqiao acupuncture on post-stroke spastic hemiplegia patients | No specified diagnostic criteria |
| 249 | Minxiang, Chen | Study on the effectiveness of rehabilitation training combined with acupuncture in the treatment of upper extremity spasm after cerebral infarction | No specified diagnostic criteria |

**Continued**

| **No.** | **First Author** | **Title** | **Excluded Reason** |
| --- | --- | --- | --- |
| 250 | Wang, C. | Clinical curative effect of electric acupuncture on acute cerebral infarction: a randomized controlled multicenter trial | No specified diagnostic criteria |
| 251 | Zhang, S. | Acupuncture efficacy on ischemic stroke recovery: multicenter randomized controlled trial in China | No specified diagnostic criteria |
| 252 | Chen, L. | Additional effects of acupuncture on early comprehensive rehabilitation in patients with mild to moderate acute ischemic stroke: a multicenter randomized controlled trial | No specified diagnostic criteria |
| 253 | Liu, C. H. | Acupuncture for a first episode of acute ischaemic stroke: an observer-blinded randomised controlled pilot study | No specified diagnostic criteria |
| 254 | Liao, H. Y. | Clinical evaluation of acupuncture as treatment for complications of cerebrovascular accidents: a randomized, sham-controlled, subject- and assessor-blind trial | No specified diagnostic criteria |
| 255 | Han-Hwa Hu | A Randomized Controlled Trial on the Treatment for Acute Partial Ischemic Stroke with Acupuncture | No specified diagnostic criteria |
| 256 | S. Sallström | Acupuncture in the treatment of stroke patients in the subacute stage: a randomized, controlled study | No specified diagnostic criteria |
| 257 | Barbro B. Johansson | Acupuncture and Transcutaneous Nerve Stimulation in Stroke Rehabilitation  A Randomized, Controlled Trial | No specified diagnostic criteria |
| 258 | Pei Jian | The Effect of Electro-Acupuncture on Motor Function Recovery in Patients with Acute Cerebral Infarction: A Randomly Controlled Trial | No specified diagnostic criteria |
| 259 | Yuan, Gao | Effects of acupuncture combined with rehabilitation therapy on nerve,motor function and quality of life in patients with hemiplegia after stroke | No specified diagnostic criteria |
| 260 | Haicheng, Gong | Effect of acupuncture and moxibustion combined with functional exercise on cerebral apoplexy hemiplegia | No specified diagnostic criteria |
| 261 | Zhan, J. | Effect of abdominal acupuncture combined with routine rehabilitation training on shoulder-hand syndrome after stroke: A randomized controlled trial | No specified diagnostic criteria |
| 262 | Mayu, Hang | Clinical Observation of Combined Rehabilitation and Jin's Acupuncture in Treating Spastic Hemiplegia After Apoplexy | No site specified for MAS assessment |
| 263 | Rilong, Huang | Clinical Observation on Acupuncture Combined with Rehabilitation Training in Treating Apoplexy Hemiplegia Spasticity | No site specified for MAS assessment |
| 264 | Chengyan, Wang | Observation on the clinical effect of abdominal needle in the treatment of muscle spasm after stroke | No site specified for MAS assessment |
| 265 | Li, Shen | Clinical Observation on 40 Cases of Post-stroke Spastic Paralysis Treated by Acupuncture at Meridian and Sinew Nodes Combined with Rehabilitation Therapy | No site specified for MAS assessment |
| 266 | Deyu, Hu | Clinical Study of Electro-acupuncture Combined with Rehabilitation Training for Apoplectic Spasticity Hemiplegia | No site specified for MAS assessment |

**Continued**

| **No.** | **First Author** | **Title** | **Excluded Reason** |
| --- | --- | --- | --- |
| 267 | Bo, Lei | Electroacupuncture Therapy Guided by Brunnstrom Theory for Spastic Hemiplegia | No site specified for MAS assessment |
| 268 | Runjie, Sun | A clinical study on the treatment of “Jin gou diao yu” acupuncture for spastic paralysis after ischemic stroke | No site specified for MAS assessment |
| 269 | Jian, Zhang | Clinical efficacy of contralateral puncture with rehabilitation therapy for postapopletic spastic hemiplegia | No site specified for MAS assessment |
| 270 | Gan, Huang | Effect of“Jingjin”Needling Method on Spasticity of Hemiplegia in Stroke | No site specified for MAS assessment |
| 271 | Caiqin, Sun | Effects of acupuncture combined with early rehabilitation therapy on limb spasticity in stroke patients | No site specified for MAS assessment |
| 272 | Guoshu, Wang | Clinical Study on Acupuncture at Cranial Sutures plus Rehabilitation Training for Post-stroke Spastic Palsy | No site specified for MAS assessment |
| 273 | Ming, Wang | Clinical research on acupuncture for functional rehabilitation of stroke patients with spasticity | No site specified for MAS assessment |
| 274 | Zhanyou, Xue | Observation on the curative effect of acupuncture combined with baclofen in the treatment of post-stroke muscle spasm | No site specified for MAS assessment |
| 275 | Jiazong, Ye | Clinical observation of electro-acupuncture stimulation of acupoints on the side of antagonistic muscles combined with rehabilitation training in the treatment of post-stroke patients with increased muscle tone | No site specified for MAS assessment |
| 276 | Yi, Ying | Clinical observation of acupuncture combined with Bobath therapy to reduce muscle tension after stroke | No site specified for MAS assessment |
| 277 | Jiao, Liu | Efficiency observation of spasmolysis and rectifying hemiplegia acupuncture combined with new Bobath technology on apoplectic spastic hemiplegia | No site specified for MAS assessment |
| 278 | Guochang, Quan | Clinical observation of electroacupuncture combined with Bobath therapy in the treatment of post-stroke spastic paralysis | No site specified for MAS assessment |
| 279 | Jing, Xu | Clinical observation of acupuncture with rehabilitation therapy on apoplectic spastic hemiplegia | No site specified for MAS assessment |

**Continued**

| **No.** | **First Author** | **Title** | **Excluded Reason** |
| --- | --- | --- | --- |
| 280 | Ronglin,Sui | Effects of acupuncture combined with Bobath therapy on limb spasm after stroke | No site specified for MAS assessment |
| 281 | Yue, Zhao | Clinical Efficacy of Eye Acupuncture for Stroke and the Correlation Between Keap1-Nrf2/ARE Signaling Pathway and Cellular Oxidative Stress | No site specified for MAS assessment |
| 282 | Sisi, Wei | Clinical observation of eye acupuncture therapy on spastic hemiplegia after stroke | No site specified for MAS assessment |
| 283 | Qingping, Luo | Clinical observation on treatment of spastic paralysis after stroke with acupuncture combined with contemporary rehabilitation technique | No site specified for MAS assessment |
| 284 | Jun, Liu | Clinical observation on treatment of spasticity poststroke byelectric acupuncture with motion point and Yang meridian | No site specified for MAS assessment |
| 285 | Zhengchao, He | Treatment of post-stroke spasticity with combinative therapy of scalp acupuncture and Bobath | No site specified for MAS assessment |
| 286 | Kefeng, Ni | Clinical Observation of Recuperative Effect of Opposing Needling Accompanying Continual Static Stretch in Treating High Muscular Tension of Apoplectic Hemiplegia | No site specified for MAS assessment |
| 287 | Qian, Wang | Clinical study on complex facilitation technique of electro-acupuncturing antagonistic muscle acupoint in treating extremital spasm caused by cerebral infarction hemiplegia | No site specified for MAS assessment |
| 288 | Daquan, Zhan | The study of clinical application of Jin’s 3-needle and occupational therapy on treating spastic paralysis after cerebral apoplexy | No site specified for MAS assessment |
| 289 | Wei, Shen | Effect of Jinsanzhen therapy combined with rehabilitation training for treatment of post-stroke spasticity | No site specified for MAS assessment |
| 290 | Yangyi, Chen | The acupuncture combined with rehabilitation therapy for stroke patients with limb spasticity | No site specified for MAS assessment |
| 291 | Changfeng, Ta | Clinical study of post-stroke spastic paralysis treated with scalp acupuncture and body acupuncture combined with Bobath skill | No site specified for MAS assessment |

**Continued**

| **No.** | **First Author** | **Title** | **Excluded Reason** |
| --- | --- | --- | --- |
| 292 | Ping, Wu | Research of efficacy assessment of post-stroke hemiplegic spasticity treated with acupuncture and rehabilitation: a multi-centre randomized controlled trial | No site specified for MAS assessment |
| 293 | Fengjuan, Lu | The combination of acupuncture Bobath therapy tendon junction of spastic paralysis after stroke clinical efficacy | No site specified for MAS assessment |
| 294 | Yi, Xia | Observation on the curative effect of scalp acupuncture combined with rehabilitation training in the treatment of stroke spastic paralysis | No site specified for MAS assessment |
| 295 | Hongyan, Chen | Clinical observation on Quanjing acupuncture therapy combined with rehabilitation training for treatmemnt of hemiplegia and spasticity caused by apoplexy. | No site specified for MAS assessment |
| 296 | Qin, Chen | Influence of reconciling yin and yang acupuncture on spastic paralysis after stroke | No site specified for MAS assessment |
| 297 | Yanqing, Lu | A clinical observation on Jin’s three-needle therapy combined with rehabilitation for 40 cases of post-spastic hemiplegia | No site specified for MAS assessment |
| 298 | Rong, Meng | Clinical observation of electro-acupuncture combined with rehabilitation in the treatment of stroke spastic hemiplegia | No site specified for MAS assessment |
| 299 | Haitao, Yang | The clinical study of temporal three-needle combined with spasm three-needle therapy on spastic hemiplegia after stroke | No site specified for MAS assessment |
| 300 | Cai, Y. | Electroacupuncture for Poststroke Spasticity: results of a Pilot Pragmatic Randomized Controlled Trial | No site specified for MAS assessment |
| 301 | Jingbao, Qiu | Clinical effect of acupuncture and cross vein acupoints in the treatment of spastic hemiplegia after stroke | No site specified for MAS assessment |
| 302 | Huimin, Qiu | Clinical study of acupuncture on spastic hemiplegia after cerebral apoplexy | No site specified for MAS assessment |
| 303 | Congyin, Ren | Clinic Effect,Spasticity Index and Ashworth Score of Acupuncture Combined with Rehabilitation Training on Limb Spasm After Stroke | No site specified for MAS assessment |
| 304 | Zhengyang, Zhang | Clinical observation on the treatment of spastic hemiplegia after cerebral apoplexy by purging Yin and reinforcing Yang acupuncture combined with neuromuscular electrical stimulation | No site specified for MAS assessment |

**Continued**

| **No.** | **First Author** | **Title** | **Excluded Reason** |
| --- | --- | --- | --- |
| 305 | Xiaolong, Lv | Clinical study of purging Yin and reinforcing Yang acupuncture combined with neuromuscular electrical stimulation in the treatment of spastic hemiplegia caused by stroke | No site specified for MAS assessment |
| 306 | Yujing, Deng | Acupuncture Jiaji point combined with exercise therapy for the treatment of hemiplegia spasticity after stroke | Random method not specified |
| 307 | Fei, Xu | Observation of curative effect of acupuncture combined with rehabilitation training in the treatment of limb spasm after cerebral infarction | Random method not specified |
| 308 | Yueyue, Huang | Evaluation of the effect of acupuncture combined with rehabilitation training on patients with post-stroke limb spasm | Random method not specified |
| 309 | Ji, Li | Clinical observation of acupuncture combined with rehabilitation training in the treatment of spastic foot varus in stroke patients | Random method not specified |
| 310 | Yufeng, Li | Clinical study of electro-acupuncture combined with rehabilitation training on spasticity of hemiplegia after stroke | Random method not specified |
| 311 | Ji, Wang | Clinical application of real-time shear wave ultrasound elastography in evaluating acupuncture for post-stroke muscle spasm | Random method not specified |
| 312 | Lina, Zhao | Clinical study on acupuncture in the recovery period of ischemic stroke(type of blood stasis due to qi deficiency) | Random method not specified |
| 313 | Huarong, Feng | Effective observation on treating upper limb spasticity after stroke by acupuncture with rehabilitation nursing | Random method not specified |
| 314 | Runjie, Sun | Clinical study of post-stroke upper limb spasmodic hemiplegia treated with jingou diaoyu needling technique and Bobath therapy | Random method not specified |
| 315 | Xuemei, Wang | Clinical Observation on 90 Cases of Apoplexy Limb Spasm Treated by contralateral Needling | Random method not specified |
| 316 | Binbin, Zhang | Observation on the curative effect of comprehensive rehabilitation on the increased muscle tone of upper limbs after stroke | Random method not specified |
| 317 | Lu, Zhang | Effects of acupuncture and moxibustion on limb motor function and activities of daily living in elderly patients with post-stroke spastic hemiplegia | Random method not specified |
| 318 | Runjie, Sun | Clinical study of post-stroke upper limb spasmodic hemiplegia treated with jingou diaoyu needling technique and Bobath therapy | Random method not specified |
| 319 | Xuemei, Wang | Clinical Observation on 90 Cases of Apoplexy Limb Spasm Treated by contralateral Needling | Random method not specified |
| 320 | Binbin, Zhang | Observation on the curative effect of comprehensive rehabilitation on the increased muscle tone of upper limbs after stroke | Random method not specified |
| 321 | Lu, Zhang | Effects of acupuncture and moxibustion on limb motor function and activities of daily living in elderly patients with post-stroke spastic hemiplegia | Random method not specified |
| 322 | Runjie, Sun | Clinical study of post-stroke upper limb spasmodic hemiplegia treated with jingou diaoyu needling technique and Bobath therapy | Random method not specified |
| 323 | Xuemei, Wang | Clinical Observation on 90 Cases of Apoplexy Limb Spasm Treated by contralateral Needling | Random method not specified |

**Continued**

| **No.** | **First Author** | **Title** | **Excluded Reason** |
| --- | --- | --- | --- |
| 324 | Chi, Zhang | Treatment of severe spasticity of hemiplegic limbs after cerebral hemorrhage | Random method not specified |
| 325 | Shugen, Zhang | Study on the treatment of upper extremity spasticity after stroke by Yan's manipulation acupuncture combined with Bobath technique | Random method not specified |
| 326 | Xiaofeng, Fu | Curative effect of acupuncture and moxibustion in adjuvant treatment for patients with post-stroke limb paralysis and its effect on limb motor function and living ability | Random method not specified |
| 327 | Yanli, Wei | Observation on the curative effect of acupuncture combined with rehabilitation training in the treatment of post-stroke limb spasm | Random method not specified |
| 328 | Mei, Zhang | Clinical Observation on Acupuncture Combined with Rehabilitation Training in the Treatment of Ischemic Stroke with Upper Limb Spasm | Random method not specified |
| 329 | Yanli, Zhang | Efficacy of reconciling yin and yang acupuncture combined with Bobath therapy on spastic paralysis after stroke | Random method not specified |
| 330 | Zizhen, Zhang | The Influence of Acupuncture on Lower Extremity Muscle Tension of Patients with Hemiplegia After Cerebral Infarction | Random method not specified |
| 331 | Chi, Zhang | Treatment of severe spasticity of hemiplegic limbs after cerebral hemorrhage | Random method not specified |
| 332 | Shugen, Zhang | Study on the treatment of upper extremity spasticity after stroke by Yan's manipulation acupuncture combined with Bobath technique | Random method not specified |
| 333 | Xiaofeng, Fu | Curative effect of acupuncture and moxibustion in adjuvant treatment for patients with post-stroke limb paralysis and its effect on limb motor function and living ability | Random method not specified |
| 334 | Yanli, Wei | Observation on the curative effect of acupuncture combined with rehabilitation training in the treatment of post-stroke limb spasm | Random method not specified |
| 335 | Mei, Zhang | Clinical Observation on Acupuncture Combined with Rehabilitation Training in the Treatment of Ischemic Stroke with Upper Limb Spasm | Random method not specified |
| 336 | Chi, Zhang | Treatment of severe spasticity of hemiplegic limbs after cerebral hemorrhage | Random method not specified |
| 337 | Shugen, Zhang | Study on the treatment of upper extremity spasticity after stroke by Yan's manipulation acupuncture combined with Bobath technique | Random method not specified |
| 338 | Xiaofeng, Fu | Curative effect of acupuncture and moxibustion in adjuvant treatment for patients with post-stroke limb paralysis and its effect on limb motor function and living ability | Random method not specified |
| 339 | Yanli, Wei | Observation on the curative effect of acupuncture combined with rehabilitation training in the treatment of post-stroke limb spasm | Random method not specified |
| 340 | Mei, Zhang | Clinical Observation on Acupuncture Combined with Rehabilitation Training in the Treatment of Ischemic Stroke with Upper Limb Spasm | Random method not specified |

**Continued**

| **No.** | **First Author** | **Title** | **Excluded Reason** |
| --- | --- | --- | --- |
| 341 | Panzhi, Lv | Rehabilitation of spastic hemiplegia after stroke | Random method not specified |
| 342 | Xiaofeng, Zhao | Efficacy of “Remissive Stage-reinforcing and Acute Stage-reducing” Acupuncture for Treating 30 Apoplectic Myospasm Patients | Random method not specified |
| 343 | Li, Li | Effect of electroacupuncture combined with baclofen for post-stroke spasticity | Random method not specified |
| 344 | Defu, Yang | Effects of combined acupuncture combined with Bobath therapy on spasticity and motor function of stroke hemiplegia | Random method not specified |
| 345 | Min, Yu | Observation of curative effect of electroacupuncture combined with rehabilitation training in improving spasticity in hemiplegic patients | Random method not specified |
| 346 | Shuwei, Li | Clinical study on combined treatment of post-stroke myospasm with acupuncture and rehabilitation training | Random method not specified |
| 347 | Yingzi, Wang | Electroacupuncture on antagonistic muscle points combined with exercise therapy for post-stroke muscle spasm | Random method not specified |
| 348 | Zhengyan, Wang | Clinical observation on the effect of scalp acupuncture combined with wax therapy on limb muscle tension in patients with cerebral infarction | Random method not specified |
| 349 | Qian, Xue | Effect of balance muscle tension acupuncture on motor function rehabilitation of hemiplegic patients with scleroparesis | Random method not specified |
| 350 | Xiaolian, Yang | Clinical observation of acupuncture combined with rehabilitation training in the treatment of post-stroke limb spasm | Random method not specified |
| 351 | Ying, Bi | Clinical study of thick needle therapeutic method in treating the spastic state of apoplectic patients | Random method not specified |
| 352 | Weijun, Gong | Electro-acupuncture at Zusanli(ST 36) to improve lower extremity motor function in sensory disturbance patients with cerebral stroke A randomized controlled study of 240 cases | Random method not specified |
| 353 | Xianying, Zeng | The acupuncture and moxibustion treatment apoplexy hemiparalysis curative effect analyzed | Random method not specified |
| 354 | Xin, Chen | Acupuncture therapy combined with contemporary rehabilitation techniques for treatment of hemiplegia and spasticity caused by apoplexy | Random method not specified |
| 355 | Jingming, Jiang | Clinical Study on Acupuncture Treatment Methods for Spasticity of Paralysed Limbs after Stroke | Random method not specified |
| 356 | Chengxu, Jin | Clinical study of acupuncture combined with rehabilitation in the treatment of upper limb spasticity in stroke hemiplegia | Random method not specified |
| 357 | Ying, Zhang | Clinical efficacy of spasmolytic acupuncture therapy for patients in convulsive stage of apoplectic sequelae | Random method not specified |
| 358 | Amir Hooman Kazemi | Clinical observation of acupuncture on spasticity of lower extremities after stroke | Random method not specified |

**Continued**

| **No.** | **First Author** | **Title** | **Excluded Reason** |
| --- | --- | --- | --- |
| 359 | Liubo, Fan | Effect of electroacupuncture and electromyographic biofeedback therapy on H wave and M wave in spastic hemiplegia patients | Random method not specified |
| 360 | Xiaohua, He | Efficacy analysis of scalp acupuncture combined with antispasmodic manipulation in the treatment of post-stroke hemiplegia spasticity | Random method not specified |
| 361 | Minghua, Huang | Curative effect observation of acupuncture point selection combined with electromyography biofeedback in treating 120 cases of post-stroke lower extremity spasm | Random method not specified |
| 362 | Hongxing, Li | Clinical Observation of Acupuncture at Acupoints on the Side of Antagonistic Muscles in Treating Limb Spasm After Apoplexy | Random method not specified |
| 363 | Changfeng, Ta | Clinical study on effect of "Eliminating blood stasis and promoting tissue regeneration"acupuncture combined with rehabilitation training in treating spastic paralysis of the patient after operation of hypertensive basal ganglia hemorrhage | Random method not specified |
| 364 | Qingning, Xiao | Evaluation of the effect of acupuncture combined with rehabilitation training on upper limb spasm after stroke in 25 cases | Random method not specified |
| 365 | Weiming, Zhang | Post-Apoplexy Spasm Treated with Acupuncture on Antagonistic Muscles Combined with Modern Rehabilitation Training Therapy | Random method not specified |
| 366 | Xiaohong, He | Efficacy of electrical stimulation of human acupoints on limb spasm and motor function in patients with cerebral infarction | Random method not specified |
| 367 | Xi, Jin | Observation on curative effect of acupuncture combined with modern rehabilitation training on limb spasm after stroke | Random method not specified |
| 368 | Junjie, Song | Study on diagnosis and treatment techniques of limb spasm after stroke | Random method not specified |
| 369 | Guoxin, Yao | Electroacupuncture on antagonistic muscle points combined with exercise therapy for post-stroke muscle spasm | Random method not specified |
| 370 | Qin, Chen | Therapeutic observation of 30 cases of post-stroke spastic paralysis treated by reconciling yin and yang acupuncture | Random method not specified |
| 371 | Wei, Gu | Therapeutic observation of 30 cases of post-stroke spastic paralysis treated by reconciling yin and yang acupuncture | Random method not specified |
| 372 | Jing, Ha | A clinical study of antagonistic muscle groups of acupoints ofacupuncture treatment of hemiplegic limb spasticity after stroke | Random method not specified |
| 373 | Jingwei, He | Clinical study on acupuncture combined with exercise therapy for spasticity caused by stroke | Random method not specified |
| 374 | Junhong, Huang | The Clinical Analysis of Acupuncture in the Treatment of Stroke Increased Muscle Tone | Random method not specified |
| 375 | Peng, Jiang | Clinical observation of acupuncture on corresponding points of antagonistic muscles combined with Bobath therapy in the treatment of hemiplegia spasticity | Random method not specified |
| 376 | Jiaying, Wang | Observation of curative effect of Hui acupuncture combined with rehabilitation training in improving spasticity of upper extremity hemiplegia after stroke | Random method not specified |

**Continued**

| **No.** | **First Author** | **Title** | **Excluded Reason** |
| --- | --- | --- | --- |
| 377 | Weihua, Zhong | Clinical observation of acupuncture at the nodes of meridians and tendons combined with antispasmodic techniques in the treatment of spastic hemiplegia | Random method not specified |
| 378 | Sudong, Han | 86 cases of post-stroke spastic paralysis treated by acupuncture at the nodes of meridians and tendons combined with rehabilitation therapy | Random method not specified |
| 379 | Shuting, Ting | Clinical Observation of Acupuncture Combined with Rehabilitation in Treating Post-Stroke Muscle Tension | Random method not specified |
| 380 | Lin, Qiu | Observation of curative effect of acupuncture combined with rehabilitation training in the treatment of post-stroke spastic hemiplegia | Random method not specified |
| 381 | Chunyan, Qu | Observation on the curative effect of acupuncture on stroke spastic hemiplegia | Random method not specified |
| 382 | Shilei, Si | Clinical observation on treating 60 cases of spastic hemiplegia following stroke by balance acupuncture therapy | Random method not specified |
| 383 | Fan, Sun | Effects of acupuncture at Jiaji point combined with rehabilitation training on the H-reflex of lower extremities in patients with stroke | Random method not specified |
| 384 | Fink, M. | Needle acupuncture on chronic poststroke leg spasticity | Random method not specified |
| 385 | Shifen, Xu | Clinical Observation of Jin's Three-needle Acupuncture plus Rehabilitation for Post-stroke Spastic Hemiplegia | Random method not specified |
| 386 | Liubo, Fan | Application of Electroacupuncture plus Movement Therapy in Recovering Neurologic Function of Patients with Spastic Hemiplegia | quasi-RCT |
| 387 | Youqiang, Fan | Evaluation of the effect of acupuncture combined with rehabilitation training in the treatment of stroke | quasi-RCT |
| 388 | Lin, Han | Clinical Observation of 30 Cases of Postapoplectic Finger Contracture Treated with Acupuncture | quasi-RCT |
| 389 | Zhouhong, Wang | Clinical observation on acupuncture plus motor therapy for postapoplectic spastic hemiplegia | quasi-RCT |
| 390 | Xianping, Huang | Clinical observation on the rehabilitation effect of acupuncture and moxibustion combined with rehabilitation training on hemiplegia after stroke | quasi-RCT |
| 391 | Hongwei, Ren | Clinical Observation Electro-acupuncture Antagonistic Muscle Combine Rehabilitatian Therapy in treating 68 Cases with Muscle Tension Increased after Stroke | quasi-RCT |
| 392 | Lei, Xu | Acupuncture combined with rehabilitation training for the limb spasm after stroke | quasi-RCT |
| 393 | Wei, Zhai | 27 cases of limb spasm after ischemic stroke treated by Bobath combined with acupuncture and somatosensory sound wave | quasi-RCT |
| 394 | Wei, Zhai | Effects of Tiaoshenzhijing comprehensive therapy on ADL in patients with ischemic stroke patients with limb spasm | quasi-RCT |
| 395 | Yonghui, Li | The clinical effects of whole acupuncture on cerebral infarction | quasi-RCT |

**Continued**

| **No.** | **First Author** | **Title** | **Excluded Reason** |
| --- | --- | --- | --- |
| 396 | Ailin, Wang | Therapeutic effect of acupuncture and moxibustion combined with rehabilitation therapy on hemiplegia after stroke | quasi-RCT |
| 397 | Yanbin, Yan | Clinical study of scalp acupuncture synchronous exercise therapy on spastic hemiplegia after stroke | quasi-RCT |
| 398 | Jia,Gong | Observation on the effect of acupuncture on upper antispasmodic points combined with passive rehabilitation exercise and manual massage in the treatment of ischemic stroke | quasi-RCT |
| 399 | Lijie, Gou | Observation on the curative effect of acupuncture and moxibustion in the treatment of post-stroke limb spasm | quasi-RCT |
| 400 | Yongqing, Pan | Treatment of 128 cases of post-stroke muscle tension increase by penetrating acupuncture and electro-acupuncture | quasi-RCT |
| 401 | Yi, Li | Effect of Acupuncture Cooperating with Bobath Approach on Spasticity after Stroke | quasi-RCT |
| 402 | Yunzhi, Ma | 36 cases of spastic paralysis after stroke treated by body acupuncture combined with exercise therapy | quasi-RCT |
| 403 | Guobin, Sheng | Acupuncture at the nodes of meridians and tendons combined with rehabilitation therapy for post-stroke spastic paralysis | quasi-RCT |
| 404 | Qinglun, Su | Clinical study of acupuncture combined with nerve block in the treatment of biceps spasm after stroke | quasi-RCT |
| 405 | Shuai, Tong | Observation on the efficacy of acupuncture at key acupoints combined with rehabilitation therapy for spasmodic hemiplegia after cerebral infarction | quasi-RCT |
| 406 | Lisheng, Zhao | Observation on the effect of acupuncture at Sanyang points combined with antispasmodic stretch technique in the treatment of 40 cases of cerebral hemiplegia patients with wrist joint spasm | quasi-RCT |
| 407 | Zhigang, Li | Efficacy Observation on Acupuncture for Spasm that Caused by Stroke | quasi-RCT |
| 408 | Qingling, Fang | Observation on the curative effect of acupuncture combined with rehabilitation training in the treatment of upper limb spastic paralysis after stroke | quasi-RCT |
| 409 | Hui, Xu | Clinical observation of electroacupuncture combined with rehabilitation training in the treatment of upper limb muscle spasm after stroke | quasi-RCT |

**Table S2: Study Characteristics of Included Trials**

| **Included trials** | **Sample size**  **(E/C)** | **Gender**  **(male/**  **female) (E/C)** | **Mean**  **age**  **(E/C)** | **Type of stroke**  **(E/C)** | **First**  **onset of stroke** | **Spasm**  **classification** | **Mean**  **duration**  **of stroke**  **(E/C)** | **Experimental group** | **Control**  **group** | **Outcomes** | **Adverse effect** | **Follow-up** |
| --- | --- | --- | --- | --- | --- | --- | --- | --- | --- | --- | --- | --- |
| Shi LT 2004a | 74(38/36) | E:29/9  C:28/8 | E:68.26±6.28  C:67.36±6.41 | ischemia  & hemorrhage | Y | CSI>12 | E:3.19±0.64w C:3.12±0.63w | EA+CR | CR | ③⑧ | / | / |
| Shi LT 2004b | 70(34/36) | E:26/10  C:28/8 | E:67.83±6.34  C:67.36±6.41 | ischemia  & hemorrhage | Y | CSI>12 | E:3.21±0.59w C:3.12±0.63w | EA | CR | ③⑧ | / | / |
| He J 2008 | 40(20/20) | E:15/5  C:12/8 | E:55.15±8.04  C:53.67±8.08 | ischemia  & hemorrhage | / | B2-4 | / | EA+CR | CR | ③⑤⑥⑧ | / | / |
| Zhang ZM 2008 | 60(30/30) | E:17/13  C:20/10 | E:65.13±9.76  C:62.43±11.19 | ischemia  & hemorrhage | / | MAS1^+^-4 | E:14.25±6.14d C:13.96±5.89d | MA | CR | ①②③④ | N | / |
| Chu GX 2009 | 60(30/30) | E:18/12  C:16/14 | E:60.37±10.81  C:60.77±10.65 | ischemia  & hemorrhage | / | MAS1-3 | E:42.33±16.72d C:40.2±14.06d | EA+CR | CR | ②③④⑧ | / | / |
| Jiao ZH 2009a | 61(31/30) | E:20/11  C:20/10 | E:57.03±10.52  C:61.93±8.68 | ischemia | ≤2 | MAS≥1 | E:41.65±17.96d  C:36.80±19.36d | MA+CR | CR | ①②③④ | / | / |
| Jiao ZH 2009b | 63(33/30) | E:19/14  C:20/10 | E:63.58±8.80  C:61.93±8.68 | ischemia | ≤2 | MAS≥1 | E:34.67±16.25d  C:36.80±19.36d | MA | CR | ①②③④ | / | / |
| Lu JY 2009 | 60(30/30) | E:19/11  C:17/13 | E:66.43±7.59 C:67.00±6.80 | ischemia  & hemorrhage | / | MAS1-3 | E:40.03±13.71d C:34.17±12.37d | EA+CR | CR | ①③④⑩ | / | / |
| Yu JY 2009 | 45(24/21) | E:16/8 C:14/7 | E:62.3±11.5 C:61.5±9.3 | ischemia  & hemorrhage | / | MAS≥1 | E:1.8±0.9y C:1.9±0.8y | EA+CR | CR | ① | / | / |
| Ni HH 2010 | 30(15/15) | E:11/4 C:10/5 | 40-79 | ischemia  & hemorrhage | / | MAS≥2 | / | MA+CR | CR | ②③ | / | / |
| Xu YL 2010 | 63(32/31) | E:17/15 C:14/17 | E:57±7.3 C:58±4.7 | ischemia  & hemorrhage | / | MAS1-3 | E:48.73±19.52d C:52.4±21.65d | MA | CR | ②③ | / | / |
| Yan W 2010 | 81(40/41) | E:18/22 C:22/19 | E:60.6±10.74 C:62.34±8.89 | ischemia | / | MAS≥1 | E:40.25±16.82d C:43.5±17.65d | EA+CR | CR | ①③④ | / | / |
| Chen M 2011 | 60(30/30) | E:23/7 C:21/9 | E:47-80 C:41-79 | ischemia | ≤2 | MAS1-3 | / | MA | CR | ①③④⑧⑨ | / | / |
| Wang LC 2011 | 60(30/30) | E:18/12 C:14/16 | E:58.1±6.2 C:62.4±5.6 | ischemia  & hemorrhage | / | MAS≥1 | E:4.78±1.229w C:4.24±0.89w | MA+CR | CR | ①③ | / | / |
| Wang LC 2011 | 73(37/36) | E:18/19 C:20/16 | E:58.1±6.2 C:62.4±5.6 | ischemia  & hemorrhage | / | MAS≥1 | E:4.78±1.22w C:4.24±0.89w | MA+CR | CR | ①③ | / | / |
| Wu W 2011 | 60(30/30) | E:17/13 C:16/14 | E:60±6 C:60±8 | ischemia | / | MAS≥1 | E:4.54y  C:4.76y | EA+CR | CR | ①③④ | / | / |
| Wu YH 2011 | 60(30/30) | E:16/14 C:15/15 | E:66.23±8.28 C:69±8.6 | ischemia | / | B2-4 | E:40.6±15.94d C:37.26±15.2d | MA+CR | CR | ① | / | / |
| Feng XG 2012 | 200(100/100) | E:49/51 C:58/42 | E:51.71±7.08 C:48.91±9.52 | ischemia  & hemorrhage | / | MAS1-3 | E:6.85±1.99w C:6.589±2.34w | MA+CR | CR | ①② | / | / |
| Hu DX 2013 | 56(28/28) | 23/33 | 39-75 | ischemia | Y | MAS1-4 | / | MA+CR | CR | ① | / | / |
| Wu N 2014a | 59(30/29) | E:18/12 C:19/10 | E:56.46±7.37 C:57.08±6.31 | ischemia  & hemorrhage | Y | MAS≥1 | E:34.81±14.51d C:33.6±15.84d | MA+CR | CR | ①③④ | N | / |
| Wu N 2014b | 58(29/29) | E:20/9 C:19/10 | E:56.22±6.46 C:57.08±6.31 | ischemia  & hemorrhage | Y | MAS≥1 | E:35.34±14.32d C:33.6±15.84d | MA | CR | ①③④ | N | / |
| Cheng P 2015 | 37(18/19) | E:8/10 C:11/8 | E:58.13±5.26 C:57.27±6.87 | ischemia  & hemorrhage | / | MAS=3 | E:52.4±18.36d C:48±16.45d | EA+CR | CR | ①③ | / | / |
| Qian S 2015 | 65(35/30) | E:24/11 C:21/9 | E:56.8±2.5 C:56.2±2.4 | ischemia  & hemorrhage | / | MAS≥1 | E:1.5±0.4y C:1.2±0.4y | MA+CR | CR | ①③④ | / | / |
| Xing XM 2015 | 60(30/30) | E:18/12 C:19/11 | E:62.6±7.1 C:60.0±8.7 | ischemia | / | MAS1-4 | E:56.2±20.99d C:57.3±23.79d | EA+CR | CR | ①②⑧ | / | / |
| Yao XH 2015 | 60(30/30) | E:16/14 C:17/13 | 40-70 | ischemia | Y | MAS1-3 | / | MA | CR | ②④⑧ | Y | Y |
| Huang D 2016 | 40(20/20) | E:12/8 C:12/8 | E:56.3±3.5 C:55.7±3.8 | ischemia  & hemorrhage | / | MAS≥1 | E:21.85±2.35d C:22.36±2.65d | EA+CR | CR | ①③ | / | / |
| Lin FC 2016 | 60(30/30) | E:20/10 C:21/9 | E:63.83±7.6 C:66.67±6.96 | ischemia  & hemorrhage | / | MAS1^+^-3 | E:52.97±23.30d C:54.07±23.79d | EA+CR | CR | ①②④ | Y | Y |
| Liu LL 2016 | 57(29/28) | E:15/14 C:15/13 | E:58.93±8.96 C:59.96±8.16 | ischemia  & hemorrhage | Y | MAS2-3 | E:28.52±6.87d C:29.43±7.33d | MA | CR | ①③④ | / | / |
| Qu F 2016a | 60(30/30) | E:14/16 C:17/13 | E:56.4±12.0 C:58.7±10.1 | ischemia  & hemorrhage | Y | MAS2-3 | E:45.5±17.2d C:47.3±16.4d | MA+CR | CR | ①②③④ | / | / |
| Qu F 2016b | 60(30/30) | E:15/15 C:17/13 | E:57.6±11.2 C:58.7±10.1 | ischemia  & hemorrhage | Y | MAS2-3 | E:46.8±16.6d C:47.3±16.4d | MA | CR | ①②③④ | / | / |
| Su CH 2016a | 60(30/30) | E:21/9 C:20/10 | E:61±6.8 C:61.9±6.6 | ischemia  & hemorrhage | / | MAS1^+^-3 | / | MA+CR | CR | ①②③④ | / | / |
| Su CH 2016b | 60(30/30) | E:23/7 C:20/10 | E:60.1±6.7 C:61.9±6.6 | ischemia  & hemorrhage | / | 1^+^-3 | / | MA | CR | ①②③④ | / | / |
| Ye BY 2016 | 60(30/30) | E:13/17 C:15/15 | E:60.1±9.7 C:61.7±10.2 | ischemia  & hemorrhage | / | MAS1-3 | E:3.7±1.6m C:3.8±1.5m | MA+CR | CR | ① | / | / |
| Ye WC 2016 | 60(30/30) | E:19/11 C:20/10 | E:65±7 C:65±7 | ischemia  & hemorrhage | Y | MAS1-3 | E:60.27±21.75d C:63.1±16.64d | MA+CR | CR | ①③④ | / | / |
| Jia CJ 2017 | 51(26/25) | E:14/12 C:15/10 | E:63±11 C:58±12 | ischemia  & hemorrhage | Y | MAS1-3 | E:41.4±11.0d C:44.1±13.2d | MA+CR | CR | ①③④ | N | / |
| Li BJ 2017 | 120(60/60) | E:32/28 C:35/25 | E:55.7±4.8 C:54.9±5.2 | ischemia | / | MAS1-3 | E:15.2±3.7d C:15.6±3.3d | EA+CR | CR | ②③ | / | / |
| Liu G 2017 | 120(60/60) | E:34/26 C:36/24 | E:55.4±7.69 C:55.27±7.96 | ischemia  & hemorrhage | Y | MAS≥1 | E:53.95±29.41d C:52.6±29.73d | EA+CR | CR | ①②③④ | / | / |
| Rong JF 2017 | 50(25/25) | E:14/11 C:12/13 | E:57.5±5.6 C:56.4±4.8 | ischemia  & hemorrhage | Y | MAS≥1^+^ | E:6.38±1.22m C:6.42±1.17m | MA+CR | CR | ①③ | / | / |
| Su CH 2017 | 60(30/30) | E:19/11 C:17/13 | E:58.08±7.3 C:59.08±7.32 | ischemia  & hemorrhage | / | MAS1-3 | E:2.03±1.02m C:2.01±1.01m | MA+CR | CR | ②③⑧ | / | / |
| Xie JJ 2017 | 90(45/45) | E:29/19 C:27/21 | E:52.5±10.84 C:50.7±11.4 | ischemia | / | MAS1-4 | E:42.3±10.2d C:40.1±11.42d | EA+CR | CR | ①③④ | / | / |
| Deng SJ 2018 | 44(22/22) | E: 14/8 C: 16/6 | E:55.77±4.74 C:57.38±4.88 | ischemia  & hemorrhage | Y | MAS1^+^-3 | E:45.94±7.36d C:46.38±6.76d | MA+CR | CR | ②③④ | / | / |
| Guan XR 2018 | 38(19/19) | E:13/6 C:15/4 | E:56.3±12.12 C:57.7±14.73 | ischemia | Y | MAS>1 | E:5.24±1.47m C:5.27±1.62m | MA+CR | CR | ①②③④ | / | / |
| Han ZX 2018 | 60(30/30) | E:19/11 C:14/16 | E:64.4±10.54 C:64.97±8.98 | ischemia | ≤2 | MAS1-3 | E:120.3±24.52d C:113±26.63d | MA+CR | CR | ① | / | / |
| Jia CJ 2018 | 66(34/32) | E:18/16 C:19/13 | E:61±12 C:57±12 | ischemia  & hemorrhage | Y | MAS1-3 | E:39.4±10.3d C:41.7±12.5d | MA+CR | CR | ①③④ | N | / |
| Qi LL 2018 | 60(30/30) | E:17/13 C:14/16 | E:64±10 C:65±9 | ischemia | ≤2 | MAS1-3 | E:118.6±27.9d C:113.5±26.6d | MA+CR | CR | ①③④ | / | / |
| Qing Y 2018 | 40(20/20) | E:14/6 C:11/9 | E:58.55±9.57 C:56.75±9.42 | ischemia  & hemorrhage | Y | MAS1-3 | E:2.85±1.79m C:3.10±1.65m | MA+CR | CR | ①③④ | / | / |
| Qiu LF 2018 | 60(30/30) | E:18/12 C:17/13 | E:57.90±8.75 C:59.80±9.68 | ischemia  & hemorrhage | Y | MAS≥1 | E:84.33±39.80d C:80.77±41.06d | MA+CR | CR | ②③④ | / | / |
| Wang J 2018a | 61(30/31) | E:15/15 C:16/15 | E:53.75±7.97 C:54.91±8.76 | ischemia  & hemorrhage | Y | MAS1-4 | E:50.43±16.93d C:54.91±8.76d | MA+CR | CR | ①③ | / | / |
| Wang J 2018b | 61(30/31) | E:17/13 C:16/15 | E:55.17±8.46 C:54.91±8.76 | ischemia  & hemorrhage | Y | MAS1-4 | E:50.26±16.34d C:54.91±8.76d | MA | CR | ①③ | / | / |
| Wang XY 2018 | 64(32/32) | E:19/13 C:22/10 | E:64.1±7.3 C:63.8±7.7 | ischemia  & hemorrhage | Y | MAS≥1 | E:42.7±13.9d C:43.1±13.6d | EA+CR | CR | ①②③ | / | / |
| Xu J 2018 | 49(25/24) | E:15/10 C:15/10 | E:59±12 C:60±11 | ischemia  & hemorrhage | Y | MAS1-3 | E:43.1±13.2d C:42.6±11.8d | MA+CR | CR | ①③④ | N | / |
| Fan WW 2019 | 64(32/32) | E:19/13 C:22/10 | E:64.09±7.25 C:63.84±7.67 | ischemia  & hemorrhage | Y | MAS≥1 | E:42.72±13.94d C:43.0±13.62d | EA+CR | CR | ①② | / | / |
| Feng WF 2019 | 60(30/30) | E:16/14 C:17/13 | E:57.5±1.5 C:58.3±1.2 | ischemia | / | MAS2-4 | / | EA+CR | CR | ①② | / | / |
| He J 2019 | 97(47/50) | E:33/17 C:29/21 | E:64.03±8.63 C:64.13±9.21 | ischemia  & hemorrhage | / | MAS≥1 | E:29.3±17.21d C:32.4±22.09d | MA | CR | ①③ | Y | / |
| Jiang XG 2019 | 60(30/30) | E:21/9 C:20/10 | E:61.23±9.34 C:60.97±9.34 | ischemia  & hemorrhage | Y | MAS1-2 | E:175.3±84.54d C:175±87.73d | MA+CR | CR | ①③④ | / | / |
| Li RQ 2019 | 60(30/30) | E:18/12 C:16/14 | E:41.20±13.68 C:39.00±11.57 | ischemia  & hemorrhage | Y | MAS1-3 | E:80.53±38.18d C:90.3±49.68d | EA+CR | CR | ①③④⑥⑦ | / | / |
| Ma ZY 2019 | 100(50/50) | E:35/15 C:36/14 | E:63±8 C:63±8 | ischemia  & hemorrhage | ≤2 | MAS1-3 | E:8.8±4.5w C:8.6±4.4w | MA+CR | CR | ①③ | / | / |
| Ma ZY 2019 | 40(20/20) | E:10/10 C:9/11 | E:67.2±5.7 C:65.4±4.8 | ischemia  & hemorrhage | Y | MAS1-4 | E:31.8±1.7d C:32.3±1.8d | MA+CR | CR | ①③④ | / | / |
| Rong XQ 2019 | 49(24/25) | E:17/7 C:16/9 | E:59.1±12.26 C:55.1±11.24 | ischemia  & hemorrhage | Y | MAS≥1 | E:4.84±3.96m C:3.84±3.47m | MA+CR | CR | ①②⑦ | / | / |
| Wang HQ 2019 | 59(30/29) | E:19/11 C:16/13 | E:56.7±7.02 C:59±7.51 | hemorrhage | / | B3-5 | E:59.53±17.49d C:55.7±15.78d | MA+CR | CR | ①③④⑤⑨ | / | / |
| Wang X 2019 | 180(90/90) | E:54/36 C:50/40 | E:63.2±10 C:62.8±12.4 | ischemia | / | MAS1-4 | E:44.5±8.2d C:42.8±6.9d | MA+CR | CR | ①③④ | / | / |
| Xu ZW 2019 | 60(30/30) | E:19/11 C:17/13 | E:64.57±8.10 C:63.20±8.00 | ischemia  & hemorrhage | ≤2 | MAS≥1 | E:3.51±0.92m C:3.16±0.43m | MA+CR | CR | ①④⑧ | / | / |
| Yang M 2019 | 72(36/36) | E:19/17 C:20/16 | E:64.53±7.59 C:64.08±8.57 | ischemia  & hemorrhage | / | MAS1-3 | E:64.28±40.01d C:64.53±40.41d | MA+CR | CR | ①②③④ | N | / |
| Zhang ZQ 2019a | 88(44/44) | E:31/13 C:31/13 | E:64.27±10.6 C:64.97±10.23 | ischemia  & hemorrhage | Y | MAS1^+^-3 | E:7.93±4.76m C:5.59±4.40m | MA+CR | CR | ①②④ | N | / |
| Zhang ZQ 2019b | 88(44/44) | E:31/13 C:30/14 | E:64.27±10.6 C:62.81±11.71 | ischemia  & hemorrhage | Y | MAS1^+^-3 | E:7.93±4.76m C:6.43±4.62m | MA | Sham MA | ①②④ | N | / |
| Zhu ZJ 2019 | 60(30/30) | E:17/13 C:19/11 | E:55.6±13.97 C:51.3±12.44 | ischemia  & hemorrhage | Y | MAS≥1 | E:42.57±22.43d C:42.5±20.49d | MA+CR | CR | ①③⑩ | / | / |
| Duan C 2020 | 96(49/47) | E:35/16 C:30/19 | E:58±10 C:60±11 | ischemia  & hemorrhage | Y | B2-5 | E:45.35±15.58d C:42.1±14.81d | MA | Sham MA | ①③④ | / | / |
| Feng WF 2020 | 60(30/30) | E:22/8 C:19/11 | E:59.67±9.65 C:60±10.15 | ischemia  & hemorrhage | / | MAS2-4 | E:2.5±1.96m C:2.93±2.18m | EA+CR | CR | ①⑨ | / | / |
| Guo QQ 2020 | 97(48/49) | E:29/19 C:28/21 | E:59.8±10.12 C:58.2±10.33 | ischemia  & hemorrhage | Y | MAS1-3 | E:120.1±87.99d C:115.0±84.59d | MA | WM | ①③④ | N | / |
| Hao Q 2020 | 72(36/36) | E:25/11 C:27/9 | E:56.16±9.7 C:54.48±8.5 | ischemia  & hemorrhage | Y | B≥2 | E:120.01±0.43d C:120.5±0.51d | EA+CR | CR | ①③④ | / | / |
| He X 2020 | 40(20/20) | E:15/5 C:13/7 | E:54.25±9.22 C:60.25±7.47 | ischemia  & hemorrhage | Y | MAS1-3 | E:58.85±33.45d C:68.1±48.25d | MA+CR | CR | ①②③ | N | / |
| Wang R 2020 | 70(35/35) | E:24/11 C:25/10 | E:60.4±8.286 C:59.2±9.771 | ischemia  & hemorrhage | / | MAS1-3 | E:52.54±41.62d C:55±39.082d | MA+CR | CR | ①②③④ | N | / |
| Wang XC 2020a | 67(33/34) | E:18/15 C:16/18 | E:51±9 C:53±10 | ischemia  & hemorrhage | / | MAS1-3 | E:64.8±38.2d C:61.9±32.8d | MA+CR | CR | ①③④ | / | / |
| Wang XC 2020b | 67(33/34) | E:17/16 C:16/18 | E:52±9 C:53±10 | ischemia  & hemorrhage | / | MAS1-3 | E:68.9±39.4d C:61.9±32.8d | MA | CR | ①③④ | / | / |
| Xie SM 2020 | 60(30/30) | E:21/9 C:17/13 | E:62.95±3.36 C:63.37±3.76 | ischemia  & hemorrhage | / | MAS1-3 | E:49.28±12.49d C:46.2±12.53d | EA+CR | CR | ①③④ | / | / |
| Yang Q 2020 | 60(30/30) | E:19/11 C:18/12 | E:60±6.98 C:59±7.23 | ischemia  & hemorrhage | / | MAS≥1 | E:11±2.23m C:12±2.21m | EA+CR | CR | ①③④⑤ | / | / |
| Yang S 2020 | 58(29/29) | E:20/9 C:22/7 | E:59.5±10.57 C:58.5±10.09 | ischemia  & hemorrhage | Y | MAS1-2 | E:46.66±40.53d C:45.9±34.65d | MA+CR | CR | ①③④⑧ | / | / |
| Yin ZL 2020 | 50(25/25) | E:16/9 C:17/8 | E:53±13 C:52±11 | ischemia | Y | B2-4 | E:1.5±0.4m C:1.5±0.5m | MA+CR | CR | ①③④ | / | / |
| Zhang Q 2020 | 50(25/25) | / | 16-65 | ischemia  & hemorrhage | Y | MAS1-3 | / | EA | WM | ①② | / | / |
| Zhu JM 2020 | 60(30/30) | E:13/17 C:16/14 | E:63±10 C:64±13 | ischemia  & hemorrhage | / | MAS≥1 | E:19.9±6.5d C:21.5±5.2d | MA+CR | CR | ①②③④⑤ | / | / |
| Deng FH 2021 | 120(60/60) | E:34/26 C:31/29 | E:65.1±10.04 C:61.5±11.31 | ischemia  & hemorrhage | / | MAS1-3 | E:33.21±10.53d C:37.81±11.3d | MA+CR | CR | ①③④ | / | / |
| Duan C 2021 | 80(41/39) | E:28/15 C:25/16 | E:58±10 C:61±11 | ischemia  & hemorrhage | Y | B2-5 | E:46.02±15.01d C:41.2±14.42d | EA+CR | CR | ①③④ | / | / |
| Duan YF 2021 | 60(30/30) | E:17/13 C:16/14 | E:56.2±9.4 C:55.3±10 | ischemia  & hemorrhage | / | MAS≥1 | E:31.1±9.5d C:32.6±8.7d | MA+CR | CR | ①③④ | / | / |
| Liu LD 2021 | 60(30/30) | E:21/9 C:19/11 | E:57.0±6.908 C:58.3±7.931 | ischemia  & hemorrhage | / | MAS1-3 | E:60.1±25.361d C:62±24.391d | MA+CR | CR | ①②③④⑦ | N | / |
| Song YJ 2021 | 64(32/32) | E:19/13 C:11/21 | E:54.83±8.76 C:57.22±4.81 | ischemia  & hemorrhage | Y | MAS≥2 | E:22.88±3.4d C:23.9±2.82d | MA+CR | CR | ③⑩ | / | / |
| Wang ZX 2021 | 60(30/30) | E:18/12 C:17/13 | E:72.8±10.31 C:73.2±10.19 | ischemia | / | MAS≥1 | E:13.82±2.19d C:13.65±2.28d | MA+CR | CR | ①②③④ | / | / |
| Wang T 2021 | 60(30/30) | E:18/12 C:19/11 | E:64.43±8.92 C:63.17±6.47 | ischemia | / | MAS1-4 | E:56.94±26.15d C:55.5±24.67d | MA+CR | CR | ①②③④ | / | / |
| Zhang L 2021 | 60(30/30) | E:17/13 C:16/14 | E:62.14±8.03 C:59.36±6.95 | ischemia  & hemorrhage | / | MAS1-3 | E:35.65±10.31d C:37.2±7.88d | EA+CR | CR | ①③④⑤⑥⑦ | / | / |
| Zhang SH 2021a | 140(70/70) | E:35/35 C:34/36 | E:57.3±15.49 C:58.9±12.35 | ischemia  & hemorrhage | Y | B2-5 | E:87.11±30.8d C:89.07±30.6d | MA+CR | CR | ①③④ | / | / |
| Zhang SH 2021b | 142(72/70) | E:40/32 C:34/36 | E:55.5±17.19 C:58.9±12.35 | ischemia  & hemorrhage | Y | B2-5 | E:82.51±32.87d C:89.07±30.6d | MA | CR | ①③④ | / | / |
| Zhang CX 2021 | 140(70/70) | E:35/35 C:34/36 | E:51±15 C:54±16 | ischemia  & hemorrhage | Y | MAS≥1 | E:36.22±14.83d C:33.5±14.76d | MA+CR | CR | ①③④ | / | Y |
| Huang H 2022 | 40(20/20) | E:9/11 C:11/9 | E:69±8 C:68±6 | ischemia  & hemorrhage | Y | MAS1^+^-3 | E:2.4±0.9m C:2.5±1m | EA+CR | CR | ③⑧ | / | / |
| Li JX 2022 | 144(72/72) | E:39/36  C:41/34 | E:66.15±7.12 C:64.2±6.67 | ischemia  & hemorrhage | Y | MAS1-3 | E:3.45±1.22m C:3.7±1.64m | MA+CR | CR | ①③④ | / | / |
| Ren CY 2022 | 60(30/30) | E:15/15 C:21/9 | E:76.85±7.97 C:77.93±7.52 | ischemia  & hemorrhage | / | MAS=4 | E:2.17±0.96m C:2.05±0.73m | MA+CR | CR | ①⑧ | / | / |
| Guo LL 2022 | 120(60/60) | 34/26 32/28 | 62.34±3.15 63.39±3.34 | ischemia | ≤2 | MAS≥2 | 3.28±0.48m 3.23±0.44m | MA+CR | CR | ①③④⑨ | / | / |
| Ma AF 2022 | 84(42/42) | 30/12 32/10 | 61±6 60±6 | ischemia& hemorrhage | ≤2 | MAS≥1 | 27.8±3.8d 27.3±3.6d | MA+CR | CR | ①③⑤⑦⑧ | N | / |
| Zhang QS 2022 | 61(31/30) | 21/10 18/12 | 35-70 | ischemia  & hemorrhage | / | MAS1-2 | / | EA+CR | CR | ①③④ | Y | / |

Note: B: Brunnstrom; E: experimental group; C: control group; d:day; w:week; m:month; y:year; MA: manual acupuncture; EA: electroacupuncture; CR: conventional rehabilitation; WM: western medicine; /:not mentioned; Y:yes; N:no; ①MAS score; ②effective rate; ③Fugl-Myer Assessment, FMA; ④Barthel Index, BI ⑤integral electromyography, iEMG; ⑥co-contraction rate, CCR; ⑦root mean square, RMS; ⑧the clinical spasticity index, CSI; ⑨ratio of maximum H-reflex to maximum M response, H_max_/M_max_; ⑩CSS: composite spasticity scale.

**Table S3: STRICTA, Standards for Reporting Interventions in Controlled Trials of Acupuncture**

| **Studies** | **1. Acupuncture rationale** | | | **2. Details of needling** | | | | | | | **3. Treatment regimen** | | **4.**  **Co-interventions** | | **5.**  **Practitioner background** | **6. Control or comparator interventions** | |
| --- | --- | --- | --- | --- | --- | --- | --- | --- | --- | --- | --- | --- | --- | --- | --- | --- | --- |
|  | **1a** | **1b** | **1c** | **2a** | **2b** | **2c** | **2d** | **2e** | **2f** | **2g** | **3a** | **3b** | **4a** | **4b** | **5** | **6a** | **6b** |
| Shi LT 2004a | Yes | No | No | No | Yes | Yes | Yes | Yes | Yes | Yes | Yes | Yes | Yes | No | No | No | Yes |
| Shi LT 2004b | Yes | No | No | No | Yes | Yes | Yes | Yes | Yes | Yes | Yes | Yes | Yes | No | No | No | Yes |
| He J 2008 | Yes | No | No | No | Yes | Yes | Yes | Yes | Yes | Yes | Yes | Yes | Yes | No | No | No | Yes |
| Zhang ZM 2008 | Yes | No | No | No | Yes | No | No | Yes | Yes | Yes | Yes | Yes | Yes | No | No | No | Yes |
| Chu GX 2009 | Yes | No | No | No | No | No | Yes | Yes | Yes | Yes | Yes | Yes | Yes | No | No | No | Yes |
| Jiao ZH 2009a | Yes | No | No | No | No | Yes | Yes | Yes | Yes | Yes | Yes | Yes | Yes | No | No | No | Yes |
| Jiao ZH 2009b | Yes | No | No | No | No | Yes | Yes | Yes | Yes | Yes | Yes | Yes | Yes | No | No | No | Yes |
| Lu JY 2009 | Yes | No | No | Yes | Yes | No | No | Yes | Yes | Yes | Yes | Yes | Yes | No | No | No | Yes |
| Yu JY 2009 | Yes | No | No | No | Yes | No | Yes | Yes | Yes | No | Yes | Yes | Yes | No | No | No | Yes |
| Ni HH 2010 | Yes | No | No | No | Yes | Yes | Yes | Yes | Yes | Yes | Yes | Yes | Yes | No | No | No | Yes |
| Xu YL 2010 | Yes | No | No | No | No | No | No | Yes | Yes | Yes | Yes | Yes | Yes | No | No | No | Yes |
| Yan W 2010 | Yes | No | No | No | Yes | No | Yes | Yes | Yes | Yes | Yes | Yes | Yes | No | No | No | Yes |
| Chen M 2011 | Yes | Yes | No | Yes | No | Yes | Yes | Yes | Yes | Yes | Yes | Yes | Yes | No | No | No | Yes |
| Wang LC 2011 | Yes | No | No | No | No | No | Yes | Yes | Yes | Yes | Yes | Yes | Yes | No | No | No | Yes |
| Wang LC 2011 | Yes | No | No | No | No | No | Yes | Yes | Yes | Yes | Yes | Yes | Yes | No | No | No | Yes |
| Wu W 2011 | Yes | No | No | No | Yes | No | Yes | Yes | Yes | Yes | Yes | Yes | Yes | No | No | No | Yes |
| Wu YH 2011 | Yes | No | No | No | Yes | Yes | Yes | Yes | Yes | No | Yes | Yes | No | No | No | No | No |
| Feng XG 2012 | Yes | No | No | No | No | No | No | Yes | Yes | No | Yes | No | Yes | No | No | No | Yes |
| Hu DX 2013 | Yes | No | No | No | Yes | Yes | No | Yes | Yes | Yes | Yes | Yes | Yes | No | No | No | Yes |
| Wu N 2014a | Yes | No | No | No | Yes | No | Yes | Yes | Yes | Yes | Yes | Yes | Yes | No | No | No | Yes |
| Wu N 2014b | Yes | No | No | No | Yes | No | Yes | Yes | Yes | Yes | Yes | Yes | Yes | No | No | No | Yes |
| Cheng P 2015 | Yes | Yes | No | No | Yes | Yes | Yes | Yes | Yes | Yes | Yes | Yes | Yes | No | Yes | No | Yes |
| Qian S 2015 | Yes | No | No | No | No | No | No | Yes | Yes | Yes | Yes | Yes | No | No | No | No | No |
| Xing XM 2015 | Yes | No | No | No | No | No | No | Yes | Yes | No | Yes | Yes | Yes | No | No | No | Yes |
| Yao XH 2015 | Yes | No | No | No | Yes | No | Yes | Yes | Yes | Yes | Yes | Yes | Yes | No | No | No | Yes |
| Hung D 2016 | Yes | Yes | No | Yes | Yes | Yes | No | Yes | Yes | Yes | Yes | Yes | Yes | No | No | No | Yes |
| Lin FC 2016 | Yes | Yes | No | No | Yes | No | Yes | Yes | Yes | No | Yes | Yes | Yes | No | Yes | No | Yes |
| Liu LL 2016 | Yes | Yes | No | Yes | Yes | Yes | No | Yes | Yes | Yes | Yes | Yes | No | No | No | No | No |
| Qu F 2016a | Yes | No | No | No | Yes | No | No | Yes | Yes | Yes | Yes | Yes | Yes | No | No | No | Yes |
| Qu F 2016b | Yes | No | No | No | Yes | No | No | Yes | Yes | Yes | Yes | Yes | Yes | No | No | No | Yes |
| Su CH 2016a | Yes | No | No | No | No | No | No | Yes | Yes | No | Yes | Yes | Yes | No | No | No | Yes |
| Su CH 2016b | Yes | No | No | No | No | No | No | Yes | Yes | No | Yes | Yes | Yes | No | No | No | Yes |
| Ye BY 2016 | Yes | No | No | No | No | No | Yes | Yes | Yes | Yes | Yes | Yes | No | No | No | No | No |
| Ye WC 2016 | Yes | Yes | No | No | No | Yes | Yes | Yes | Yes | Yes | Yes | Yes | Yes | No | No | No | Yes |
| Jia CJ 2017 | Yes | Yes | No | Yes | Yes | Yes | No | Yes | Yes | Yes | Yes | Yes | Yes | No | No | No | Yes |
| Li BJ 2017 | Yes | No | No | No | No | Yes | Yes | Yes | Yes | Yes | Yes | Yes | Yes | No | No | No | Yes |
| Liu G 2017 | Yes | No | No | Yes | No | Yes | Yes | Yes | Yes | No | Yes | Yes | Yes | No | No | No | Yes |
| Rong JF 2017 | Yes | No | No | No | Yes | No | Yes | Yes | Yes | No | Yes | Yes | Yes | No | No | No | Yes |
| Su CH 2017 | Yes | No | No | No | Yes | No | No | Yes | Yes | Yes | Yes | Yes | Yes | No | No | No | Yes |
| Xie JJ 2017 | Yes | No | No | No | Yes | No | No | Yes | Yes | Yes | No | Yes | Yes | No | No | No | Yes |
| Deng SJ 2018 | Yes | Yes | No | No | No | Yes | Yes | Yes | Yes | No | Yes | Yes | Yes | No | No | No | Yes |
| Guan XR 2018 | Yes | Yes | No | No | Yes | No | Yes | Yes | Yes | Yes | Yes | Yes | Yes | No | No | No | Yes |
| Han ZX 2018 | Yes | Yes | No | No | Yes | Yes | No | Yes | Yes | Yes | Yes | Yes | Yes | No | Yes | No | Yes |
| Jia CJ 2018 | Yes | Yes | No | Yes | Yes | Yes | No | Yes | Yes | Yes | Yes | Yes | Yes | No | No | No | Yes |
| Qi LL 2018 | Yes | Yes | No | No | Yes | Yes | No | Yes | Yes | Yes | Yes | Yes | Yes | No | Yes | No | Yes |
| Qing Y 2018 | Yes | Yes | No | Yes | Yes | Yes | Yes | Yes | Yes | Yes | Yes | Yes | Yes | No | No | No | Yes |
| Qiu LF 2018 | Yes | No | No | No | Yes | No | No | Yes | Yes | Yes | Yes | Yes | Yes | No | No | No | Yes |
| Wang J 2018a | Yes | No | No | No | Yes | Yes | No | Yes | Yes | Yes | Yes | Yes | Yes | No | No | No | Yes |
| Wang J 2018b | Yes | No | No | No | Yes | Yes | No | Yes | Yes | Yes | Yes | Yes | Yes | No | No | No | Yes |
| Wang XY 2018 | Yes | No | No | Yes | No | Yes | No | Yes | Yes | Yes | Yes | Yes | Yes | No | No | No | Yes |
| Xu J 2018 | Yes | Yes | No | No | Yes | Yes | Yes | Yes | Yes | Yes | Yes | Yes | Yes | No | No | No | Yes |
| Fan WW 2019 | Yes | No | No | Yes | No | Yes | No | Yes | Yes | No | Yes | Yes | Yes | No | No | No | Yes |
| Feng WF 2019 | Yes | No | No | No | No | No | No | Yes | Yes | Yes | Yes | Yes | Yes | No | No | No | Yes |
| He J 2019 | Yes | No | No | No | Yes | Yes | Yes | Yes | Yes | Yes | Yes | Yes | Yes | No | No | No | Yes |
| Jiang XG 2019 | Yes | Yes | No | Yes | Yes | Yes | Yes | Yes | Yes | Yes | Yes | Yes | Yes | No | No | No | Yes |
| Li RQ 2019 | Yes | No | No | No | Yes | Yes | Yes | Yes | Yes | Yes | Yes | Yes | Yes | No | No | No | Yes |
| Ma ZY 2019 | Yes | No | No | No | Yes | Yes | No | Yes | Yes | Yes | Yes | Yes | Yes | No | No | No | Yes |
| Ma ZY 2019 | Yes | No | No | No | Yes | No | No | Yes | Yes | No | Yes | Yes | Yes | No | No | No | Yes |
| Rong XQ 2019 | Yes | Yes | No | No | Yes | Yes | Yes | Yes | Yes | Yes | Yes | Yes | Yes | No | No | No | Yes |
| Wang HQ 2019 | Yes | No | No | No | No | Yes | Yes | Yes | Yes | Yes | Yes | Yes | Yes | No | Yes | No | Yes |
| Wang X 2019 | Yes | No | No | No | No | No | Yes | Yes | Yes | No | Yes | Yes | Yes | No | No | No | Yes |
| Xu ZW 2019 | Yes | Yes | No | No | Yes | Yes | Yes | Yes | Yes | No | Yes | Yes | Yes | No | No | No | Yes |
| Yang M 2019 | Yes | Yes | No | Yes | No | Yes | No | Yes | Yes | Yes | Yes | Yes | Yes | No | No | No | Yes |
| Zhang ZQ 2019a | Yes | No | No | No | Yes | Yes | Yes | Yes | Yes | No | Yes | Yes | Yes | No | No | No | Yes |
| Zhang ZQ 2019b | Yes | No | No | No | Yes | Yes | Yes | Yes | Yes | No | Yes | Yes | Yes | No | No | No | Yes |
| Zhu ZJ 2019 | Yes | Yes | No | No | No | Yes | No | Yes | Yes | Yes | Yes | Yes | Yes | No | No | No | Yes |
| Duan C 2020 | Yes | No | No | No | Yes | Yes | Yes | Yes | Yes | Yes | Yes | Yes | Yes | No | No | No | Yes |
| Feng WF 2020 | Yes | No | No | No | Yes | No | No | Yes | Yes | Yes | Yes | Yes | Yes | No | No | No | Yes |
| Guo QQ 2020 | Yes | Yes | No | No | Yes | Yes | Yes | Yes | No | Yes | Yes | Yes | Yes | No | No | No | Yes |
| Hao Q 2020 | Yes | No | No | No | No | No | Yes | Yes | Yes | Yes | Yes | Yes | Yes | No | No | No | Yes |
| He X 2020 | Yes | Yes | No | No | Yes | No | Yes | Yes | Yes | Yes | Yes | Yes | Yes | No | No | No | Yes |
| Wang R 2020 | Yes | Yes | No | No | Yes | No | Yes | Yes | Yes | Yes | Yes | Yes | Yes | No | No | No | Yes |
| Wang XC2020a | Yes | No | No | No | Yes | Yes | Yes | Yes | Yes | Yes | Yes | Yes | Yes | No | No | No | Yes |
| Wang XC 2020b | Yes | No | No | No | Yes | Yes | Yes | Yes | Yes | Yes | Yes | Yes | Yes | No | No | No | Yes |
| Xie SM 2020 | Yes | No | No | No | Yes | Yes | Yes | Yes | Yes | Yes | Yes | Yes | Yes | No | No | No | Yes |
| Yang Q 2020 | Yes | No | No | No | Yes | Yes | Yes | Yes | Yes | No | Yes | Yes | Yes | No | No | No | Yes |
| Yang S 2020 | Yes | No | No | No | No | Yes | No | Yes | Yes | Yes | Yes | Yes | Yes | No | No | No | Yes |
| Yin ZL 2020 | Yes | Yes | No | No | Yes | Yes | No | Yes | Yes | Yes | Yes | Yes | Yes | No | No | No | Yes |
| Zhang Q 2020 | Yes | No | No | No | Yes | No | Yes | Yes | No | Yes | Yes | Yes | Yes | No | No | No | Yes |
| Zhu JM 2020 | Yes | No | No | No | Yes | Yes | Yes | Yes | Yes | Yes | Yes | Yes | Yes | No | Yes | No | Yes |
| Deng FH 2021 | Yes | No | No | No | Yes | No | Yes | Yes | Yes | Yes | Yes | Yes | Yes | No | No | No | Yes |
| Duan C 2021 | Yes | No | No | No | Yes | No | No | Yes | Yes | Yes | Yes | Yes | Yes | No | No | No | Yes |
| Duan YF 2021 | Yes | No | No | No | Yes | Yes | Yes | Yes | Yes | Yes | Yes | Yes | Yes | No | No | No | Yes |
| Liu LD 2021 | Yes | No | No | No | Yes | No | Yes | Yes | Yes | No | Yes | Yes | No | No | No | No | No |
| Song YJ 2021 | Yes | No | No | No | Yes | No | Yes | Yes | Yes | Yes | Yes | Yes | Yes | No | No | No | Yes |
| Wang ZX 2021 | Yes | No | No | No | Yes | Yes | Yes | Yes | Yes | Yes | Yes | Yes | No | No | No | No | No |
| Wang T 2021 | Yes | No | No | No | Yes | No | Yes | Yes | Yes | No | Yes | Yes | Yes | No | No | No | Yes |
| Zhang L 2021 | Yes | No | No | No | No | Yes | Yes | Yes | Yes | Yes | Yes | Yes | Yes | No | No | No | Yes |
| Zhang SH 2021a | Yes | No | No | No | Yes | Yes | No | Yes | Yes | Yes | Yes | Yes | Yes | No | No | No | Yes |
| Zhang SH 2021b | Yes | No | No | No | Yes | Yes | No | Yes | Yes | Yes | Yes | Yes | Yes | No | No | No | Yes |
| Zhang CX 2021 | Yes | No | No | No | Yes | Yes | No | Yes | Yes | Yes | Yes | Yes | Yes | No | No | No | Yes |
| Huang H 2022 | Yes | No | No | No | Yes | No | Yes | Yes | Yes | Yes | Yes | Yes | Yes | No | Yes | No | Yes |
| Li JX 2022 | Yes | No | No | No | No | No | Yes | Yes | Yes | Yes | Yes | Yes | No | No | No | No | No |
| Ren CY 2022 | Yes | No | No | No | Yes | Yes | Yes | Yes | Yes | Yes | Yes | Yes | Yes | No | No | No | Yes |
| Guo LL 2022 | Yes | No | No | No | No | No | Yes | Yes | Yes | Yes | Yes | Yes | No | No | No | No | No |
| Ma AF 2022 | Yes | No | No | No | No | Yes | No | Yes | Yes | Yes | Yes | Yes | Yes | No | No | No | Yes |
| Zhang QS 2022 | Yes | No | No | No | No | Yes | No | Yes | Yes | Yes | Yes | Yes | Yes | No | Yes | No | Yes |

Note: 1a, style of acupuncture; 1b, reasoning for treatment provided, based on historical context, literature sources, and/or consensus methods, with references where appropriate; 1c, extent to which treatment was varied; 2a, number of needle insertions per subject per session (mean and range where relevant); 2b,points used (unilateral/bilateral); 2c, depths of insertion (e.g., cun or tissue level); 2d, responses elicited (e.g., de qi or twitch response); 2e, needle stimulation (e.g., manual or electrical); 2f, needle retention time; 2g, needle type (diameter, length, and manufacturer or material); 3a, number of treatment sessions; 3b, frequency of treatment; 4a, details of other interventions (e.g., moxibustion, cupping, herbs, exercises, lifestyle advice); 4b, setting and context of treatment, including instructions to practitioners, and information and explanations to patients; 5, Description of participating acupuncturists (qualification or professional affiliation, years in acupuncture practice, other relevant experience); 6a, Rationale for the control or comparator in the context of the research question, with sources that justify this choice; 6b, Precise description of the control or comparator. If sham acupuncture or any other type of acupuncture-like control is used, provide details as for Items 1 to 3 above; No, no details report; Yes, details reported.
